# Reaching Never- and Incompletely-Vaccinated Children with Routine Immunization: A Proof-of-Concept Activity Using Geo-Referenced Microplans in Two Health Zones in Maniema Province, Democratic Republic of the Congo

**DOI:** 10.3390/vaccines14020175

**Published:** 2026-02-13

**Authors:** Mary M. Alleman, Affaud Anais Tanon, Emmanuel Rukengwa, Kevin Tschirhart, Christ Lendo, Merveille Balepukayi, Grace Koko Cishugi, Eddy Balume Shaboya, Chuku Mburugu, Gloire Chasinga, Amy Louise Lang, Katherine Schwenk, Roger Widmer, Stéphane Vouillamoz, Jean Jacques Kanyaka Biduaya, Alain Magazani, John Kaozi, Generose Matunda Sumaili, Serge Sukani, Dolla Ngwanga Lapaba, Kimberly E. Bonner, Robert T. Perry, Jean Crispin Mukendi, Aimé Cikomola Mwana wa bene, Paul Lame

**Affiliations:** 1 Global Immunization Division, Centers for Disease Control and Prevention, Atlanta, GA 30329, USA; aatanon@gmail.com (A.A.T.); voq2@cdc.gov (K.E.B.); rmp9@cdc.gov (R.T.P.); 2 GRID3, Kinshasa BP 7248 Kin 1, Democratic Republic of the Congo; emmanuel.rukengwa@grid3.org (E.R.); christlendo92@gmail.com (C.L.); balepukayimerveille@gmail.com (M.B.); gracecishugi@gmail.com (G.K.C.); balumeeddyshaboya07@gmail.com (E.B.S.); mbmmalyanga@gmail.com (C.M.); gloirechasinga19@gmail.com (G.C.); 3 GRID3, New York, NY 10017, USA; kevin.tschirhart@grid3.org; 4 Center for Integrated Earth System Information (CIESIN), Columbia Climate School, Columbia University, Palisades, NY 10964, USA; 5 DRT Strategies, Arlington, VA 22209, USA; langstervision@gmail.com; 6 Geospatial Research, Analysis and Services Program (GRASP), Agency for Toxic Substances and Disease Registry, Centers for Disease Control and Prevention, Atlanta, GA 30329, USA; tdy9@cdc.gov; 7 Novel-t, 1214 Geneva, Switzerland; roger_widmer@hotmail.com (R.W.); sv@novel-t.ch (S.V.); 8 Bizzell, New Carrollton, MD 20785, USA; jjkbiduaya@googlemail.com; 9 Avram Corporation, Miami, FL 33137, USA; 10 African Field Epidemiology Network (AFENET), Regional Coordination Office for the Central and Indian Ocean Francophone Region, Kinshasa 01207, Democratic Republic of the Congo; amagazani@afenet.net; 11 Expanded Programme on Immunization, Ministry of Public Health, Hygiene, and Prevention, Kinshasa 01206, Democratic Republic of the Congo; john-descemet.kaozi@unikin.ac.cd (J.K.); generosesumaili@gmail.com (G.M.S.); sukaniserge@gmail.com (S.S.); dollangwanga@gmail.com (D.N.L.); mukendijean2@gmail.com (J.C.M.); aimcik@yahoo.fr (A.C.M.w.b.); 12 Mashako Plan, Expanded Programme on Immunization, Ministry of Public Health, Hygiene, and Prevention, Kinshasa 01206, Democratic Republic of the Congo

**Keywords:** Democratic Republic of the Congo, routine immunization, non-vaccination, zero-dose children, never-vaccinated children, incompletely-vaccinated children, microplanning, geo-referenced microplanning, geo-spatial analysis, reasons for non-vaccination, periodic intensification of routine immunization

## Abstract

Background/Objectives: The Democratic Republic of the Congo (DRC) has a history of low coverage (<50%) with all first-year-of-life vaccines for children aged 12–23 months, resulting in frequent outbreaks of vaccine-preventable diseases. In response, the DRC’s Expanded Program on Immunization (EPI) is applying innovations to improve vaccination coverage, including using geospatial data to inform vaccination planning (geo-referenced microplans). This report describes a proof of concept to geo-locate, by locality of residence, never-vaccinated children (NVC) or incompletely vaccinated children (IVC); use those data to prepare geo-referenced microplans for rounds of Periodic Intensification of Routine Immunization (PIRIs); and implement the PIRIs. Methods: In 2022, in Kindu and Kibombo Health Zones (HZs), Maniema Province, DRC, children aged 0–23 months were enumerated with inquiries about their vaccination status and reasons for non-vaccination by locality of residence. The enumeration was coupled with the collection of the localities’ geographic coordinates, facilitating the spatial illustration of estimated proportions of NVC by locality. Coordinates for HZ and health area (HA) landmarks and borders were also collected. We created maps that informed geo-referenced PIRI microplans, placing an emphasis on deploying vaccination teams to localities with high proportions of NVC, especially those in remote and riverine locations. To account for inclusion of children aged up to 59 months in the PIRIs, enumeration data were extrapolated to estimate the numbers of NVC and IVC in this wider age range. Volunteers mobilized communities for the PIRIs, HA staff vaccinated age-eligible children, and vaccination teams were geographically tracked. Results: In Kindu, 29,837 children aged 0–23 months were enumerated in 430 localities; among them, 38% were NVC and 6% IVC. In Kibombo, 9582 children aged 0–23 months were enumerated in 168 localities; among them, 50% were NVC and 16% IVC. In both HZs, reasons for never vaccination were primarily associated with knowledge- or belief-related factors, while reasons for incomplete vaccination were associated with access-related factors. Between HAs and localities, there was heterogeneity in the proportions of NVC and IVC and in the reasons for non-vaccination. The numbers of NVC and IVC aged 0–59 months were estimated at 28,220 and 4613 in Kindu and 12,038 and 3785 in Kibombo. Approximately 2000 health staff and community volunteers were engaged for implementation of each of the three PIRIs. The number of children vaccinated during the three PIRIs ranged from 15,500 to 26,500 and from 10,500 to 15,500 in Kindu and Kibombo, respectively. Data suggest that vaccinated children originated from >90% of localities identified during the cartography. Tracking data showed that vaccination teams visited localities with high proportions of NVC, including those that were remote and riverine. Conclusions: Geo-referenced microplanning with engagement of health staff and communities succeeded in vaccinating at least 40,000 children who were not routinely benefiting from health services in two HZs in the DRC; similar innovative strategies could be considered elsewhere. Applying new technologies to existing microplanning strategies can enhance their success.

## 1. Introduction

The Expanded Programme on Immunization (EPI) in the Democratic Republic of the Congo (DRC) was launched in 1977, targeting children with four vaccines: Bacille Calmette–Guerin (BCG), measles, oral polio vaccine (OPV), and the combined diphtheria, pertussis, and tetanus (DTP) vaccine [1,2,3]. The DRC’s EPI schedule currently includes eight vaccines with administration recommended as described in Supplemental Table S1 (ST1) [4].

Over the decades, the DRC’s EPI program has been monitored through reviews of annual administrative vaccination coverage data and numerous surveys estimating national, provincial, and health zone (HZ)-level vaccination coverage [4,5,6,7,8,9,10,11,12,13,14,15,16,17,18,19,20]. Estimates have revealed numerous years of low coverage insufficient to prevent vaccine preventable disease (VPD) outbreaks of large magnitude and long duration [21,22,23,24,25,26,27,28,29,30,31,32]. Moreover, in the DRC, the national proportion of completely-vaccinated children (CVC, children aged 12–23 months who have received all first-year-of-life vaccines in the EPI schedule within the year being surveyed) only once exceeded 50% as measured through various surveys conducted during the period 1995–2022; during the same period, the proportion of children who had received no first-year-of-life vaccines fluctuated between 6% and 20% (Supplemental Table S2 (ST2)) [9,10,12,13,14,15,16,17,18]. Notably, the proportion of CVC fell from 45% in the 2013–2014 Demographic and Health Survey to 35% in the 2017–2018 Multiple Indicator Cluster Survey. In response to this decline and recurring VPD outbreaks, in 2018 the DRC’s EPI program and immunization partners launched the Emergency Plan for the Revitalization of Routine Immunization (RI) in the DRC (known as the Mashako Plan) [10,15,24,25,28,30,32,33,34,35]. The plan’s second phase (Mashako Plan 2.0) has aimed to reduce the number of children not receiving any routine vaccines in their first year of life [34,35]. One of the four Mashako Plan interventions, “Service Delivery”, advocates for relating vaccination sessions with catchment areas and their populations, also known as microplanning. The basic process of microplanning is detailed in numerous guidelines and strives to ensure that all populations and geographies are reached for health services [33,36,37,38]. New technologies and novel approaches are being applied to strengthen and improve the success of microplanning. For example, geo-referenced microplans incorporate spatial (geographic) data collected through satellite imagery and “on the ground” mapping [39,40,41,42,43,44,45]. One initiative in the DRC, the GRID3 Mapping for Health (GRID3-M4H) project, began in 2020 with the objective of improving RI services using geo-referenced microplans [39,40,41,42]. With the participation of local health staff, GRID3-M4H project staff have generated population estimates, identified and collected geographic coordinates for settlements and critical infrastructure, revised HZ and health area (HA) borders, and used these data to propose locations for vaccination sites convenient for target populations. The initial GRID3-M4H projects were implemented in Kinshasa, Kasai, Kasai Oriental, Haut Katanga, and Lomami provinces.

**Figure 1:**
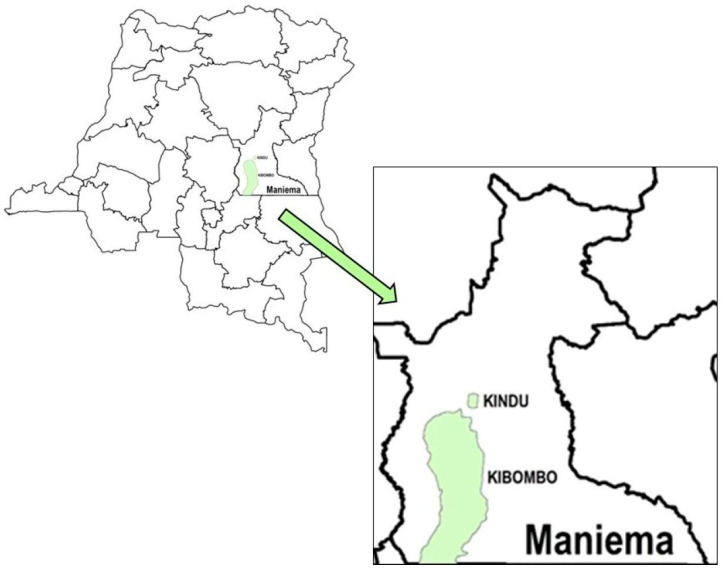
Map of the Democratic Republic of the Congo (**left**), with a guiding arrow to the inset (**right**) illustrating the location of Kindu and Kibombo Health Zones (in green shading) within Maniema Province.

The activity described in this report builds upon the experience of the GRID3-M4H project and was conducted in two HZs, Kindu and Kibombo, in Maniema Province, DRC (Figure 1). Maniema Province and these HZs were chosen because of documented poor vaccination coverage (Supplemental Table S2 (ST2)), high proportions of “zero-dose” children (defined as those who did not receive any dose of a DTP-containing vaccine), and a history of polio and measles outbreaks [9,10,13,14,15,16,17,18,19,22,23,24,25,26,27,28,29,30,31,32]. In surveys conducted during 2001–2022, Maniema was documented as having up to 59% of children aged 12–23 months who had never received any first-year-of-life vaccines (Supplemental Table S2 (ST2)) [9,10,13,14,15,16,17,18,19]. We adopted the objectives of the GRID3-M4H project and added several more in order to (1) collect coordinates for HZ and health area (HA) landmarks and borders; (2) enumerate (rather than estimate) and geo-locate, by locality of residence, children aged 0–23 months who were never vaccinated (NVC) or incompletely vaccinated (IVC) through routine health services, while also documenting the predominant reasons for non-vaccination; (3) use those data to prepare geo-referenced microplans for rounds of Periodic Intensification of Routine Immunization (PIRIs); and (4) vaccinate enumerated children during PIRIs according to the microplans [39,40]. This activity was aligned with numerous national strategic immunization plans and the ambitions of global partners to identify and reach “zero-dose” children, including the global “Big Catch-up” initiative [46], and contributed to GRID3′s objective to map Maniema Province, including updating HZ and HA borders [19,36,47,48,49].

## 2. Materials and Methods

### 2.1. Implementing Partners

This activity was designed and implemented as a collaborative effort. Implementing partners included the DRC-EPI Programme (national and provincial), GRID3, the African Field Epidemiology Network, and the Centers for Disease Control and Prevention (CDC, Atlanta).

### 2.2. Public Health Structure, Maniema Province, DRC

**Figure 2:**
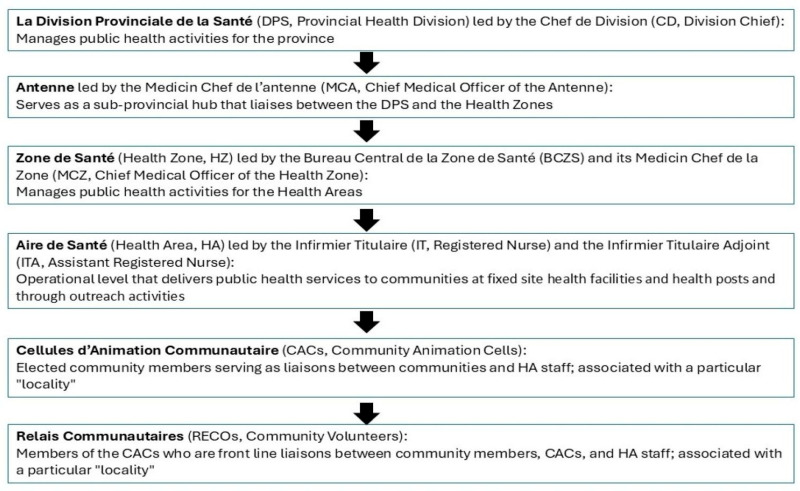
Maniema Province, Democratic Republic of the Congo: Schematic of the provincial public health structure. Arrows indicate the flow of the structure from the provincial level to the intermediary levels and then to the community.

The functional public health structure and its link to communities in Maniema Province are described briefly in Figure 2 [43,50,51]. Kindu and Kibombo HZs have 11 and 12 HAs, respectively. For this activity, HAs were divided into smaller areas, hereafter referred to as “localities”, served by Cellules d’Animation Communautaire (CACs); localities are synonymous with neighborhoods within urban areas or individual villages in rural areas. Staff from the Division Provinciale de la Santé (DPS), Antenne, Bureau Central de la Zone de Santé (BCZS), and HAs and members of the CACs (particularly the Relais Communautaires, RECOs, community volunteers) participated in this activity (Figure 2). Kindu and Kibombo HZs were chosen for reasons described in the introduction as well as their accessibility, their urban (Kindu) and rural (Kibombo) environments, and the presence of remote rural and riverine (Congo River) communities [16,17,18,24,28]. Kindu is the provincial hub, with proximity to provincial administration, an airport, and a vaccine depot.

### 2.3. Participatory Mapping, July–October 2022

Along with the enumeration of children aged 0–23 months (described in detail below), participatory mapping was conducted as documented elsewhere (see Graphical Abstract) [39,40,41,42]. Briefly, four GRID3 cartographers (two each in Kindu and Kibombo) trained and collaborated with the Registered Nurses and Assistant Registered Nurses [Infirmiers Titulaires (ITs) and Infirmiers Titulaires Adjoints (ITAs) respectively (hereafter referred to as “nurses”)] from each HA to collect geospatial data in their HAs on all inhabited settlements/localities, HA geographic boundaries, health facilities, schools, religious centers, etc. While nurses collected data in their respective HAs, GRID3 cartographers worked with BCZS staff to discuss HA and HZ boundaries using available maps and high-resolution satellite imagery. Once geospatial data collection was completed in the HAs and data were analyzed and integrated into geospatial layers, an initial validation was conducted with the BCZS. Subsequently, GRID3 cartographers conducted final data analyses; final basemaps were reviewed with BCZS staff and made available publicly at https://grid3.org/geospatial-data-drc (accessed on 4 February 2026) [39,41,42,52].

### 2.4. Enumeration of Children Aged 0–23 Months and Determination of Vaccination Status, in Conjunction with Participatory Mapping, July–October 2022

A house-to-house enumeration of children aged 0–23 months, done simultaneously with the participatory mapping (described above), was conducted in Kindu and Kibombo during July–October 2022 (see Graphical Abstract) [39,40,41,42]. The enumeration was organized geographically by known CACs and their localities; RECOs from each CAC conducted the enumeration in all houses with age-eligible children within their locality. RECOs used a paper enumeration questionnaire (in French and Swahili) on which they recorded, for each child aged 0–23 months, data on the child’s demographics, vaccination status, and reasons for non-vaccination, if applicable (Tool S1A−C). Field tools were provided to assist RECOs in determining a child’s approximate age (Tool S2A,B) and the vaccines for which the child was age-eligible (Tool S3A,B). RECOs and their supervisors were trained on the DRC’s EPI schedule, how to use the age guide, and how to complete the enumeration questionnaire; supervisors were provided with a written guide detailing how to complete the enumeration questionnaire (Tool S4A,B). After classroom training, RECOs and supervisors participated in field exercises on tool use. The enumeration was supervised by staff from the DPS, BCZS, and HAs, GRID3 cartographers, and graduates of the DRC’s Field Epidemiology Training Program.

Vaccination status (vaccinated vs. never vaccinated) was assessed by vaccination card or caretaker verbal declaration of (1) at least one vaccination received through routine health services at a clinic or hospital or (2) a scar on one forearm (preferably the recommended left), indicating receipt of the BCG vaccine. If the answer was no to both inquiries, the child was considered a never-vaccinated child (NVC). If the answer was yes to either of these inquiries, the child was considered to have been vaccinated. To assess vaccination completion [a completely-vaccinated child (CVC) vs. an incompletely-vaccinated child (IVC)], the vaccination card (preferably) or verbal declaration by the caretaker to the RECO was then used to determine if the child had received all recommended vaccinations according to age. If the answer was yes, the RECO noted the child as a CVC; if no, the RECO noted the child as an IVC. Reference to CVC and IVC always implies “according to the child’s age” at the time of the 2022 enumeration. Principal reasons for non-vaccination (never and incomplete) included the following pre-specified categories: knowledge- and belief-related (do not know or have forgotten vaccination schedule, not aware of the benefits of vaccination, fear of adverse events after vaccination, organized hesitancy towards vaccination, or individual refusal); access-related (vaccination site is too far away, absence of transport, lack of time to bring the child, long waits at vaccination sites, or lack of money); or other. The RECO recorded the child’s vaccination status (CVC, IVC, or NVC) as well as the principal reason for incomplete or never vaccination on the enumeration questionnaire (Tool S1A−C).

Once RECOs completed enumeration in their specific localities, the HA nurses visited each locality within their HA, collected the paper copy enumeration questionnaires, manually aggregated the questionnaire data for that particular locality, and entered the locality’s aggregated data into an Open Data Kit [ODK, https://opendatakit.org/software/ (accessed on 4 February 2026), San Diego, CA, USA] questionnaire on a smartphone provided by GRID3. After data entry on the smartphone, the HA nurses recorded geographic coordinates in a central location of the locality, thereby geo-linking the enumeration data with the locality. GRID3 cartographers subsequently collected the smartphones and uploaded the data to Novel-t’s Geospatial Tracking System (GTS) [https://www.novel-t.ch/home (accessed on 4 February 2026), Geneva, Switzerland] from where the data could be subsequently downloaded in Excel (Microsoft, Redmond, WA, USA) for management and analyses.

### 2.5. Analysis of Enumeration Data and Linking to Geospatial Data, November 2022–June 2023

During November 2022–June 2023, the linkages between the enumeration and geospatial data were verified, and analyses were conducted in Excel to calculate the proportion of enumerated children who were NVC, IVC, or CVC, by HZ, HA, and locality. For the NVC and IVC, the reasons for non-vaccination were analyzed by (1) aggregated knowledge-/belief-, access-, and other-related categories and (2) by the individual pre-specified reasons described above. Large format HA maps (90 cm × 110 cm) illustrating the proportion of NVC by locality and the principal reason for never vaccination were prepared, printed, and distributed to relevant health staff; examples of maps are presented in the Results.

### 2.6. PIRI Geo-Referenced Microplanning and Implementation, July 2023–January 2024

During July–September 2023, geo-referenced microplanning for three rounds of PIRIs was conducted (see Graphical Abstract) [36,39,44,53,54]. Staff at all levels of the provincial public health structure and community members participated in the process, which included discussions of the 2022 participatory mapping and enumeration results and how they could serve as the basis for HA geo-referenced microplans; data illustrated on the large format HA maps were integrated into planning. The DPS requested to conduct the PIRIs for children aged 0–59 months, a wider age range than enumerated. We, therefore, extrapolated the 0–23 month enumerated population data by locality to estimate the number of NVC and IVC aged 0–59 months. The 0–23 month enumerated figures were divided by two to obtain an estimate per one-year birth cohort; this estimate was subsequently multiplied by five for an estimate for the five one-year birth cohorts in the 0–59-month age range. Lists of these estimates by locality were prepared, printed, and distributed to HA staff for use during microplanning (an example for Kindu HZ is Tool S5).

Following global guidelines for catch-up vaccination, the HA nurses completed the geo-referenced microplanning using the estimates of NVC and IVC aged 0–59 months and their localities of residence to identify and quantify the logistics (e.g., vaccine requirements, human resources, cold chain, vaccination team transport, etc.), social mobilization, training, and financing needed for each of the three PIRIs during which all age-appropriate vaccines in the DRC’s EPI schedule were to be offered to those aged 0–59 months who had not received them (Supplemental Table S1 (ST1), Tool S6A,B) [53,54]. Special attention was given to planning for vaccination team deployment to localities with high proportions of NVC. In the days prior to each PIRI, RECOs were engaged by their CACs to mobilize their populations and inform them about the PIRI dates, vaccination site locations, and the EPI schedule. To assist the RECOs in mobilizing the NVC and IVC identified in 2022, the paper copy enumeration questionnaires listing these children by name and locality were made available. As RECOs went house-to-house for mobilization, they used the EPI schedule graphic and an updated age guide to assist in identifying children aged 0–59 months who needed vaccination (Tools S3A,B and S7A,B). RECOs completed a coupon (Tool S8A,B) for each child aged 0–59 months in the house noted to be lacking an age-appropriate vaccine, recording the child’s name, age, and incompletely- or never-vaccinated status. Upon the child’s arrival at the vaccination site, the coupon was collected as preliminary information about the child’s vaccination status.

The three PIRIs were conducted over five to seven days each in September 2023, November 2023, and January 2024. The September PIRI was conducted in conjunction with a measles Supplementary Immunization Activity (SIA) targeting children aged 6–59 months [38]. Vaccination teams were composed of a medical professional(s) trained on the DRC’s EPI schedule and in the procedures for preparing and administering vaccines to the target age group (Tools S3AB, S6A,B and S9A,B). In addition, teams had a person(s) with the following responsibilities: (1) completion of paper data collection forms (Tally Sheets, Tool S10A,B) that tallied the number of doses of each vaccine administered to children aged 0–11 months, 12–23 months, and 24–59 months; (2) completion of an official DRC-EPI program vaccination card for children aged 0–23 months or a post-vaccination coupon for those aged 24–59 months (Tool S11A,B); (3) completion of a form (“provenance form”) that registered, for each child who arrived at the vaccination site, their age, locality of residence, and whether they had ever been vaccinated before the PIRI (Tool S12A,B); and (4) entry of some of these data, summarized by day or by completion of a vaccination session in a particular locality, into a smartphone-based ODK questionnaire that also recorded the geographic coordinates of the vaccination site [44]. A guide on how to organize vaccination sites and an operational guide were provided (Tools S9A,B and S13A,B). In addition to being entered into a smartphone, as a back-up and to facilitate quick accounting of daily accomplishments during each PIRI, data from the vaccination tally and “provenance” forms were aggregated daily for the HA by HA nurses, recorded on paper summary forms that were sent to the BCZS, and subsequently entered into Excel by BCZS staff; the BCZS staff, in their turn, entered the HZ’s daily aggregated data on paper summary forms that we sent to the provincial level (Tools S14A–S15B).

During the PIRIs, teams vaccinated at accessible fixed sites (such as health facilities, health posts, hospitals, schools, churches, etc.) or as mobile teams that traveled by motorcycle, boat, or on foot to rural, remote, and riverine localities, establishing temporary vaccination sites. Supervisors were deployed and used a smartphone-based supervisory questionnaire (Tool S16A,B) to record observations and geographic coordinates at each supervised site.

After each PIRI, data from the smartphones were uploaded to the GTS website and were thereafter available in Excel for analysis. Vaccination team tracking data could be visualized on the GTS website for review of localities visited. After each PIRI, review meetings were held with all participants to review (1) vaccination team accomplishments relative to the estimated numbers of children needing vaccination by locality, (2) the geographic positioning of teams, (3) the locality of residence of children coming to the vaccination sites, and (4) the success of teams in vaccinating at localities that were remote or riverine and those with high proportions of NVC. Strengths and weaknesses in implementation were discussed to facilitate improvements during subsequent PIRIs.

### 2.7. Analysis of PIRI Data, November 2023–May 2024

During November 2023–May 2024, smartphone and Excel data on the number of doses of each vaccine administered by age group and by day of the three PIRIs were analyzed in Excel to calculate, by HZ, HA, and team, the total number of children and the total by age category vaccinated with each vaccine offered. Data from the “provenance forms” were used to analyze the locality of residence for participating children (see Graphical Abstract). Data from the geographic tracking of vaccination teams were used to analyze the locations of vaccination sessions for comparison with plans and to evaluate the length of time at vaccination sites and arrival at remote and riverine localities.

## 3. Results

### 3.1. Participatory Mapping, July–October 2022

**Figure 3:**
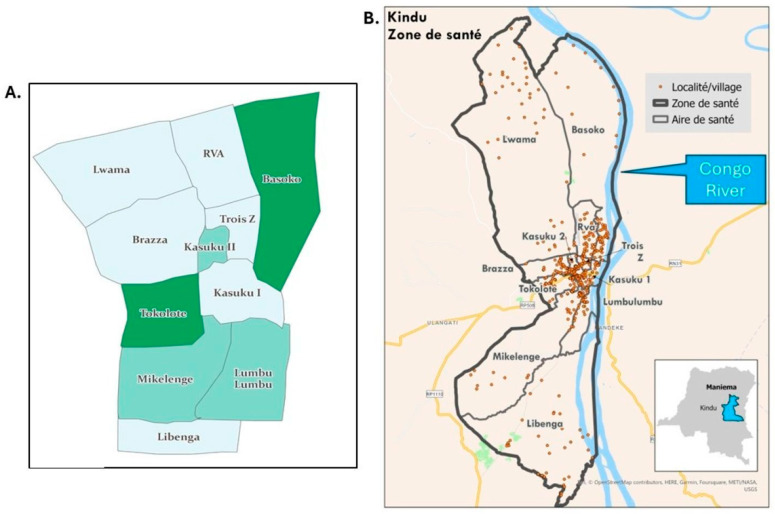

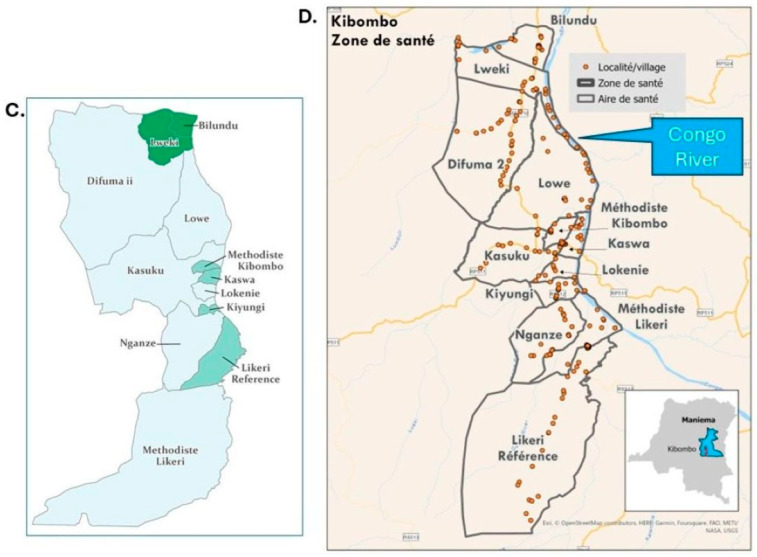
(**A**,**B**) Kindu Health Zone (HZ, Zone de santé), (**C**,**D**) Kibombo HZ, Maniema Province, Democratic Republic of the Congo. (**A**,**C**) Known HZ and health area (HA, Aire de santé) boundaries prior to October 2022 (shades of green in these maps are for visual purposes only) and (**B**,**D**) localities (brown dots) identified during participatory mapping with revised HZ and HA geographic boundaries, as of October 2022.

Figure 3 provides maps illustrating the HZ and HA boundaries of Kindu (Figure 3A,B) and Kibombo (Figure 3C,D) based on geospatial data available before (Figure 3A,C) and after (Figure 3B,D) the 2022 participatory mapping. These figures illustrate significant revisions in HA shapes, borders, and geographic extent. Figure 3B,D also illustrate the geographic location of the 430 and 168 identified inhabited localities of Kindu and Kibombo, respectively. The number of localities by HA ranged from 15 (Trois Z) to 57 (Tokolote) in Kindu and from 4 (Bilundu) to 23 (Difuma 2) in Kibombo (Supplemental Table S3A,B (ST3A,B)). Maps that also include the location of HA health facilities can be found at https://grid3.org/geospatial-data-drc (accessed on 4 February 2026).

### 3.2. Enumeration of Children Aged 0–23 Months and Determination of Vaccination Status, in Conjunction with Participatory Mapping, July–October 2022

#### 3.2.1. Kindu HZ

**Figure 4:**
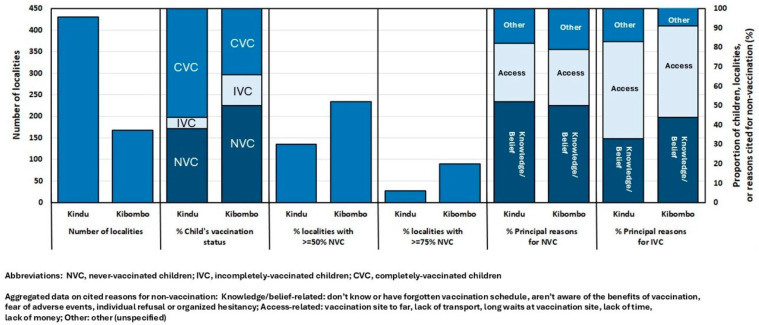
Kindu and Kibombo Health Zones, Maniema Province, Democratic Republic of the Congo: Summary of enumeration of children aged 0–23 months with vaccination status, principal reasons for non-vaccination, and participatory mapping, July–October 2022.

In Kindu HZ, the enumeration with participatory mapping revealed a total of 29,837 children aged 0–23 months living in the 430 identified localities (Supplemental Table S3A (ST3A), Figure 3B and Figure 4). At the HZ level, the numbers of NVC and ICV were 11,288 (38%) and 1845 (6%), respectively (Supplemental Table S3A (ST3A), Figure 4). The proportions of NVC and IVC ranged from 15–61% and from 2–16%, respectively, in the various HAs (Supplemental Table S3A (ST3A)). Kindu HZ had 30% and 6% of localities with ≥50% NVC and ≥75% NVC, respectively (Supplemental Table S3A (ST3A), Figure 4). In Kindu HZ, overall and with data on reasons for non-vaccination aggregated, the most frequently cited reasons by caretakers for non-vaccination among NVC were knowledge- or belief-related (52%) while the same among IVC were access-related (50%) (Figure 4). When data were disaggregated by specific reason and summarized for the HZ, the most frequently cited reasons for non-vaccination among NVC were equally (21%) lack of time to bring the child for vaccination and individual refusal to vaccinate the child, with fear of adverse events and “other” frequently expressed [Supplemental Figure S1A (SF1A)]. Among Kindu’s HAs, the most frequently cited reasons for non-vaccination of NVC varied; however, lack of time to bring the child, individual refusal to vaccinate, fear of adverse events, and “other” were commonly cited reasons (Supplemental Figure S1A (SF1A)). Of note, in Brazza and Kasuku 1 HAs, organized hesitancy and individual refusals, respectively, were the principal reasons cited for NVC. The most frequently cited reason for non-vaccination of IVC was lack of time to bring the child in Kindu HZ overall and in seven of the 11 HAs (Supplemental Figure S1B (SF1B)).

#### 3.2.2. Kibombo HZ

In Kibombo HZ, the enumeration with participatory mapping revealed a total of 9582 children aged 0–23 months living in the 168 identified localities (Supplemental Table S3B (ST3B), Figure 3D and Figure 4). At the HZ level, the numbers of NVC and IVC were 4815 (50%) and 1514 (16%), respectively (Supplemental Table S3B (ST3B) and Figure 4). The proportions of NVC and IVC ranged from 28–78% and from 4–33%, respectively, in the various HAs (Supplemental Table S3B (ST3B)). Kibombo HZ had 52% and 20% of localities with ≥50% NVC and ≥75% NVC, respectively (Supplemental Table S3B (ST3B), Figure 4). Bilundu HA had 100% of localities with ≥50% NVC, and 64% of Kasuku HA’s localities had ≥75% NVC (Supplemental Table S3B (ST3B)). In Kibombo HZ overall, and with data on reasons for non-vaccination aggregated, the most frequently cited reasons by caretakers for non-vaccination among NVC were knowledge- or belief-related (50%) while the same among IVC were access-related (47%) (Figure 4). When the data were disaggregated by specific reason and summarized for the HZ, the most frequently cited reasons for non-vaccination of NVC were not being aware of the benefits of vaccination and “other”; fear of adverse events, lack of time to bring the child for vaccination, and individual refusal were frequently expressed (Supplemental Figure S2A (SF2A)). Among Kibombo’s HAs, the most frequently cited reasons for non-vaccination of NVC varied; however, in four of 12 HAs, the most frequently cited reason was a lack of awareness of the benefits of vaccination. Overall, in Kibombo, the most frequently cited reason for non-vaccination of IVC was lack of time to bring the child for vaccination; in six and five of Kibombo’s 12 HAs, lack of time and fear of adverse events were the most frequently cited reasons, respectively (Supplemental Figure S2B (SF2B)).

#### 3.2.3. Linking Enumeration and Geospatial Data

**Figure 5:**
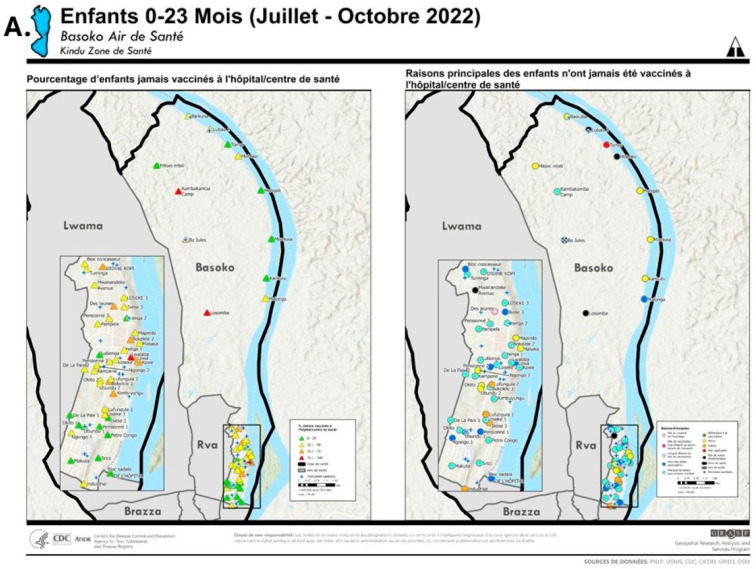

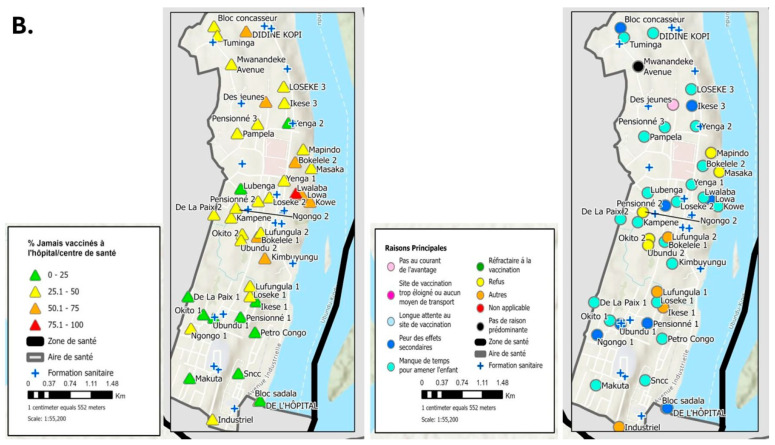
(**A**) Basoko Health Area (HA, Aire de santé), Kindu Health Zone (Zone de santé), Maniema Province, Democratic Republic of the Congo, is an example of a combination map provided to HA staff that illustrates, by locality, the proportion of children aged 0–23 months who were never vaccinated in a hospital or health center (left) and the principal reasons for never vaccination as reported by caretakers (right), 2022. The proportions of children, by locality, who were never vaccinated are presented with colored triangles as follows: green, 0–25%; yellow, 25.1–50%; orange, 50.1–75%, and red, 75.1–100%. The principal reasons for never vaccination, by locality, are presented with colored circles as follows: light pink, not aware of the benefits; dark pink, vaccination site is too far or no means of transport; light blue, long waits at the vaccination site; dark blue, fear of adverse events; aqua, lack of time to bring the child; dark green, organized refusal to vaccinate; yellow, individual refusal to vaccinate; orange, other; red, not applicable; black, no predominant reason. The blue crosses represent the location of health facilities. These legends are the same for Tools S17–S21. (**B**) Magnified insets from maps in Figure 5A.

After linking the participatory mapping and enumeration data, maps were prepared illustrating the proportion of NVC and the principal reason cited for non-vaccination of NVC by locality as “combination maps” (Figure 5 provides the example of Basoko HA, Kindu.). This map illustrates the locality-to-locality variations in the proportions of NVC and the principal reasons for non-vaccination among NVC. Moreover, it assists in visualizing localities with ≥75.1% of NVC, as also observed in the north of Lwama HA, Kindu, and along the Congo River in Lowe HA, Kibombo (Tools S17–S19). In addition, localities where individual refusals and organized hesitancy towards vaccination exist can be seen, as also observed in Kasuku 1 HA and Brazza HA, Kindu (Tools S17, S20, and S21). Such maps, prepared for all HAs, were instrumental during the preparation of the geo-referenced microplans for the PIRIs.

### 3.3. PIRI Implementation, September 2023–January 2024

**Figure 6:**
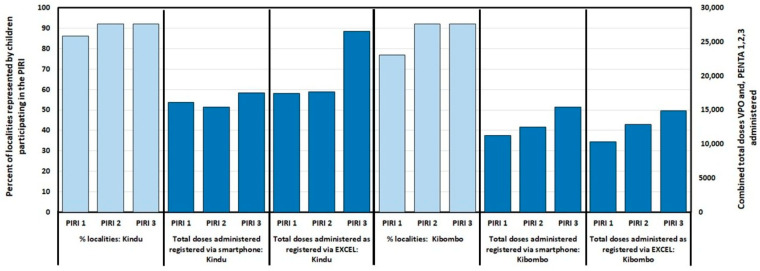
Kindu and Kibombo Health Zones, Maniema Province, Democratic Republic of the Congo: By the round of Periodic Intensification of Routine Immunization (PIRI) conducted in 2023/2024, and data entry method where appropriate, the percentage of localities represented by participants aged 0–59 months, and the combined number of administered doses of oral polio birth dose vaccine and pentavalent vaccines 1, 2, and 3.

**Figure 7:**
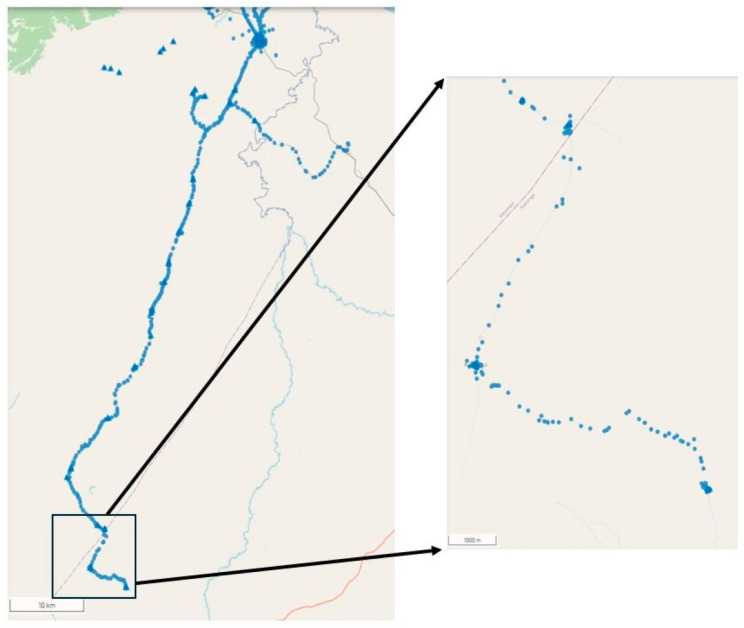
Likeri Reference Health Area (HA), Kibombo Health Zone, Maniema Province, Democratic Republic of the Congo: Geographic tracking of vaccination teams conducting vaccination throughout the HA on a given day during the second of three PIRIs, December 2023 (left panel, scale = 10 km). The blue dots represent moments when geographic location data were recorded on the smartphones carried by vaccination teams and the blue triangles represent when and where those teams entered vaccination session-related data into the smartphones. The right panel inset (scale = 1000 m) is a magnified view of the southernmost region of the HA.

The PIRIs targeted an estimated 28,220 and 12,038 NVC and 4613 and 3785 IVC, aged 0–59 months, in Kindu and Kibombo, respectively, with all vaccines in the routine schedule according to age eligibility (Supplemental Table S3A,B (ST3A,B) and Tool S6A,B). Approximately 2000 health staff and community volunteers participated in each of the three PIRIs (Supplemental Table S4 (ST4)). The number of children arriving at the vaccination site and their localities of residence and vaccine doses administered were recorded by two data entry methods (smartphone or manually through Excel) that yielded differing results (Supplemental Table S5A,B and Figure 6). The number of doses of OPV birth dose (OPV0), first dose (PENTA1), second dose (PENTA2), and third dose (PENTA3) pentavalent vaccine are presented because these 4 vaccines would not have been administered to the same child during a given PIRI; their combined doses are a rough estimate of the number of individual children vaccinated by PIRI. Data varied by the two collection methods. In Kindu, approximately 18,000–32,500 were registered on the provenance forms and 15,500–26,500 were vaccinated (Supplemental Table S5A (ST5A)). Approximately 9500–17,000 were registered on the provenance forms, and 10,500–15,500 were vaccinated during the PIRIs in Kibombo (Supplemental Table S5B (ST5B)). Except for the first PIRI, provenance form data indicate that children from >90% of identified localities in both HZ came to the vaccination sites. No serious adverse events were reported. During all PIRIs, vaccination teams were geographically tracked via smartphones. Tracking data showed that vaccination teams visited localities with high proportions of NVC. The tracks of the vaccination teams working in Likeri Reference HA, Kibombo, on a day during the second PIRI are provided in Figure 7 as example (Tool S22 provides a combination map for this HA for orientation). In this example, tracking ensured that teams arrived at their assigned localities and sites and remained in each for a duration of time appropriate for administration of the vaccinations reported by the team. Moreover, tracking revealed that a team reached the southernmost localities of the HA, some with >50% of children never vaccinated.

## 4. Discussion

Since at least 2020, the DRC has been among the ten countries with the largest numbers of “zero-dose” children (estimated at 839,000 in 2023), and Maniema Province has one of the highest proportions of such children in the country [16,17,18,19,55,56,57,58]. In a recent publication, Ingle and colleagues state: “The challenge ahead lies in identifying zero-dose children and vaccinating them, which requires gaining a deeper understanding of how to reach these communities first” [55]. Aligned with that challenge, the objectives of the GRID-M4H initiative, and the commentary of others that local contexts should be understood prior to designing interventions for vaccination, the activity presented here was a “proof of concept” aimed at contributing to the evidence base for the feasibility of using technology to geo-locate NVC and IVC at the most local level and subsequently using geo-referenced microplans to vaccinate them [19,36,39,40,41,42,43,44,45,55].

GRID3′s participatory mapping process was used to assign precise geographic coordinates to identified inhabited localities and HZ and HA borders for Kindu and Kibombo using data collected “on the ground”, for the first time, by local staff [39,40,41,42]. Previously, borders were approximated with available, but incomplete, data, and local HA staff used hand-drawn maps based upon their experiences and travels. HA borders had been associated with landmarks, such as lakes, rivers, mountains, roads, and villages. While landmarks were useful as operational boundaries and could be placed on hand-drawn maps, knowing their correct spatial placement and relationship with other landmarks requires the collection and analysis of geographic coordinates. In the DRC, precise geographic coordinates for HZ and HA boundaries are gradually being collected because tools for easily capturing coordinates (such as smartphones) have become more readily available, as have resources to build in-country technical capacity for cartographic analysis [42,52].

Combining house-to-house enumeration with inquiries regarding children’s vaccination status and participatory mapping, 168 and 430 localities with 9582 and 29,837 children aged 0–23 months were identified in Kibombo and Kindu, respectively (See Graphical Abstract). The enumeration revealed that 50%, 16% and 34% of children aged 0–23 months in Kibombo and 38%, 6% and 56% of the enumerated children in Kindu were either NVC, IVC, or CVC, respectively; significant heterogeneity was observed among HAs in these two HZs for these categories. While direct comparisons should not be made because of differing methodologies, age groupings, and definitions of vaccination status, annual vaccination coverage surveys conducted by the DRC’s Kinshasa School of Public Health (KSPH) revealed similar general trends in that “complete coverage” (defined as children aged 12–23 months having received each of 13 key antigens) and “children having received no vaccine” ranged from 4.3–35.0% and 20.2–80.3%, respectively, for these two HZs in 2020–2022 [16,17,18]. These KSPH HZ-level findings are consistent with those at the Maniema provincial level where “complete coverage” and “children having received no vaccine” ranged from 7–29% and 28–59%, respectively, during 2017/18–2022 [15,16,17,18]. As was observed in Kibombo, a predominantly rural HZ, other studies have shown an association between a high prevalence of children who have received no first-year-of-life vaccines or are “zero dose” and habitation in the DRC’s more rural environments compared with children who live in urban areas [9,10,12,13,14,15,16,17,18,19,43].

Linking a locality’s geographic coordinates with its proportions of NVC and the reasons for never vaccination enabled detailed spatial analyses, including the use of printed maps that informed geo-referenced PIRI microplanning and contributed to local knowledge [36,39,40,41,42,43,44,45]. HAs in Kindu and Kibombo had localities with ≥50% NVC scattered among localities with lesser proportions. While some clustering of localities with ≥75% NVC was observed in riverine and remote/rural localities, such localities were also observed scattered throughout HAs, including in Kindu’s and Kibombo’s urban centers. In 2022, Mutwadi et al. assessed the spatial distribution of “zero-dose” children in the two HZs of Kikwit City in western DRC [43]. Their methods included collecting the vaccination status and household geographic coordinates for a sampling of children aged 12–23 months in each HA of the HZs. Their overall findings were consistent with those of Kindu and Kibombo in that the proportion of “zero-dose” children varied between HZs, between HAs and communities therein, and that geographic “hotspots” with high proportions of zero-dose children existed.

In this activity, inquiries to caretakers on reasons for non-vaccination (never and incomplete) were not designed to be a comprehensive evaluation; they were meant to provide locality-specific information for the PIRI geo-referenced microplanning and to inform future strategies to improve vaccination coverage [16,17,18,19,43,55,59,60,61,62,63]. When data on caretakers’ reasons for non-vaccination were aggregated for each Kindu and Kibombo, knowledge- or belief-related factors seemed to be associated with never vaccination and access-related factors seemed to be associated with incomplete vaccination. When data were disaggregated by specific reason for non-vaccination, more specificity was revealed for each HZ, HA, and locality. For example, the data revealed that caretakers in Kibombo, more so than in Kindu, were not always knowledgeable about the benefits of vaccination, and for certain HAs in Kibombo, distance to the health facility was a constraint. The findings from this activity are consistent with barriers to vaccination cited in other recent studies conducted at the Maniema provincial level and in other DRC provinces and resemble reasons reported from other countries [16,17,18,19,43,59,60,61,62,63]. Spatial analyses using maps produced during this activity revealed that the predominant reasons for never vaccination frequently differed from locality to locality; however, some geographic clustering of localities was observed where refusals of vaccination (individual and organized) were common and where transport and distance barriers were cited. Other studies have revealed that reasons for never vaccination and incomplete vaccination can differ, in and of themselves, and from locality to locality, necessitating locally tailored interventions to improve vaccination [43,59,61,63].

The geo-localization of NVC and IVC in Kindu and Kibombo was critical for their specific and prioritized inclusion in the geo-referenced PIRI microplanning and implementation [39,42,54]. HA maps were integrated into the PIRI geo-referenced microplanning process that included participation of RECOs, CAC members, and health staff from all levels within the province. To tackle the caretaker-cited constraint of lack of transport and long distances to vaccination sites, microplans included provisions for mobile vaccination team deployment to specific localities with high proportions of NVC, especially those that are riverine, remote, or otherwise frequently without access to health services. Other caretaker constraints were addressed by planning PIRIs for a period of 5–7 days during daylight hours, including weekends, and having vaccination sites at convenient locations, such as at health facilities, hospitals, churches, markets, etc. RECOs were trained on the DRC’s EPI schedule, the importance of vaccination, and necessary actions in the event of adverse events before their discussions with caretakers during door-to-door sensitization of their populations before each PIRI; RECOs were compensated for their efforts. The original paper copy enumeration questionnaires from 2022 listing unvaccinated children by name were available to HA staff to assist RECOs with PIRI mobilization. This “proof of concept” was designed to reduce as many vaccination system constraints as possible by providing resources, including transport, for supervisors and vaccination team members and ensuring that sufficient vaccines, injection supplies, and cold chain equipment were readily available for the PIRIs.

Data collected during the three PIRIs indicate that in Kindu a range of approximately 15,500–26,500 children aged 0–59 months were vaccinated in the PIRIs, representing a range of 86–92% of identified localities, and in Kibombo a range of approximately 10,500–15,500 children aged 0–59 months were vaccinated in the PIRIs, representing a range of 77–92% of identified localities. The smartphone and Excel reporting formats for vaccine doses administered provided differing figures. Moreover, the numbers of children recorded on the “provenance forms” did not always correspond to the numbers of unique individuals vaccinated as they should have. It is not possible to know which figures are the most accurate; thus, they are reported as ranges and considered approximations. That said, it seems that >50% of the estimated number of the NVC and IVC aged 0–59 months participated in the PIRIs. Determining which data most correctly reflect the vaccine doses administered and the localities represented would require a detailed investigation, including reviews of the original paper copy vaccination tally and “provenance” forms. The occurrence of such discrepancies might have been mitigated in “real time” by having more stringent data quality measures in place during implementation. For example, double entry of data into smartphones and Excel spreadsheets and mandatory “two-person” cross-checking of data manually aggregated on paper summary forms. As technology and internet availability advance in the DRC, it may be possible to incorporate automated data quality checks into handheld data collection devices; in this way, high-quality data would be available for review and correction in “real time”.

Vaccination coverage by antigen for the PIRIs was not calculated because, while data for numerators exist (approximations of children vaccinated by antigen), denominators indicating numbers of children aged 0–59 months eligible for each antigen were unknown. Because individual children were not tracked during the enumeration and subsequent vaccination phase, it is not possible to know if the children enumerated in 2022 were among those vaccinated in 2023/24. An electronic and linked birth and vaccination registry would greatly benefit the DRC’s EPI program and would have been instrumental in tracking individual children in this activity [19,55].

Analyses of vaccination team tracking data enabled the spatial visualization of localities visited by teams to assess adherence to microplans and whether teams remained in localities for sufficient time to accomplish reported vaccinations; these analyses were a basis for corrective actions for subsequent PIRIs [43,44,64,65]. Routine immunization and vaccination campaign guideline documents recommend supervision of vaccination teams to ensure the quality of their work and to ensure that they reach their assigned populations and geographies [36,37,38]. Geographic tracking is a means of using technology to facilitate the supervision of many more teams over a greater number of hours and distances than could be accomplished by deploying staff. However, due to technological limitations in Maniema, vaccination team tracking could not be monitored in “real time”; analyses of tracking data and subsequent feedback to supervisors and teams were only possible after each PIRI when data could be uploaded from smartphones to a server. Taken together, data from the “provenance forms” and from the team tracking suggest that children from most localities, including those remote and riverine, were present at vaccination sites during the PIRIs. Tracking of vaccination teams in Chad, Nigeria, and Pakistan during polio SIAs has proven to improve SIA quality, including geographic reach [44,45,64,65,66].

Implementation of this “proof of concept” project faced additional challenges. The accurate assignment of a child’s vaccination status without documentation, such as vaccination cards that many children did not have (Supplemental Table S6 (ST6)), can be difficult, leading to inaccuracies in proportions of NVC and IVC [19,43]. Additional field training on (1) the use of the questionnaires used for enumeration, recording vaccine doses administered, and the locality of residence of children present at vaccination sites during the PIRIs, and (2) data entry into smartphones and Excel would likely have reduced time for data cleaning and improved data accuracy. More in-depth engagement with community and religious leaders in the localities with high proportions of NVC associated with individual refusals and organized hesitancy might have benefited children during the PIRIs; maps with these data remain in the HAs for future exploration and effort around this issue [19,55,60]. The age range (0–59 months) and vaccination status of children targeted for the PIRIs were based upon extrapolation rather than enumeration, possibly resulting in inaccurate planning figures and targets upon which to judge accomplishments.

## 5. Conclusions

This “proof of concept” activity was successful in providing evidence that tens of thousands of NVC and IVC in Kindu and Kibombo could be enumerated, geolocated, and vaccinated with targeted geo-referenced microplanning [39,43,55,59]. Using technology for supervision, for tracking of vaccination teams, and for the identification and precise geo-localization of inhabited localities, combined with the enumeration of children and their vaccination status, enhanced the success of the microplanning strategies outlined in existing vaccination manuals and guidelines [36,37,38]. Documenting the existence of localities within the precise borders of a HZ and its HAs ensured their inclusion in geo-referenced microplans. Unknown and undocumented localities are unlikely to be included in health activity planning. The tracking of vaccination teams using smartphones documented that reaching remote and riverine localities was possible. Kindu and Kibombo HZs can continue to use the data collected during this intensive activity to ensure that all children are successfully reached for health services. The DRC’s CACs and their RECOs are a powerful social mobilization workforce who could be continually engaged to educate populations about the benefits of vaccination; their involvement will always be critical even as technological advances become associated with vaccination delivery [43,55,59,60]. Technical capacity, with the potential for perennial utility, was built among HA and HZ staff who were trained in participatory mapping and in the collection and use of spatially oriented data to plan and successfully implement vaccination for targeted children [36,39,40,41,42,43,44,45].

After an extensive literature search, to our knowledge, this is a unique publication documenting such an activity and including field tools. The successful implementation of the technological innovations presented here was only possible because of the collaboration and engagement of thousands of Congolese community members, HA-, HZ-, antenne- and provincial-level staff, consultant supervisors, and cartography experts working at the most local level, the locality [39,43,55,60]. This engagement led to knowledge transfer about the importance of vaccination and vaccination schedules that cascaded from provincial-level health staff to children’s caretakers. The presence of all necessary logistics for the three PIRIs was due to the strong collaborative nature of all levels of the DRC’s EPI program. Field tools and concepts developed through this work were adapted for use during 2024/25 “Big Catch Up” activities in other Maniema HZs and could be helpful elsewhere [43,46]. In planning for Mashako Plan 3.0, the DRC’s EPI staff might consider streamlining methodologies used in this “proof of concept” and integrating resources available from multiple health programs to reach geographic areas with the greatest need and support adoption on a wider scale [55]. It is important for immunity gaps to be closed in the DRC to prevent the devastating outbreaks of VPDs that have caused tremendous preventable morbidity and mortality over decades [22,23,24,25,26,27,28,29,30,31,32].

## Data Availability

Data supporting the results presented in this manuscript are not immediately accessible due to the possibility of indirect identification of individuals who participated. Certain data will be made available upon request to the corresponding author.

## Supplementary Materials

The following supporting information can be downloaded at https://www.mdpi.com/article/10.3390/vaccines14020175/s1: The Supplemental Document contains the following tables and figures: Supplemental Table S1: Democratic Republic of the Congo, Expanded Programme on Immunization, routine vaccination schedule for children aged 0–23 months; Supplemental Table S2: Democratic Republic of the Congo, Proportion of children aged 0–23 months completely vaccinated with first-year-of-life vaccines or having received no first-year-of-life vaccines, nationally or for Maniema Province, and for Kindu and Kibombo Health Zones, by survey, 1995–2022; Supplementary Table S3A: Kindu Health Zone, Maniema Province, Democratic Republic of the Congo: By health area, results of enumeration of children aged 0–23 months with vaccination status and participatory mapping in 2022 with subsequent extrapolation to age 0–59 months in 2023; Supplementary Table S3B: Kibombo Health Zone, Maniema Province, Democratic Republic of the Congo: By health area, results of enumeration of children aged 0–23 months with vaccination status and participatory mapping in 2022 with subsequent extrapolation to age 0–59 months in 2023; Supplemental Figure S1A: Kindu Health Zone (HZ), Maniema Province, Democratic Republic of the Congo: By HZ and health area, the reasons cited by caretakers for why children 0–23 months of age were never vaccinated in a hospital or health center (July–October 2022); Supplemental Figure S1B: Kindu Health Zone (HZ), Maniema Province, Democratic Republic of the Congo: By HZ and health area, the reasons cited by caretakers for why children 0–23 months of age were incompletely vaccinated despite age eligibility (July–October 2022); Supplemental Figure S2A: Kibombo Health Zone (HZ), Maniema Province, Democratic Republic of the Congo: By HZ and health area, the reasons cited by caretakers for why children 0–23 months of age were never vaccinated in a hospital or health center (July–October 2022); Supplemental Figure S2B: Kibombo Health Zone (HZ), Maniema Province, Democratic Republic of the Congo: By HZ and health area, the reasons cited by caretakers for why children 0–23 months of age were incompletely vaccinated despite age eligibility (July–October 2022); Supplemental Table S4: Kindu and Kibombo Health Zones, Maniema Province, Democratic Republic of the Congo: Participating health area staff and community members during three rounds of Periodic Intensification of Routine Immunization, by health zone, September 2023–January 2024; Supplemental Table S5A: Kindu Health Zone, Maniema Province, Democratic Republic of the Congo: By round of Periodic Intensification of Routine Immunization and data entry method where appropriate, the dates of implementation, number of children aged 0–59 months targeted and participating, the localities represented by participants, and the number of administered doses of oral polio birth dose vaccine and pentavalent vaccines 1,2, and 3; Supplemental Table S5B: Kibombo Health Zone, Maniema Province, Democratic Republic of the Congo: By round of Periodic Intensification of Routine Immunization and data entry method where appropriate, the dates of implementation, number of children aged 0–59 months targeted and participating, the localities represented by participants, and the number of administered doses of oral polio birth dose vaccine and pentavalent vaccines 1,2, and 3. Supplemental Table S6: Kindu and Kibombo Health Zones, Maniema Province, Democratic Republic of the Congo: By health area, the proportion of enumerated children 0-23 months of age without a vaccination card, 2022. The Tools folder contains the following Tools and Figures: Tool S1A: Enumeration questionnaire for children aged 0–23 months for vaccination status in French; Tool S1B: Enumeration questionnaire for children aged 0–23 months for vaccination status in Swahili; Tool S1C: Enumeration questionnaire for children aged 0–23 months for vaccination status in English; Tool S2A: Age guide for children aged 0–23 months in French; Tool S2B: Age guide for children aged 0–23 months in English; Tool S3A: Graphic of the DRC’s routine immunization schedule as of July 2022 in French; Tool S3B: Graphic of the DRC’s routine immunization schedule as of July 2022 in English; Tool S4A: Guide for completing the enumeration questionnaire for children aged 0–23 months for vaccination status in French; Tool S4B: Guide for completing the enumeration questionnaire for children aged 0–23 months for vaccination status in English; Tool S5: Enumerated population aged 0–23 months of age and extrapolated population aged 0–59 months with vaccination status by locality, Kindu HZ; Tool S6A: DRC-specific guide for vaccine eligibility for children aged 0–59 months participating in PIRIs, in French; Tool S6B: DRC-specific guide for vaccine eligibility for children aged 0–59 months participating in PIRIs, in English; Tool S7A: Age guide for children aged 0–59 months in French, example used during PIRI 1, September 2023′ Tool S7B: Age guide for children aged 0–59 months in English, example used during PIRI 1, September 2023; Tool S8A: Coupon provided to children prior to each PIRI to indicate vaccination status in French; Tool S8B: Coupon provided to children prior to each PIRI to indicate vaccination status in English; Tool S9A: Training materials for participants in PIRI activities in French; Tool S9B: Training materials for participants in PIRI activities in English; Tool S10A: Tally sheet to record vaccine doses provided by age category during PIRIs in French; Tool S10B: Tally sheet to record vaccine doses provided by age category during PIRIs in English; Tool S11A: Coupon provided to children aged 24–59 months to record vaccinations administered during PIRIs in French; Tool S11B: Coupon provided to children aged 24–59 months to record vaccinations administered during PIRIs in English; Tool S12A: Tally sheet to record the children’s provenance by locality during each PIRI in French; Tool S12B: Tally sheet to record the children’s provenance by locality during each PIRI in English; Tool S13A: Graphic with suggestions for PIRI vaccination site organization in French; Tool S13B: Graphic with suggestions for PIRI vaccination site organization in English; Tool S14A: Health Area daily PIRI summary in French; Tool S14B: Health Area daily PIRI summary in English; Tool S15A: Health Zone daily PIRI summary in French; Tool S15B: Health Zone daily PIRI summary in English; Tool S16A: PIRI supervision checklist in French; Tool S16B: PIRI supervision checklist in English; Tool S17: Basoko, Kindu “combination map”, by locality, of proportion of never-vaccinated children and the principal reason for never vaccination; Tool S18: Lwama, Kindu “combination map”, by locality, of proportion of never-vaccinated children and the principal reason for never vaccination; Tool S19: Lowe, Kibombo “combination map”, by locality, of proportion of never-vaccinated children and the principal reason for never vaccination; Tool S20: Kasuku 1, Kindu “combination map”, by locality, of proportion of never-vaccinated children and the principal reason for never vaccination; Tool S21: Brazza, Kindu “combination map”, by locality, of proportion of never-vaccinated children and the principal reason for never vaccination; Tool S22: Likeri Reference, Kibombo “combination map”, by locality, of proportion of never-vaccinated children and the principal reason for never vaccination. Document of Ref. [3] provided in Supplementary File S1.

## Abbreviations

The following abbreviations are used in this manuscript:

BCG	Bacille Calmette–Guerin vaccine
BCZS	Bureau Central de la Zone de Santé (Headquarters for the Health Zones)
CAC	Cellules d’Animation Communautaire (Community Animation Cells, Community-based Organization)
CD	Chef de Division of the Division Provinciale de la Santé (Division Chief of the Provincial Health Division
CDC-Atlanta	Centers for Disease Control and Prevention-Atlanta, USA
CVC	Completely Vaccinated Children
DHS	Demographic and Health Survey
DPS	Division Provinciale de la Santé (Provincial Health Division)
DRC	Democratic Republic of the Congo
DTP	Diphtheria, Pertussis, and Tetanus combined vaccine
EPI	Expanded Programme on Immunization
GRID3-M4H	GRID3 Mapping for Health
GTS	Geospatial Tracking System
HA	Health Area
HZ	Health Zone
IPV	Inactivated Polio Vaccine
IT	Infirmier Titulaire (Registered Nurse at the Health Area Level, Nurse)
ITA	Infirmier Titulaire Adjoint (Assistant Registered Nurse at the Health Area Level, Nurse)
IVC	Incompletely Vaccinated Children
KSPH	Kinshasa School of Public Health
MCA	Medicin Chef de l’Antenne (Chief Medical Officer of the Antenne):
MICS	Multiple Indicator Cluster Survey
MCV	Measles-Containing Vaccine
MCZ	Medicin Chef de la Zone (Chief Medical Officer of the Health Zone):
NVC	Never-Vaccinated Children
ODK	Open Data Kit
OPV	Oral Polio Vaccine, Types 1 and 3 Containing
PCV	Pneumococcal 13-Valent Conjugate Vaccine
PENTA	Diphtheria, Tetanus, Pertussis, Hepatitis B, and Haemophilus influenzae type B-Containing Vaccine
PENTA1	First Dose PENTA Vaccine
PENTA2	Second Dose PENTA Vaccine
PENTA3	Third Dose PENTA Vaccine
PIRI	Periodic Intensification of Routine Immunization
RECO	Relais Communautaires (Front Line Community Volunteers, Members of the CAC)
RI	Routine Immunization
ROTA	Rotavirus Vaccine
SIA	Supplementary Immunization Activity
SF	Supplemental Figure
ST	Supplemental Table
VAA	Vaccin Anti-Amaril (Yellow Fever Vaccine)
VAR	Vaccin Anti-Rougeoleux (Measles-Containing Vaccine)
YF	Yellow Fever Vaccine
