# Supplementary material for: Reaching Never- and Incompletely-Vaccinated Children with Routine Immunization: A Proof-of-Concept Activity Using Geo-Referenced Microplans in Two Health Zones in Maniema Province, Democratic Republic of the Congo
Source: Vaccines (Basel). 2026 Feb 13;14(2):175. doi: 10.3390/vaccines14020175 (PMC12945245; doi:10.3390/vaccines14020175)
Supplement: Supplementary file 1 [file vaccines-14-00175-s001.zip › Supplemental_Document_Alleman_Maniema_8Feb2026.pdf]

Supplemental Document for:

Alleman MM, Tanon AA, Rukengwa E, et al. Reaching never- and incompletely-vaccinated children with routine immunization: a proof-of-concept activity using geo-referenced microplans in two health zones in Maniema Province, Democratic Republic of the Congo

Contents

Beyond what is presented below, additional analyses of the data collected as part of the enumeration and vaccination during the rounds of Periodic Intensification of Routine Immunization (PIRIs) were conducted and are available upon request.

Supplemental Table S1: Democratic Republic of the Congo, Expanded Programme on Immunization routine vaccination schedule for children 0-23 months of age

Supplemental Table S2: Democratic Republic of the Congo, Proportion of children 12-23 months of age completely vaccinated with first year of life vaccines or having received no first year of life vaccines, nationally, for Maniema Province, and for Kindu and Kibomo health zones, by survey, 1995-2022

Supplemental Table S3A: Kindu Health Zone, Maniema Province, Democratic Republic of the Congo: By health area, results of enumeration of children 0-23 months of age with vaccination status and participatory mapping in 2022 with subsequent extrapolation to 0-59 months of age in 2023

Supplemental Table S3B: Kibombo Health Zone, Maniema Province, Democratic Republic of the Congo: By health area, results of enumeration of children 0-23 months of age with vaccination status and participatory mapping in 2022 with subsequent extrapolation to 0-59 months of age in 2023

Supplemental Figure S1A: Kindu Health Zone (HZ), Maniema Province, Democratic Republic of the Congo: By HZ and health area, the reasons cited by caretakers for why children 0-23 months of age were never vaccinated in a hospital or health center (July-October 2022)

Supplemental Figure S1B: Kindu Health Zone (HZ), Maniema Province, Democratic Republic of the Congo: By HZ and health area, the reasons cited by caretakers for why children 0-23 months of age were incompletely vaccinated despite age eligibility (July-October 2022)

Supplemental Figure S2A: Kibombo Health Zone (HZ), Maniema Province, Democratic Republic of the Congo: By HZ and health area, the reasons cited by caretakers for why children 0-23 months of age were never vaccinated in a hospital or health center (July-October 2022)

Supplemental Figure S2B: Kibombo Health Zone (HZ), Maniema Province, Democratic Republic of the Congo: By HZ and health area, the reasons cited by caretakers for why children 0-23 months of age were incompletely vaccinated despite age eligibility (July-October 2022)

Supplemental Table S4: Kindu and Kibombo Health Zones, Maniema Province, Democratic Republic of the Congo: Participating health area staff and community members during three rounds of Periodic Intensification of Routine Immunization, by health zone, September 2023 - January 2024

Supplemental Table S5A: Kindu Health Zone, Maniema Province, Democratic Republic of the Congo: By round of Periodic Intensification of Routine Immunization and data entry method where appropriate, the dates of implementation, number of children 0-59 months of age targeted and participating, the localities represented by participants, and the number of administered doses of oral polio birth dose vaccine and pentavalent vaccines 1,2, and 3

Supplemental Table S5B: Kibombo Health Zone, Maniema Province, Democratic Republic of the Congo: By round of Periodic Intensification of Routine Immunization and data entry method where appropriate, the dates of implementation, number of children 0-59 months of age targeted and participating, the localities represented by participants, and the number of administered doses of oral polio birth dose vaccine and pentavalent vaccines 1,2, and 3

Supplemental Table S6: Kindu and Kibombo Health Zones, Maniema Province, Democratic Republic of the Congo: By health area, the proportion of enumerated children 0-23 months of age without a vaccination card, 2022

Supplemental Table 1: Democratic Republic of the Congo, Expanded Programme on Immunization, routine vaccination schedule for children 0-23 months of age [reference 4 in article reference list]

Vaccine	Details	Schedule
OPV	Oral polio vaccine, types 1 and 3 containing	At birth, 6, 10, 14 weeks
BCG	Bacille Calmette-Guerin	At birth
PENTA	Diphtheria, Tetanus, Pertussis, Hepatitis B, and *Haemophilus influenzae* type B (Pentavalent vaccine)	6, 10, 14 weeks
PCV-13	Pneumococcal 13-valent conjugate	6, 10, 14 weeks
ROTA	Rotavirus	6, 10, 14 weeks
IPV	Inactivated polio vaccine	14 weeks, 9 months
MEASLES	Measles containing	9, 15 months
YELOW FEVER	Yellow fever	9 months

Supplemental Table 2: Democratic Republic of the Congo, Proportion of children 12-23 months of age completely vaccinated with first-year-of-life vaccines or having received no first-year-of-life vaccines, nationally, for Maniema Province, and for Kindu and Kibomo health zones, by survey, 1995-2022

Source [reference in article reference list]	Vaccination in children 12-23 months of age							
	National		Maniema Province		Kindu Health Zone		Kibombo Health Zone	
	Completely-vaccinated* %	Received no first-year-of-life vaccines† %	Completely-vaccinated* %	Received no first-year-of-life vaccines† %	Completely-vaccinated* %	Received no first-year-of-life vaccines† %	Completely-vaccinated* %	Received no first-year-of-life vaccines† %
MICS 1995 [12]	6							
MICS 2001 [13]	23	19	3	30				
DHS 2007 [9]	31	18	10	31				
MICS 2010 [14]	42	10	10	24				
DHS 2013-14 [10]	45	6	42	11				
MICS 2017-18 [15]	35	20	7	59				
KSPH 2020 [16] ‡	53	9	29	28	35		14	
KSPH 2021 [17]	42	13	15	44	28	20	4	80
KSPH 2022 [18]	45	13	13	41	21	36	7	66

Abbreviations and shading:

Grey shading: not available because data were not collected, analysis was not presented in the report, or survey was not designed for analysis at this geographic level

MICS: Multiple Indicator Cluster Survey;

DHS: Demographic and Health Survey;

KSPH: Kinshasa School of Public Health

* Completely-vaccinated children: Children 12-23 months of age who had received all first-year-of-life vaccines included in the Democratic Republic of the Congo's Expanded Programme on Immunization schedule in the year being surveyed. In some survey years, the birth dose of oral polio vaccine is not included in the indicator. Details can be found in the survey documents.

† Received no first-year-of-life vaccines: Children 12-23 months of age who had received no first-year-of-life vaccines included in the Democratic Republic of the Congo's Expanded Programme on Immunization schedule in the year being surveyed.

‡ The 2020 KSPH vaccination coverage survey included 18 of DRC's 26 provinces

Supplemental Table 3A: Kindu Health Zone, Maniema Province, Democratic Republic of the Congo: By health area, results of enumeration of children 0-23 months of age with vaccination status and participatory mapping in 2022 with subsequent extrapolation to 0-59 months of age in 2023														
Health Areas of Kindu Health Zone	No. of localities identified during participatory mapping	Total children No.		Never vaccinated in hospital/health center					Incompletely vaccinated according to age			Completely vaccinated according to age		
		0-23 months of age	0-59 months of age (extrapolated from enumeration)	0-23 months of age No., %		% localities with >=50% children 0-23 months never vaccinated	% localities with >=75% children 0-23 months never vaccinated	0-59 months of age (extrapolated from enumeration) No.	0-23 months of age No., %		0-59 months of age (extrapolated from enumeration) No.	0-23 months of age No., %		0-59 months of age (extrapolated from enumeration) No.
Basoko	54	4,392	10,980	1,646	37	22	6	4,115	230	5	575	2,516	57	6,290
Brazza	40	1,751	4,378	1,068	61	80	3	2,670	28	2	70	655	37	1,638
Kasuku 1	48	3,501	8,753	825	24	6	0	2,063	144	4	360	2,532	72	6,330
Kasuku 2	35	1,230	3,075	353	29	11	0	883	115	9	288	762	62	1,905
Libenga	35	993	2,483	153	15	9	0	383	27	3	68	811	82	2,028
Lumbulumbu	22	2,286	5,715	948	41	27	0	2,370	123	5	308	1,215	53	3,038
Lwama	39	6,170	15,425	2,497	40	62	46	6,243	419	7	1,048	3,254	53	8,135
Mikelenge	46	2,784	6,960	1,139	41	26	9	2,848	263	9	658	1,382	50	3,455
RVA	39	3,020	7,550	908	30	5	0	2,270	278	9	695	1,834	61	4,585
Tokolote	57	3,311	8,278	1,657	50	51	2	4,143	153	5	383	1,501	45	3,753
Trois Z	15	399	998	94	24	0	0	235	65	16	163	240	60	600
Total Kindu	430	29,837	74,593	11,288	38	30	6	28,220	1,845	6	4,613	16,702	56	41,755

Abbreviations:
No., Number

Supplemental Table 3B: Kibombo Health Zone, Maniema Province, Democratic Republic of the Congo: By health area, results of enumeration of children 0-23 months of age with vaccination status and participatory mapping in 2022 with subsequent extrapolation to 0-59 months of age in 2023														
Health Areas of Kibombo Health Zone	No. localities identified during participatory mapping	Total children No.		Never vaccinated in hospital/health center					Incompletely vaccinated according to age			Completely vaccinated according to age		
		0-23 months of age	0-59 months of age (extrapolated from enumeration)	0-23 months of age No., %		% localities with >=50% children 0-23 months never vaccinated	% localities with >=75% children 0-23 months never vaccinated	0-59 months of age (extrapolated from enumeration) No.	0-23 months of age No., %		0-59 months of age (extrapolated from enumeration) No.	0-23 months of age No., %		0-59 months of age (extrapolated from enumeration) No.
Bilundu	4	1,056	2,640	629	60	100	0	1,573	81	8	203	346	33	865
Difuma 2	23	532	1,330	241	45	48	9	603	119	22	298	172	32	430
Kasuku	14	559	1,398	436	78	93	64	1,090	37	7	93	86	15	215
Kaswa	19	719	1,798	380	53	32	16	950	31	4	78	308	43	770
Kiyungi	13	1,513	3,783	647	43	23	0	1,618	361	24	903	505	33	1,263
Likeri Reference	22	867	2,168	300	35	32	5	750	288	33	720	279	32	698
Lokenie	6	638	1,595	181	28	17	0	453	42	7	105	415	65	1,038
Lowe	19	931	2,328	485	52	68	37	1,213	186	20	465	260	28	650
Lweki	18	573	1,433	314	55	83	39	785	120	21	300	139	24	348
Methodiste Kibombo	7	990	2,475	684	69	86	14	1,710	71	7	178	235	24	588
Methodiste Likeri	12	809	2,023	375	46	42	8	938	102	13	255	332	41	830
Nganze	11	395	988	143	36	27	0	358	76	19	190	176	45	440
Total Kibombo	168	9,582	23,955	4,815	50	52	20	12,038	1,514	16	3,785	3,253	34	8,133

Abbreviations:
No., number

Supplemental Figure 1A: Kindu Health Zone (HZ), Maniema Province, Democratic Republic of the Congo: By HZ and health area, reasons cited by caretakers for why children aged 0-23 months were never vaccinated in a hospital or health center (July-October 2022)

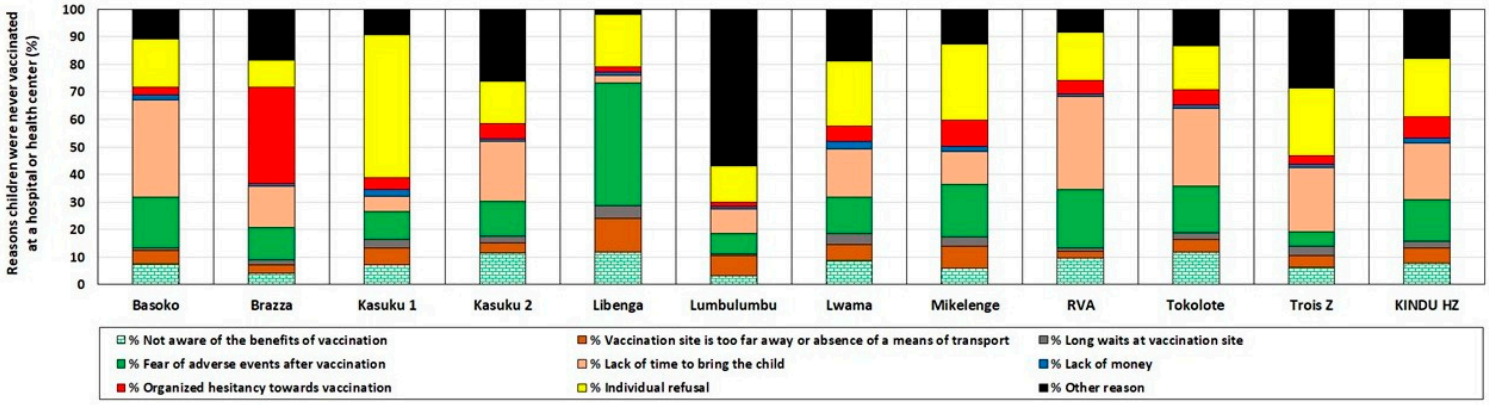

Supplemental Figure 1B: Kindu Health Zone (HZ), Maniema Province, Democratic Republic of the Congo: By HZ and health area, reasons cited by caretakers for why children aged 0-23 months were incompletely vaccinated despite age eligibility (July-October 2022)

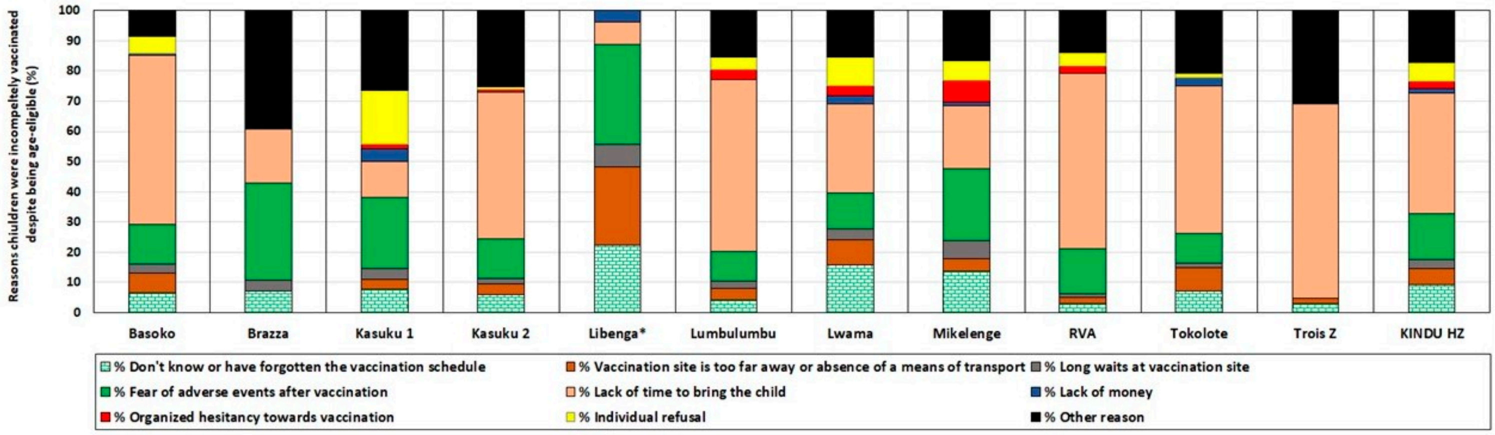

*Incomplete data for two children from Libenga AS; therefore, they were excluded from these analyses.

Supplemental Figure 2A: Kibombo Health Zone (HZ), Maniema Province, Democratic Republic of the Congo: By HZ and health area, reasons cited by caretakers for why children aged 0-23 months were never vaccinated in a hospital or health center (July-October 2022)

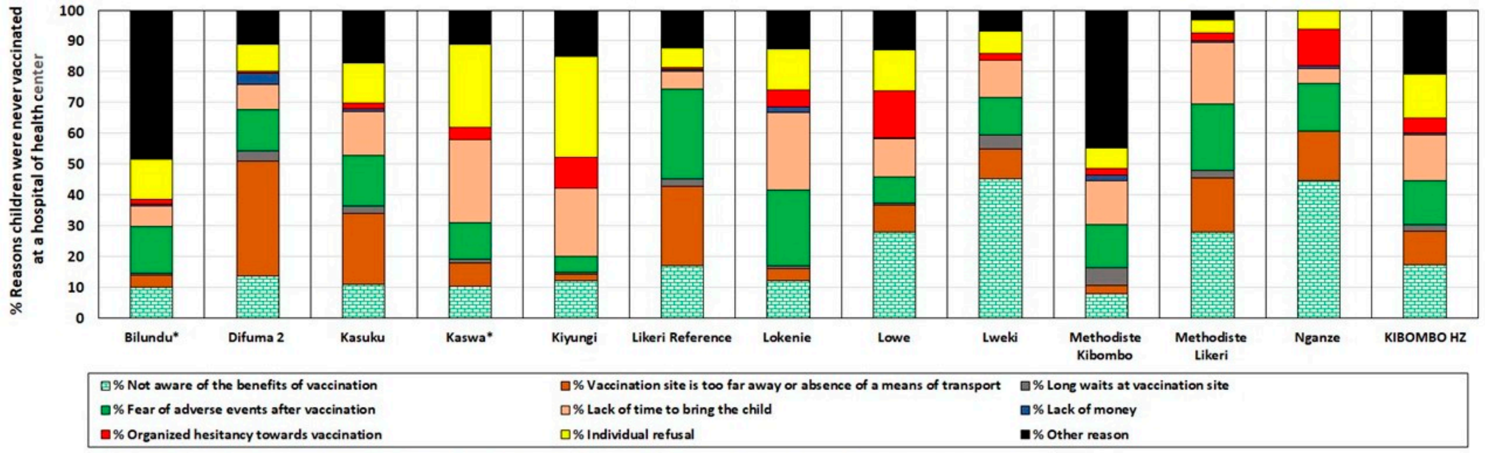

*Data were incomplete for 6 children in Kaswa AS (localité Maniema 1) and for 12 children in Bilundu AS (localité Bilundu); therefore, those children were excluded from these analyses.

Supplemental Figure 2B: Kibombo Health Zone (HZ), Maniema Province, Democratic Republic of the Congo: By HZ and health area, reasons cited by caretakers for why children aged 0-23 months were incompletely vaccinated despite age eligibility (July-October 2022)

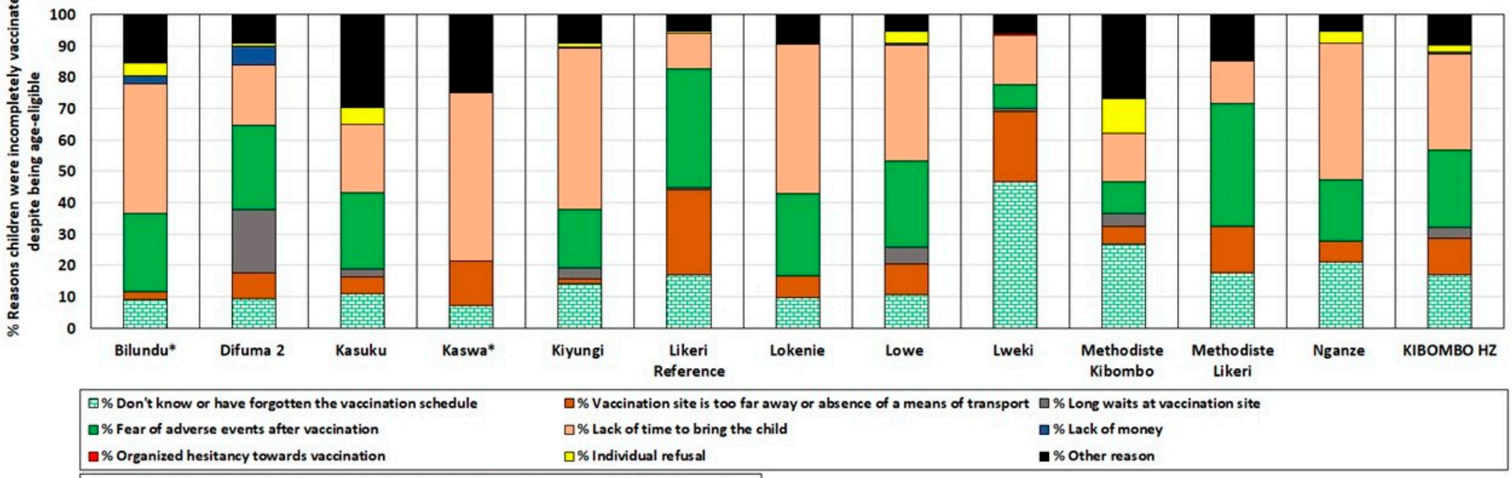

*Incomplete data for 3 children from Kaswa AS and 4 from Bilundu AS; therefore they were excluded from these analyses.

Supplemental Table 4: Kindu and Kibombo Health Zones, Maniema Province, Democratic Republic of the Congo: Participating health area staff and community members during three rounds of Periodic Intensification of Routine Immunization, by health zone, September 2023 - January 2024

	PIRI 1 September 2023		PIRI 2 November 2023		PIRI 3 January 2024	
	Kindu	Kibombo	Kindu	Kibombo	Kindu	Kibombo
No. of vaccination teams	137	76	134	76	134	76
No. of members/team*	5	5	3	3	3	3
No. of RECO	1,038	425	1,038	425	1,038	425
No. of town criers	176	168	134	168	134	168
No. of CAC leaders**			11	12	11	12

Abbreviations:
PIRI, Periodic Intensification of Routine Immunization;
No., Number
RECO, Relais communautaires who are members of the CAC;
CAC, Cellules d'Animation Communautaire
*The first PIRI was integrated with a measles follow-up campaign targeting children 6-59 months; due to additional vaccination and documentation required at vaccination sites, teams needed 5 persons.

**For the first PIRI, the participation of CAC leaders was not recorded separately from others who participated.

Supplemental Table 5A: Kindu Health Zone, Maniema Province, Democratic Republic of the Congo: By round of Periodic Intensification of Routine Immunization and data entry method where appropriate, the dates of implementation, number of children 0-59 months of age targeted and participating, the localities represented by participants, and the number of administered doses of oral polio birth dose vaccine and pentavalent vaccines 1,2, and 3

PIRI	Date	Estimated target (No.) 0-59 months never vaccinated + incompletely vaccinated	No. children participating in the PIRI that were registered on the provenance form at a vaccination site		No. of localities identified during mapping	No. (%) of localities represented by children participating in the PIRI as registered on the provenance form at a vaccination site	No. doses oral polio vaccine birth dose (OPV0)		No. doses first dose Pentavalent vaccine (PENTA1)		No. doses second dose Pentavalent vaccine (PENTA2)		No.doses third dose Pentavalent vaccine (PENTA3)		Total No. doses VPO0, PENTA1,2,3	
			Smart phone entry	EXCEL entry		Smart phone entry	Smart phone entry	EXCEL entry	Smart phone entry	EXCEL entry	Smart phone entry	EXCEL entry	Smart phone entry	EXCEL entry	Smart phone entry	EXCEL entry
1	27 September - 1 October 2023	32,833	18,030	Not recorded	430	370 (86.0%)	1,522	1,520	8,600	9,475	3,343	3,413	2,685	2,990	16,150	17,398
2	29 November - 3 December 2023	32,833	20,598	32,291	430	396 (92.1%)	1,120	803	5,789	6,279	5,763	6,273	2,765	4,303	15,437	17,658
3	13 - 17 January 2024	32,833	22,967	27,337	430	397 (92.3%)	910	1,410	4,243	7,506	4,728	7,349	7,623	10,288	17,504	26,553

Abbreviations:
PIRI, Periodic Intensificaton of Routine Immunizaiton
No., number

Supplemental Table 5B: Kibombo Health Zone, Maniema Province, Democratic Republic of the Congo: By round of Periodic Intensification of Routine Immunization and data entry method where appropriate, the dates of implementation, number of children 0-59 months of age targeted and participating, the localities represented by participants, and the number of administered doses of oral polio birth dose vaccine and pentavalent vaccines 1,2, and 3

PIRI	Date	Estimated target 0-59 months never vaccinated + incompletely vaccinated	No. children participating in the PIRI that were registered on the provenance form at a vaccination site		No. of localities identifiied during mapping	No. (%) of localities represented by children participating in the PIRI as registered on the provenance form at a vaccination site	Doses oral polio vaccine birth dose (OPV0)		Doses first dose Pentavalent vaccine (PENTA1)		Doses second dose Pentavalent vaccine (PENTA2)		Doses third dose Pentavalent vaccine (PENTA3)		Total doses VPO0, PENTA 1,2,3	
			Smart phone entry	EXCEL entry		Smart phone entry	Smart phone entry	EXCEL entry	Smart phone entry	EXCEL entry	Smart phone entry	EXCEL entry	Smart phone entry	EXCEL entry	Smart phone entry	EXCEL entry
1	29 September - 3 October 2023	15,823	9,454	Not recorded	168	130 (77.4%)	741	475	5,825	4,959	2,692	2,882	1,991	2,052	11,249	10,368
2	28 November - 2 December 2023	15,823	13,317	16,286	168	154 (91.7%)	525	517	3,839	3,331	6,072	6,766	2,074	2,261	12,510	12,875
3	14 - 18 January 2024	15,823	13,733	16,803	168	154 (91.7%)	189	243	1,207	1,136	2,697	2,802	11,298	10,702	15,391	14,883

Abbreviations:
PIRI, Periodic Intensificaton of Routine Immunizaiton
No., number

Supplemental Table 6: Kindu and Kibombo Health Zones, Maniema Province, Democratic Republic of the Congo: By health area, the proportion of enumerated children 0-23 months of age without a vaccination card, 2022

Health Areas of Kindu Health Zone	% children without a vaccination card		Health Areas of Kibombo Health Zone	% children without a vaccination card
Basoko	53		Bilundu	67
Brazza	62		Difuma 2	61
Kasuku 1	27		Kasuku	85
Kasuku 2	43		Kaswa	56
Libenga	26		Kiyungi	62
Lumbulumbu	45		Likeri	58
Lwama	47		Lokenie	47
Mikelenge	54		Lowe	68
RVA	40		Lweki	84
Tokolote	58		Metho Kibb	75
Trois Z	31		Metho Lkr	50
Total Kindu	46		Nganze	70
			TOTAL KIBOMBO	65
