# Supplementary material for: Reaching Never- and Incompletely-Vaccinated Children with Routine Immunization: A Proof-of-Concept Activity Using Geo-Referenced Microplans in Two Health Zones in Maniema Province, Democratic Republic of the Congo
Source: Vaccines (Basel). 2026 Feb 13;14(2):175. doi: 10.3390/vaccines14020175 (PMC12945245; doi:10.3390/vaccines14020175)
Supplement: Supplementary file 1 [file vaccines-14-00175-s001.zip › Supplementary File S1.pdf]

COUNTRY ASSESSMENT
COMBATTING CHILDHOOD COMMUNICABLE DISEASES PROJECT
ZAIRE

Country Assessment Team:

Government of Zaire
Centers for Disease Control
USAID Zaire
WHO ZAIRE
UNICEF ZAIRE

July 1982

INDEX

Title	Page
1. CCCD in Africa	5
1.1 Scope of Project	5-6
1.2 Purpose of Project	6
1.3 Country Specific Activity Project Design	6
2. Country Assessment for Zaire	6-7
2.1 Method of Assessment	7
2.2 Geographic and Demographic Data	7
2.2.1 Geographic Data	8
2.2.2 Demographic Data	9
2.3 Childhood Infectious Disease Epidemiology	9
2.4 Official Government Health Policies And Plans	9
2.4.1 National Health Plan	9-10
A. Background	9
B. Strategy Proposed by National Health Plan	9
C. Objectives and Targets of National Health Plan	9-10
D. Feasibility	10-11
(1) Government Commitment	11-12
(2) (a) Resources Required for Implementation	12
(b) Resources Required for Maintenance (Recurrent Costs)	12-14
(3) Resources Currently Available/Committed	14
(4) Summary of Feasibility	14
2.4.2 Expanded Program on Immunization	14
A. Background	14-15
B. Current Achievements	16
C. Objectives and Targets of Zaire EPI	16-18
D. Feasibility	18
(1) Government Commitment	18
(2) Resources Required	19
(3) Resources Currently Available/Committed	19-21
(4) Summary of Feasibility	22
2.4.3 Control of Diarrheal Diseases	22
A. Background	22
B. Current Achievements	22

	C. Objectives and Targets of Zaire CDD	22-23
	D. Feasibility	24
	(1) Government Commitment	24
	(2) Resources Required	24-25
	(3) Resources Currently Available/Committed	26
	(4) Summary of Feasibility	26
2.4.4	Malaria Control	26
	A. Background	26-27
	B. Current Achievements	27
	C. Objectives and Targets of Zaire Malaria Control	27-28
	D. Feasibility	28
	(1) Government Commitment	28-29
	(2) Resources Required	29
	(3) Resources Currently Available/Committed	29
	(4) Summary of Feasibility	30
3.	Proposals for CCCD in Zaire	30
3.1	Proposed Scope of CCCD in Zaire	30-31
3.2	Proposed CCCD Regional Support Project Assistance	31-33
3.3	Proposed CCCD Bilateral Assistance	33-35

Tables

Title		Page
Table 1	Hospital Admission and Death Information, Zaire	8
Table 2	Projected EPI Needs According to EPI National Plan of Action, 1982 - 1986	20
Table 3	Anticipated Commodity Support for EPI Zaire, 1982-1986 (Donors other than USAID)	21
Table 4	Estimated Number of ORS Packets Required for CDD Zaire	25
Table 5	Number of Health Zones (Urban plus Rural) to be Operational in Zaire, 1982-1986	25
Table 6	Proposed CCCD Bilateral Commodity Support, 1982-1986	36

Annexes

Annex 1	List of Persons and Documents Consulted	37
Annex 2	List of Technical Officer's Tasks	38
Annex 3	Projected Expansion of PHC Activities in Zaire	39
Annex 4	Projected Population Growth in Zaire	40

1. CCCD IN AFRICA

1.1 SCOPE OF PROJECT

The Combatting Childhood Communicable Disease Project (CCCD) is a major cooperative effort of African nations, WHO/AFRO and other nations to reduce childhood mortality, disability and morbidity in Africa through improved prevention and control of childhood infectious diseases. CCCD is a specific response of CDA (Cooperation for Development in Africa) to the World Health Organization's request for increased technical cooperation in support of primary health care programs in Africa.

Assessments of current health status in Africa clearly identify children under 5 at exceptional risk of morbidity and mortality. Of the four million children born each year in Africa, it is estimated that 25% die before their fifth birthday. Millions more are debilitated or permanently disabled by disease.

This high risk of death and disability is 20-25 times the risk of children born in developed countries and represents an unacceptable level of human suffering. The basic causes reflect the complex interaction of undernutrition, infectious disease and economic underdevelopment. The priority and importance of economic development to the long-term improvement in health cannot be overlooked. A firm government commitment to integrated primary health care, with encouragement of community-based initiatives to prevent and control infectious diseases, can significantly improve child health during the next ten years. Integrated primary health care with preventive and curative services and community-based initiatives is the basic philosophy of the CCCD project.

1.2 Purpose of Project

CCCD seeks to provide a regional mechanism for strengthening the ability of African health institutions, whether national or regional, to identify priority childhood infectious diseases. It also seeks to improve their ability to design, implement and evaluate appropriate integrated strategies for prevention and control of these diseases.

Three categories of childhood disease, for which effective control technology exists, have been assigned priority in the CCCD project. These are the vaccine preventable diseases, acute diarrheal disease and malaria. These priorities are based on estimates of under 5 motality from the major infectious diseases in Africa. The project is not limited to these three elements. It may be expanded to include other diseases which may prove to be important causes of childhood morbidity and mortality in certain nations, such as the hemorrhagic fevers, meningitis, yellow fever or yaws.

The CCCD project makes available two types of technical cooperation; these are regional and bilateral (country-specific). Any African nation choosing to participate in CCCD can request one or more of the following regional elements:

- Training
- Training Development/Adaptation
- Health Education/Promotion
- Operations Research
- CCCD Health Information System

In addition, certain countries will receive bilateral (country-specific) assistance. The U.S. currently plans to provide bilateral assistance to 12 countries during the first seven years of the project. It is expected that other CDA countries will provide bilateral assistance during the same time period. U.S. bilateral assistance is projected for 48 months in each country, to begin with 2 countries in 1982. Any country receiving bilateral assistance must comply with the USAID 25% host country contribution rules.

The regional activities are totally donor funded, have no recurrent cost implications and are not subject to the 25% host country contribution rules. An African country may receive assistance from the regional component, the bilateral component, or both.

1.3 Country-Specific Activity Project Design

A collaborative assessment is a prerequisite for selection of a country to receive country-specific activity support. The objective of the assessments is to determine the current health situation in candidate countries, to assess their current plans and strategies, available and required resources, and to specify and recommend, as appropriate, specific external assistance needs. The final country assessment report should contain the information necessary to enable development of a project agreement if a country is selected for CCCD bilateral assistance.

2. Country Assessment for Zaire

2.1 Method of Assessment

In February 1982, a country assessment was conducted by a team consisting of representatives of the Government of Zaire, the Centers for Disease Control/Atlanta, USAID Zaire, WHO Zaire, and UNICEF Zaire. It coordinated its assessment with the advisory committee of the Zaire Expanded Program on Immunization (EPI). The team reviewed geographic and demographic data, childhood infectious disease epidemiology and the National Health Plan. It studied existing and projected government of Zaire plans for the Expanded Program on Immunization, Control of Diarrheal Diseases and Malaria Control. The feasibility of implementing these plans within a Primary Health Care framework was assessed, and proposals for CCCD cooperation were developed.

2.2 Geographic and Demographic Data

2.2.1 Geographic Data

The Republic of Zaire is one of the largest countries of Africa, having an area of 2,346,200 km^2. Zaire occupies the geographic center of the African continent, being located between 3° 20" latitude north and 13° 20" latitude south. The climate is tropical, and most of the country is covered by equatorial rain forest.

2.2.2 Demographic Data

The 1982 estimated population of Zaire is 28,120,000, giving an average population density of 12 persons per km^2. Thirty percent of the population is urban (living in communities of greater than 10,000 population). The remaining 70% of the population is classified as rural.

The age distribution of the population is estimated as follows (1975 census):

0-11 months	4.23% or	1,900,000
12-23 months	3.75% or	1,055,000
2-5 years	11.02% or	3,100,000
0-15 years	46.00% or	12,935,000

The following estimates relating to health and potential CCCD cooperation are obtained from the National Health Plan:

Crude Birth Rate	47/1000
Crude Death Rate	18/1000
Infant Mortality Rate	172/1000 live births
Under-five mortality rate (cumulative over 5 years)	350/1000 live births
Life expectancy at birth	46 years
Literacy rate	15%
Per capita GNP	$260 U.S.
Number of radios in use	101/1000 population

The fact that the under-five mortality rate, as reported in the National Health Plan, comprises a very large proportion of the crude death rate attests to the importance of childhood mortality. It also may indicate a need for additional mortality data from which more realistic rates may be estimated.

to the importance of childhood mortality. It also may indicate a need for additional mortality data from which more realistic rates may be estimated.

2.3 Childhood Infectious Disease Epidemiology

Based on data reported from hospitals in 1975, from throughout the country, the National Health Plan of Zaire lists infectious and parasitic diseases as the most important causes of overall morbidity and mortality. Malaria is described as the most important cause of morbidity from infectious disease and the most important cause of infectious disease mortality during the first year of life. Measles is described as the greatest overall cause of infectious disease-related mortality. Cholera and other acute diarrheal diseases are likewise listed as important causes of mortality. Malnutrition and birth defects are listed as important non-infectious causes of under-five mortality. The following supplemental information about measles, diarrheal diseases and malaria in the under-five population was developed by the pre-program assessment team from an analysis of records at Mama Yemo Hospital (Kinshasa), Kisantu Hospital (Kisantu Rural Health Zone) and Ngidinga Maternity Dispensary (Kisantu Rural Health Zone). The information is presented for the years 1976, 1978 and 1980, and clearly confirms that these three diseases are serious childhood health problems in both urban and rural Zaire.

TABLE 1
HOSPITAL ADMISSION AND DEATH INFORMATION, ZAIRE

Average Percentage of Total, 1976, 1978, 1980	Mama Yemo (5 Years)	Kisantu (15 Years)	Ngidinga (15 Years)
% Total Pediatric Admissions due to Measles	3,576/30,716 (12%)	1,554/5,518 (28%)	239/1,837 (13%)
% Total Pediatric Admissions due to Diarrheal Disease	3,885/30,716 (13%)	587/5,518 (11%)	183/1,837 (10%)
% Total Pediatric Admissions due to Malaria/Anemia	4,781/30,716 (16%)	1,267/5,518 (23%)	588/1,837 (32%)
% Total Pediatric Deaths due to Measles	1,452/11,813 (12%)	365/1,034 (35%)	29/197 (15%)
% Total Pediatric Deaths due to Diarrheal Disease	1,046/11,813 (9%)	80/1,034 (8%)	28/197 (14%)
% Total Pediatric Deaths due to Malaria/Anemia	1,623/11,183 (14%)	151/1,034 (15%)	62/197 (32%)

2.4 Official Government Health Policies and Plans

2.4.1. National Health Plan

A. Background

In 1980 the Government of Zaire signed the "Chartre du Develópement Sanitaire en Afrique" which has as its basis the adoption of a primary health care strategy for achieving health for all by the year 2000.

In 1981 the Comite Central du Mouvement Populaire de la Revolution (The President's Advisory Commitee) authorized primary health care (PHC) as the basis for a national strategy to achieve the goal of health for all by the year 2000.

A new National Health Plan covering the five year period 1982-1986 was completed in January 1982. This plan endorses PHC as the means of achieving health for all in Zaire by the year 2000. The plan represents the official health policy of the Government of Zaire.

B. Strategy Proposed by National Health Plan

The new National Health Plan envisions the development of a national primary health care system through the establishment of urban and rural health zones. Health care in these zones will be established as a 4-level referral system. At the first level will be the local public health educators ("animateurs") who will function at the community level to promote understanding and use of the PHC system. As presently envisioned, "animateurs" will not offer treatment. The next level will be the health posts, or dispensaries, which will be the primary level for preventive and curative medical srvices. The next highest level will be the health centers, which will serve as the reference for curative services that cannot be provided at health posts. Health centers may also offer the same basic PHC services as health posts. A hospital will be the final level of reference for curative services in the health zone. The reference hospital will be the headquarters of the health zone.

Health zones will be defined in terms of an appropriate "catchment area" for reference hospitals. As such they will not necessarily correspond to existing administrative boundaries. The National Health Plan envisions that by the end of 1986 there will be 17 urban and 124 rural health zones established and that they will provide access for integrated PHC services to approximately 60% of the total population of Zaire. Between 10 and 15 of these health zones are presently functioning. Most remain to be identified and defined.

C. Objectives and Targets of National Health Plan

The National Health Plan promotes the integration of existing vertical programs of the Department of Public Health, specifically EPI activities, into the national PHC system. The plan also promotes establishment of a

plan for a diarrheal diseases control strategy to be carried out as an integrated activity within the Primary Health Care system. Several aspects of the malaria control strategy presented in the National Health Plan lend themselves to inclusion in the envisioned national PHC system.

Specific goals and objectives for the EPI, CDD and Malaria Control activities listed in the National Health Plan are summarized in the following table:

OBJECTIVES AND TARGETS, NATIONAL HEALTH PLAN (1982 - 1986)

CONTROL PROGRAM	OBJECTIVES	TARGETS
EPI	Reduction of morbidity and mortality due to the six childhood diseases which can be prevented by immunization by assuring a vaccination coverage of 80% for the target population by the year 2000.	1. Provide vaccines to all PHC posts.<br>2. Assure training of PHC personnel responsible for integration of EPI activities<br>3. Assure uninterrupted supply of vaccines and of cold chain and vaccination supplies
CDD	*Guarantee a progressive availability of primary health care to the entire population.	1. Control diarrheal diseases through an appropriate control strategy.
Malaria Control	Reduction of morbidity and mortality due to malaria in all age groups but especially among children under 15, pregnant women and nursing mothers.	1. Prevent malaria morbidity and mortality, especially in urban and peri-urban areas, by vector control.<br>2. Assure protection from malaria morbidity and mortality by means of health education.<br>3. Assure preventive and curative treatment for malaria in the pilot malaria control pilot areas.

*CDD is listed under Primary Health Care.

D. Feasibility

The National Health Plan is a new document, having been completed shortly before the country assessment was carried out. It is the official Government statement of planned health activities in Zaire for the five-year period ending December 31, 1986.

The National Health Plan is ambitious. At the present time, based on accessibility of EPI services, it is estimated that health services are accessible to only 20% of the people of Zaire. The National Health Plan calls for accessibility of primary health care services to be expanded so that by 1986 such services will be accessible to 60% of the population of Zaire. Extrapolation of this rate of progress would allow 100% access by 1990, which would be in keeping with WHO EPI's stated goal of making immunizations available to all the world's children by 1990.

It must be kept in mind that an expansion of services assumes that the services made available will be at or above certain minimum standards. Expansion in name only, in order to keep pace with a stated goal is to be avoided. The stated goal of increasing the availability of quality health services to an additional 10% of the total population each year while transforming the system to a national primary health care system at the same time, represents a monumental undertaking. Achievement of this goal will require a concurrent training of supervisory personnel and development of supervisory and logistics systems necessary to make such expansion possible.

This undertaking will require large amounts of external resources in addition to what the Government of Zaire can make available from its Treasury. The country assessment team feels that if the amount of resources needed for the activities necessary to implement the Plan's objectives can be made available from the Government of Zaire and external resources, the National Health Plan is feasible. If the amount of resources necessary for full implementation of National Health Plan objectives cannot be mobilized, Plan implementation will have to take place at a correspondingly reduced rate, with the ultimate result that implementation accomplishments will fall short of stated Plan objectives.

(1) Government Commitment

The National Health Plan was drafted by Zairois civil servants of the Department of Public Health. It is clear that the individuals who drafted the Plan as well as other dedicated civil servants with the Department are committed to the Plan. As can be seen in Section (3) "Resources Currently Available or Committed," the Government of Zaire has also made a strong financial commitment to those aspects of the Plan for which CCCD support is being proposed.

Important to the success of implementation of the Primary Health Care system envisioned in the National Health Plan is the identification of a focus within the Department of Public Health that will be responsible for coordinating and overseeing its implementation. A new Direction for Primary Health Care has recently been created within the Department of Public Health. The responsibility for coordinating and overseeing the implementation of PHC activities in Zaire will lie with this Direction. Policy decisions and instructions will, of course, come from Ministerial and Secretary General levels of the Department of Public Health, but these will be channeled through the Primary Health Care Direction.

In March 1982, meetings were held at which all Regional Medical Inspectors were instructed to delineate and assess the current health resources of the health zones in their regions. The early identification of the boundaries of all urban and rural health zones will facilitate the identification of specific health zones to be included in each year's expansion. This, in turn, will enable realistic projections of personnel, training and commodity needs.

It is essential that informal coordination mechanisms be established among programs responsible for implementing PHC activities. This is especially true for the USAID supported Basic Rural Health project and proposed CCCD activities. It is encouraging that PHC implementors are already establishing informal means of coordinating their activities. With such informal mechanisms for coordinating PHC implementation activities and with a Government focus for referring coordination problems that cannot be resolved informally, the feasibility of National Health Plan implementation, at least with respect to PHC, is greatly enhanced.

(2)(a) Resources Required for Implementation

Central to the Zaire PHC system concept is the establishment of urban and rural health zones that, in the aggregate, will cover the entire country. Under instructions from the Ministerial and Secretary-General levels of the Department of Public Health, the new Primary Health Care Directorate will be responsible for seeing that health zones are established and PHC activities are implemented in them according to schedule. Resources required to carry out this responsibility include supervisory personnel with field experience and the transportation and fuel necessary to assure their nation-wide mobility.

The PHC system envisioned by the National Health Plan will not require significant increases in personnel. The intention is to take existing health personnel assigned to health facilities and train them so that they and their facilities can be integrated into a PHC system. There will be a need to train supervisory personnel and personnel who will assume logistics responsibilities. While the need for additional personnel will be minimal, training needs will be great.

The expansion of integrated PHC services to an additional 10% of the total Zaire population during each year of the Plan will require significant increases in supplies and equipment.

The financial resources needed to support the envisioned implementation of an integrated PHC system in Zaire are going to require increased financial resources from the Government of Zaire and from external donors.

2(b) Resources Required for Maintenance (Recurrent Costs)

The primary health care expansion proposed in the National Health Plan (1982-86) will require additional resources to maintain expanded services once they are implemented. The major component of facilities and personnel are currently in place and financed by either the government or religious

missions. Some new facilities may have to be built and many will have to be restored and re-equipped, but this will represent one-time expenditures.

Additional recurrent costs will occur in the area of drugs and supplies, more adequate systems of supervision, supply and transport, and extension of activities to a fourth level of service; the village animateurs. At present there is little information as to what will be the additional recurrent costs of the proposed primary health care system once it is implemented. The principal additional cost components are vaccines and supplies which are estimated to total 27 million Z in the fourth year of the project, or about 10% of current total health expenditures. It is clear, however, that a system is planned which will capture a substantial amount of private sector resources through a system of user fees and/or fees for services. Such fees are proposed to be of 3 types.

1) A one time inscription fee of 10 Z.
2) Fees for service of 6Z for each acute or chronic episode treated and
3) A fee of 6Z to purchase an immunization card which would entitle the holder of the card to all vaccinations.

It is estimated that such a fee system will produce 30% of all resources required in rural health zones.

The funds collected are to be used two ways. The first is to finance salaries of personnel not on the governmental roles. The second is to finance the purchase of medication.

While it is difficult to estimate the impact that the system of fees may have on the implementation of the primary health care system as a whole, it is much easier to show the possible impact it may have on recurrent costs on such services as immunizations, oral rehydration therapy and anti-malaria treatment. Reasonable cost assumptions for vaccination recurrent costs would be:

Vaccines	2 Z
Supplies	1
Operating Costs	3
Equipment Transport	3
Buildings	1
Personnel	9
Total	19 Z

The 6Z Fee will thus meet about 1/3 the total recurrent costs. Keeping in mind that the government already funds personnel and buildings and the majority of operating costs, it becomes clear that the 6Z fee could cover the remaining components of vaccines, supplies and equipment and transport.

For diarrheal disease oral rehydation treatment and anti-malarial treatment the 6Z fee will about equal all estimated costs for the treatment provided, including personnel salaries. These costs are estimated to be:

Medication		0.5Z
Supplies		-
Operating costs		1.5
Equipment and Transport		1.5
Buildings		0.5
Personnel		3.0
	TOTAL	7.0Z

It is not currently the practice to utilize funds generated by local fees for services since the government is supposed to supply these items free of charge. Situations frequently occur in which governmentally provided supplies have been expended, and local monies are available but are not used to purchase critically needed drugs or supplies. Modifications of the system to allow locally generated funds to be used to purchase those items most critically needed at reasonable cost must be given a high priority.

The ability of the Government of Zaire to meet foreign exchange problems associated with importation of vaccines, medications and associated equipment is a separate problem from their ability to mobilize sufficient local currency to meet those same costs. Foreign exchange is limited and decisions must be reached according to the priority the government places on CCCD activities.

(3) Resources Currently Available or Committed

The value of resources presently available or committed for the four-year period 1983-1986, for the implementation of those PHC activities for which CCCD support is being proposed, is 86,000,000 Z (equivalent to $15 million at official exchange rates) from the Government of Zaire, and $565,000 from external donors other than USAID. It is expected that additional resource commitments will be forthcoming from some external donors but estimates of the value of such commitments are not possible at this time.

It is being proposed that the CCCD Project provide $4,849,000 during the four year period 1983-1986 (see Table 6).

(4) Summary of Feasibility

The concept of integrating basic health services and developing a PHC system, an envisioned in the National Health Plan, is logical and sound. Given the availability of resources required to achieve the objectives stated in the National Health Plan, the Country Assessment Team feels that the Plan's objectives relating to PHC, are feasible. The level of support the GOZ has provided to prior EPI efforts, and their planned initiation of user fees, indicate to the assessment team a reasonable expectation that the proposed plan is financially feasible.

2.4.2 Expanded Program on Immunization

A. Background

The National Expanded Program on Immunization in Zaire began in 1977. It succeeded the National Smallpox Eradication Program, which had provided mass immunization for smallpox to all persons, and BCG to persons under 20

years of age. The initial objective of the Zaire EPI was to provide systematic immunization with measles, poliomyelitis, pertussis, diphtheria, tetanus and BCG vaccines to children from birth to 24 months of age residing in the country's 15 largest cities. The program was developed vertically within the Ministry of Health, using the former smallpox eradication strategy of mobile vaccination teams which were directly responsible to the national program headquarters.

Soon after the program began, an effort was made to supplement the mobile strategy by providing additional immunizations through certain fixed primary health care centers. The operational advantage and cost-effectiveness of the fixed center integrated approach soon became evident. In a comparison of children vaccinated by EPI mobile teams and of others vaccinated at fixed PHC centeres, it was found that 66% of children who received the first dose of DTP and polio vaccines at fixed PHC centers completed the series of three doses, as opposed to only 21% who received their first dose from a mobile team.

An international evaluation of the Zaire EPI in 1980 recommended that the mobile strategy be totally abandoned in favor of a fixed center approach with outreach. This recommendation has been accepted by the national EPI leadership and provides the basic strategy for vaccine delivery in the 1982-1986 national EPI plan.

In August, 1981, the EPI leadership, with this strategy in mind, organized and coordinated a joint PHC/EPI training course which was held in Ngidinga, Bas Zaire. The course was attended by chief physicians from 14 different health zones in Zaire. The course used field exercises as part of the training.

The purpose of the three week course was to emphasize the role of the health zone's chief physician in the supervision and training of health personnel responsible for PHC implementation and supervision. One of the main features of the course was the preparation of zonal health plans and the presentation of these plans to the Secretary of the Department of Public Health. The course attracted international attention with representation from EPI/WHO/Geneva, the International Children's Center/Paris and the "Cooperation Medicale Belge". A second course is planned for August, 1982.

The country assessment team visited the pilot rural health zone in which the training took place. The team met with the zone's chief physician and the supervisor of a health center. Both these individuals had attended the joint PHC/EPI training course. Team members were uniformly impressed by the perspective of these individuals and by the extent to which the zonal plan developed during the course was being implemented.

B. Current Achievements

The original five-year EPI plan written in 1977 called for establishment of EPI activities first in Kinshasa, and then their gradual expansion to 14 other urban areas. Through systematic training, through an establishment of a logistics system capable of assuring a reliable supply of vaccination and cold chain equipment, including vaccines; and through capable central leadership and supervision, these goals have been realized. At present the measles vaccination coverage in each of the urban areas is greater than 50% among children 12-24 months of age. An EPI demonstration zone has been established in a rural health zone 2 hours drive from Kinshasa. This demonstration zone is being used for training as EPI expansion continues.

C. Objectives and Targets of Zaire EPI

The 1982-1986 National EPI plan calls for extension of EPI activities to 124 rural health zones by the end of 1986. In addition it advocates integration of all EPI activities into the national PHC system, and describes this integration as a logical evolution of the EPI delivery strategy.

Specific Objectives and targets listed in the 1982-1986 National EPI plan are essentially identical to those listed in the National Health Plan. The following is a list of these objectives and activities:

Objective	Targets
1. Provide vaccine to all PHC posts through planned activities, taking into account local needs and resources.	1.1 Prepare a list of all PHC posts capable of integrating EPI into daily activities.

1.1.1 Planned Expansion to Health Zones:

	1982*	1983	1984	1985	1986
Urban Zone					
New	4	4	3	3	3
Cum. Total	4	8	11	14	17
Rural Zone					
New	14	20	30	30	30
Cum Total	14	34	64	94	124

*includes health zones existing prior to 1982

1.1.2 Percent of Zaire Population Having Access to EPI Services

Zone	Current	Projected 1986
Urban	53%	60%
Rural	6%	58%
National	20.1%	58.6%

1.2 Assist posts in planning sub-regional programs using local baseline data.
1.3 Assist posts in writing workplans for sub-regional activities.
1.4 Assist posts in sub-regional evaluations of EPI activities
1.5 Assure vaccine provision to posts.

2. Assure training of PHC personnel responsible for integration of EPI activities.

2.1 List personnel in need of training.

2.2 Plan training in a well functioning demonstration zone.

2.3 Organize training courses:

2.3.1 Develop training materials to be incorporated into medical and para-medical schools by academic year 1982-1983.

2.3.2 Develop EPI technical manual integrating PHC and EPI for medical and para-medical personnel by July, 1982

2.3.3 Train medical personnel in EPI/PHC integrated activities:

No. of persons to be trained by year

	1982	1983	1984	1985	1986	Total
Medecins Inspecteurs<br>Medecins Sous regionaux	55	10	-	-	-	65
Non-Government Health Personnel	50	40	30	20	10	150
Medecins Chef de Zone	20	30	30	30	30	140
Chefs de Cercle Medical<br>Infermieres Encadreurs	60	90	90	90	90	420
Cold Chain Managers	40	-	40	-	40	120

	2.4 Evaluate Training Effectiveness.
3. Assure uninterrupted supply of vaccines, vaccine equipment and cold chain supplies.	3.1 List equipment and supplies necessary.
	3.2 List equipment and supplies available.
	3.3 Identify sources of financial assistance for equipment and supplies not available.
	3.4 Order and receive equipment and supplies.
	3.5 Assure uninterrupted supply to health posts with integrated EPI/PHC activities.
	3.6 Oversee utilization of vaccine and vaccination equipment.

D. Feasibility

(1) Government Commitment

The EPI Plan of Operations for 1982-1986 is a comprehensive document which is consistent with the National Health Plan. The EPI leadership is enthusiastic when discussing its Plan of Operations and looks forward to implementing the integration of EPI services within the context of Primary Health Care.

Since its inception, the EPI has had to rely heavily on donor support. This circumstance will certainly continue for the forseeable future. In 1981, the Government of Zaire supported at least 25% of the total cost of EPI activities.* It is expected that this level of support will continue even as the delivery of health services is expanded and integrated into a PHC system.

Over the past 5 years the EPI leadership has demonstrated a mature and forward looking attitude concerning its need for continued donor support by actively keeping donors and potential donors informed of its activities, plans and needs. The formation in 1980 of the Comite Directeur du PEV** is but one example of this.

---

* There are several different accounting procedures that can be utilized. The accounting procedure which is least favorable to the GOZ would still show the GOZ contributing at least 25% of the total cost. Other procedures would show the GOZ contribution to be 50% or higher.

** The EPI Steering Committee, whose members represent major donors to EPI, representatives of the Department of Public Health, and interested observers. The Committee meets quarterly with the EPI leadership to discuss program progress and future plans.Based on the prominence of EPI in the National Health Plan, on the detailed and well conceived EPI Plan of Operations, on past and projected levels of Government of Zaire financial support to EPI, on the proven ability of the EPI leadership to attract external resources, and on the past performance of the EPI leadership; the Country Assessment Team feels that the Government of Zaire's commitment to EPI is assured.

(2) Resources Required

Over the five year period 1982-1986, delivery of EPI services will be greatly increased in Zaire. This expansion will take place within the context of Primary Health Care. A major concern is how EPI will maintain its present high standards of vaccine supply and supervision as delivery of its services are expanded. Greatly increased amounts of equipment, including vehicles, will be required. Large numbers of PHC personnel will require in-service training and supervision. The expected increase of resources needed to fund this expansion has been explicitly outlined in the EPI Plan of Operations for 1982-1986. Table 2 is taken from that plan (some numbers have been rounded off). While the country assessment team feels that some of the estimates contained in the table need to be re-assessed, it is agreed that in the aggregate the table represents a reasonable statement of needs to enable realization of the goal of implementation and/or maintenance of services in 17 urban and 124 rural health zones by 1986.

Since immunization services will be integrated into the delivery of basic health services performed by existing staff, there will be no need for additional vaccinators. There will be additional personnel needed at the national and regional level for the administration, supervision and evaluation of activities, and for logistical support, but it is expected that these needs can be met by the training of existing Department of Public Health personnel not presently working with the EPI. Expanded personnel needs for the implementation of integrated PHC activities will almost wholly be met by utilizing and retraining, as necessary, personnel already employed by the health sector. Since the development and refinement of training materials and training activities has been in process since 1981, there seems to be no doubt of the Government's recognition of this need, and its capability to respond.

A technical officer such as the one currently provided to the Zaire EPI under a USAID-CDC PASA would appear to be a real need. Besides providing technical expertise, such a person could serve a valuable role in the establishment of health zones and with coordinating the implementation of CCCD activities with other PHC activities. As implementation of PHC systems proceed, administrative, supervisory and logistical support to EPI will more correctly be seen as support for the integrated PHC system.

(3) Resources Currently Available/Committed

Since 1977, USAID has supported the EPI with a full-time technical officer under a PASA from CDC. The technical officer is assigned specifically to advise the national EPI in its measles control efforts. The TO also works with national EPI staff in planning, implementation and evaluation of all other EPI activities. Under the PASA, USAID supplies measles vaccine, vaccination equipment, vehicles and other commodities. Significant USAID financial assistance is also provided to the Zaire EPI from counterpart

TABLE 2

PROJECTED EPI NEEDS ACCORDING TO EPI NATIONAL PLAN OF ACTION, 1982-198[illegible]

ITEM		YEAR 1982	1983	1984	1985	1986
BCG VACCINE	DOSES	2,325,000	2,400,000	2,500,000	2,540,000	2,620,000
	VALUE	$75,000	$84,000	$96,000	$110,000	$125,000
DTP VACCINE	DOSES	2,035,000	2,685,000	3,620,000	4,435,000	5,355,000
	VALUE	$80,000	$115,000	$170,000	$230,000	$305,000
POLIO VACCINE	DOSES	2,035,000	2,685,000	3,620,000	4,435,000	5,355,000
	VALUE	$116,000	$170,000	$250,000	$340,000	$450,000
TT VACCINE	DOSES	1,640,000	1,900,000	2,255,000	2,610,000	2,990,000
	VALUE	$48,000	$61,000	$80,000	$102,000	$130,000
MEASLES	DOSES	680,000	895,000	1,205,000	1,480,000	1,785,000
	VALUE	$150,000	$220,000	$325,000	$436,000	$580,000
NEEDLES	DOZENS	2386	2811	3408	3950	4552
	VALUE	$1,000	$1250	$1650	$2100	$2650
SYRINGES	NUMBER	9542	11424	13630	15798	18200
	VALUE	$11,000	$14,000	$18,500	$23,250	$29,500
REFRIGERATORS	NUMBER	90	120	165	165	165
	VALUE	$70,000	$102,000	$154,000	$170,000	$186,000
COLD BOXES	NUMBER	720	960	1320	1320	1320
	VALUE	$20,000	$30,000	$44,000	$49,000	$54,000
ICE PACKS	NUMBER	5,760	7,680	10,560	10,560	10,560
	VALUE	$6,500	$10,000	$14,000	$15,000	$17,000
BICYCLES	NUMBER	560	800	1,200	1,200	1,200
	VALUE	$62,000	$97,000	$160,000	$176,000	$195,000
VEHICLES/PARTS	NUMBER	18	24	33	33	33
	VALUE	$396,000	$580,000	$880,000	$967,000	$1,065,000

funds* which are used to pay the contractual personnel required for central coordination and supervision.

Other vehicles, cold chain and vaccination equipment, and vaccines have been provided by WHO and UNICEF. The Belgian Government, through its "Cooperation Medicale Belge" provides one full-time technical officer in Lubumbashi, temporary training advisors and vaccines. Oxfam will provide bicycles in 1982. Table 3 was compiled by the country assessment team. It represents a current best estimate of donor commitments (excluding USAID) to EPI for the period 1982-1986.

TABLE 3

Anticipated Commodity Support for EPI Zaire 1982-1986
(Donors other than USAID)

Donor	Item	Year 1982	1983	1984	1985	1986
	Vaccine:					
UNICEF	BCG (doses)	3,075,000	7,075,000	NA	NA	NA
UNICEF	DTP (doses)	1,500,000	3,102,000	NA	NA	NA
UNICEF	OPV (doses)	1,200,000	2,000,000	NA	NA	NA
UNICEF	TT (doses)	1,800,000	2,160,000	NA	NA	NA
UNICEF	Needles (S/C)	630 doz	NA	NA	NA	NA
UNICEF	Syringes (2cc)	3,600	NA	NA	NA	NA
UNICEF	Syringes (BCG)	1,000	NA	NA	NA	NA
UNICEF	Refrigerators	35	NA	NA	NA	NA
WHO	Refrigerators	2	NA	NA	NA	NA
UNICEF	Vehicles	-	2	6	7	NA
OXFAM	Bicycles	100	NA	NA	NA	NA

Note: NA means "not available", support may be forthcoming but was not certain at the time of the country assessment.

The EPI Plan of Operations for 1982-1986 estimates in its Table 16 that the total cost of plan implementation will be $30.6m ($26.6 for 1983-1986). The projected GOZ contribution for 1983-1986 (exchange rate of 5.65 Z's/dollar) is $14.6 m. While the short-fall is substantial, it is not unreasonable to expect that it can be met through external support. Committed support from USAID, WHO-UNICEF and the Belgian Government already exceeds $1 m. Additional support of at least $2-3 million is expected between now and the end of 1986.

---

*Counterpart funds are available in local currency only and can be approved by the USAID Mission Director in response to a specific government proposal for financial assistance. The source of counterpart funds is local currency generated by the sale of PL 480 commodities.

(4) Summary of Feasibility

The EPI Plan of Operations for 1982-1986 is a comprehensive and logical document. The expansion that it envisions is based on, and is consistent with what is stated in the National Health Plan with regard to EPI and PHC.

Since its inception in 1977, the Zaire EPI has had a commendable record for accomplishing stated objectives. While the stated objectives for 1982-1986 are extremely ambitious, the country assessment team feels that the EPI leadership will be able to accomplish these objectives, given the resources required to do so. Should the amount of resources needed to fully implement the EPI plan of Operations not be forthcoming, a corresponding reduction in the accomplishment of Plan objectives will likely be the result.

2.4.3 Control of Diarrheal Diseases

A. Background

In February 1982, a WHO consultant from CDD/Geneva cooperated with Department of Public Health officials to prepare an outline plan for a national CDD program. The outline plan supports integration of diarrheal disease control strategies within the national PHC structure, with oral rehydration therapy to become one of the basic services provided by primary health care centers. The outline plan envisions use of the present administrative, supervisory and logistical infrastructure of EPI for CDD's activities. According to the outline plan, expansion of CDD activities would parallel those of EPI, with coverage catching up to EPI by 1986.

B. Current Achievements

The Department of Public Health has officially given the responsibility for CDD activities in Zaire to the EPI. The outline plan of operations drafted in February has not yet been developed into a comprehensive and official government document, but the EPI leadership expects that the task of developing a comprehensive CDD plan of operations similar to the existing plan for EPI will be completed by September 1982. The Zaire EPI has already designated a Zairois physician to be responsible for CDD activities. This individual will function within the technical services branch of the EPI.

The Government of Zaire has appointed a technical advisor for CDD. This individual is the Chairman of the Department of Microbiology at the University of Kinshasa Faculty of Medicine.

C. Objectives and Targets of Zaire CDD

The CDD outline plan has been endorsed by the EPI leadership and is consistent with the National Health Plan. The objectives and activities contained in the outline plan for CDD operations are listed in the table below:

OBJECTIVE	TARGETS
1. Assure training of PHC personnel responsible for integration of CDD	1.1 National staff to attend CDD Program Manager's Course (WHO) in 1982<br><br>1.2 Regional health center and health zone personnel to be trained at integrated PHC course, 1982.<br><br>1.3 Additional health centers and health zone personnel training at CDD Mid-level course (WHO) in 1983.<br><br>1.4 Develop technical manual for in-service training in 1983.
2. Provide ORS packets to all PHC centers.	2.1 Develop integrated ORS strategy system within EPI vaccine delivery strategy to PHC centers, 1983<br><br>2.2 Begin ORS use in Kinshasa, 1983.<br><br>2.3 Begin ORS use in Kisantu, 1983.<br><br>2.4 Extend use of ORS to other urban and rural health zones, 1983<br><br>2.4.1 Access to ORS: 60% of all childhood diarrheal diseases by 1986.<br><br>2.4.2 ORS Coverage: 80% of all childhood diarrheal cases with access, 48% of all diarrheal cases (60% x 80%) by 1986.<br><br>2.4.3 Mortality Reduction: Assuming 50% effectiveness, reduce childhood diarrheal deaths 24% (48% x 50%) by 1986.
3. Develop integrated information system for CDD.	3.1 Establish diarrheal disease morbidity and mortality rates through survey, 1983.<br><br>3.2 Establish routine and sentinel CDD surveillance, 1983.
4. Begin national production of ORS packets, 1985*.	

*It will probably be necessary to import the ORS ingredients and the packaging. If this is the case, local production will most accurately be described as local packaging. To the extent possible, ingredients and packaging will be produced locally.

D. Feasibility

(1) Government Commitment

The cholera outbreaks of 1978 and 1979 drew national attention to the problem of diarrheal diseases in Zaire. At that time a Government unit was formed and made responsible for controlling cholera. The focus that this unit provided for diarrheal diseases tended to weaken as the threat of cholera subsided.

The prominence given to CDD by the National Health Plan as a health priority is evidence that the Government of Zaire recognizes the need to institutionalize a focus for diarrheal disease control in the country. The National Health Plan's inclusion of CDD objectives as part of the overall primary health care objectives attests to the long-term nature of the Government's commitment to diarrheal disease control activities. The Government's acceptance of the outline Plan of Operations for CDD drafted in February, its subsequent designation of EPI as the program to have responsibility for CDD activities, and the intention of the EPI leadership to develop a comprehensive CDD Plan of Operations by September 1982, can all be seen as positive indications of the Government's commitment to CDD.

Further evidence of a Government commitment to CDD activities lies with the protocol recently submitted by the Government to WHO for the study of diarrheal disease etiology, which includes a request for extensive laboratory equipment and a plan for a study of the effectiveness of oral rehydration in the pediatric ward of the teaching hospital.

(2) Resources Required

In addition to the funding required for establishment of a laboratory and an oral rehydration unit in Kinshasa, many other resources, including ORS, IV equipment and solutions, and in-service training, will be required. Because the CDD outline plan envisions using the EPI structure for delivery of ORS packets and supervision, many of the required resources will be the same as those listed in this report for EPI, in Section 2.4.2 Part D (2), Resources Required. The CDD outline plan assumes that 10% of the costs of transport and 30% of other operating costs listed in the EPI Plan of Operations for 1982-1986 will be attributable to CDD.*

The CDD outline plan estimates that approximately 50% of program costs will be for ORS packets, including the materials and equipment needed for packaging. The outline plan does not include an item for IV equipment, under the assumption that increased coverage by oral rehydration therapy will actually

---

*These percentages represent a reasonable estimate. After CDD activities have been implemented, operating costs will need to be reviewed in terms of the proportions appropriately attributable to EPI and CDD. If operational experience indicates that the 10% and 30% estimates are not reasonable, the percentages should be adjusted accordingly.

decrease country-wide IV needs. While it is expected that one benefit of increased coverage by oral rehydration therapy will be a decreased dependence on IV therapy, the fact remains that an IV capability will be required for at least the reference hospital in each health zone. Severe cases of dehydration will be referred to this unit and the unit must have the means of providing treatment. Most, if not all, of the facilities that will become reference hospitals probably already have IV equipment and solutions. Since this cannot be finally determined until all health zones have been defined and all reference hospitals designated, and since it may be necessary to establish an IV capability in some facilities other than reference hospitals, the possibility exists that more IV equipment and solutions than are presently available will be necessary if CDD coverage is to equal EPI coverage by 1986.

Tables 4 and 5 provide an estimate of the number of ORS packets and a notion for how extensive an IV capability would be necessary to fully implement CDD in Zaire according to the outline plan.

Table 4

Estimated Number of ORS Packets Required for CDD Zaire

	1982	1983	1984	1985	1986
Total Zaire Population	28,120,000	28,935,000	29,775,000	30,640,000	31,525,000
Zaire population under five (19%)	5,432,800	5,497,650	5,657,250	5,821,600	5,989,750
No. under 5 diarrhea cases requiring ORT yr (2.5)	13,357,000	13,744,125	14,143,125	14,554,000	14,974,375
Coverage of under 5 population*	667,850	2,061,619	4,242,938	6,549,300	8,984,625
x2 packets/case	1,335,700	4,123,238	8,485,876	13,098,600	17,969,250
x1.33 (wastage and use on older patients)					
=No ORS packets required	1,776,481	5,483,907	11,286,215	17,421,138	23,899,102

*5% 1982, 15% 1983 30% 1984 45% 1985 60% 1986

Table 5

Number of Health Zones (Urban plus Rural) to be Operational in Zaire, 1982-1986

1982	1983	1984	1985	1986
18	42	75	108	141

Note: Assume that IV capability needed in at least one health facility per health zone.

(3) Resources Currently Available/Committed

The cost of ORS packets and operating costs account for virtually 100% of anticipated CDD future program costs (excluding costs of IV fluids and equipment). It is assumed that UNICEF will provide at least partial needs for ORS packets, but no commitment has yet been made. A joint Belgium-Zaire project to establish a manufacturing capability in Zaire for first priority drugs may be able to assist with meeting program needs. Other donors may also be willing to help meet ORS packet needs.

As pointed out in section (2) above, the CDD outline plan assumes that a certain proportion of transport and other operating costs for EPI will be attributable to CDD. CDD success in acquiring needed resources is thus dependent on the extent to which EPI is able to attract required resources. The ability of EPI to attract necessary resources for planned activities has been discussed under Section 2.4.2, Part D (3), Resources Currently Available/Committed. In that discussion it was shown that more than 50% of projected costs for the period 1983-1986 are covered by Government commitments and that there is good reason to believe that most, if not all, of the shortfall will be covered by external assistance.

(4) Summary of Feasibility

The National Health Plan lists CDD as a priority and calls for its implementation within the context of the PHC system. An outline plan for CDD was developed by a WHO Geneva consultant in February 1982. The Government has officially delegated responsibility for CDD activities to the EPI. The EPI leadership expects to have a comprehensive CDD Plan of Operations by September 1982.

The strategy for developing CDD in Zaire will be based on the EPI strategy. CDD activities will be integrated with EPI activities. What has been said about EPI activities with regard to the feasibility of achieving the objectives stated in its Plan of Operations for 1982-1986, applies to CDD as well. That is, the EPI leadership will be able to accomplish its objectives, given the resources required to do so.

2.4.4 Malaria Control

A. Background

The present National Malaria Control project (PLAP) was begun in 1976. The original PLAP plan emphasized vector control by insecticide spraying in 2 project areas; one in Kinshasa, one in a rural area. In 1980, a small program of presumptive chloroquine treatment for fever among children less than 15 years of age was begun in the two project areas. The present PLAP plan (the National Malaria Control Plan of Operations for 1982-1983) supports activities only in Kinshasa.

USAID support (both bilateral and counterpart funds) to the PLAP represented almost all the PLAP's 1981 budget. Government of Zaire support for the PLAP in 1981 was less than 5% of the total budget.

The future of the PLAP is uncertain. USAID support to the PLAP was terminated as of June 1982, because the current strategy of vector control is not cost-effective in rural areas and because of lack of official interest and commitment by the GOZ. The 74 (out of 94) full-time PLAP personnel supported by USAID funding had their employment terminated at that time. The Government of Zaire has provided no indication that it intends to provide further support to the PLAP.

B. Current Achievements

By January 1982, malaria control activities, essentially aimed at vector control by residual spraying with DDT, were operational in two project areas: one was urban (Kinshasa) and one rural (Kisantu Rural Health Zone). Combined, the two areas served less than 2% of the total Zaire population. Malariometric evaluation (point prevalence studies by thick blood smear) in the project areas have shown a decrease in malaria prevalence as compared to adjacent control areas. Reduction of prevalence in the rural project area was greatest, approaching a reduction of 35%.

Other studies coordinated by the National Malaria Control Program have shown that 50% of all children less than 15 years of age who seek medical treatment at PHC centers in the project areas have a chief complaint of fever. Among these children approximately 28% have malaria parasites in capillary blood confirmed by thick smear.

C. Objectives and Targets of Zaire Malaria Control

The PLAP has a plan of operations for 1982-1983 which outlines support for activities in Kinshasa. Since USAID support to the PLAP has been terminated, and since the Government of Zaire is not supporting the PLAP, the future of the PLAP is doubtful. This being the case, it is probably not useful to use goals stated in the PLAP plan of operations as an indicator of planned Government of Zaire malaria control activities.

Under the assumption that the PLAP will not continue to exist, the country assessment team recognizes that any malaria control activities that are to be supported by CCCD must become the responsibility of some other existing government health program. Because CCCD support for immunization and diarrheal disease control activities is to be provided through the existing EPI infrastructure it would make sense to provide support to malaria control activities through the same infrastructure. In the absence of specific guidelines from the Government of Zaire on where the organizational focus for CCCD supported malaria control activities should lie, a CDC malaria consultant recommended in June 1982, that a core of expertise from the PLAP containing program elements pertinent to CCCD be retained and shifted to the EPI infrastructure. It is of course recognized that the final decision on where these malaria control responsibilites will lie rests with the Government of Zaire.

Regardless of where the organizational focus for CCCD supported malaria control efforts is finally located, the objective of these efforts will be to decrease mortality among children less than 5 years of age due to acute malaria and to decrease fetal wastage and the number of low birth weight newborns. This objective will be realized through:

- Presumptive malaria treatment for children with fever
- Operational research and evaluation, including the following topics, among others:
  - Drug resistance
  - Cost-effectiveness of malaria prophylaxis of pregnant women
  - Effect of anti-malarial activities on infant mortality
  - Effect of anti-malarial activities on the mother, and on the fetus and the newborn
  - Efficacy of the diagnosis and treatment provided by different levels of field personnel.

Presumptive treatment of children with presumed malaria and malaria prophylaxis of pregnant women (where cost-effective to do so) will be given in hospitals, health centers and health posts in much the same way as oral rehydration therapy. It is expected that malaria presumptive treatment will also be expanded to the village level, but decisions concerning the feasibility, overall effectiveness, and preferred methods for delivering anti-malarials will be made following an initial two-year surveillance phase in an area with about 250,000 - 500,000 persons (2-4 rural health zones).

If the organizational focus for CCCD malaria control support does become the EPI, it will be through the EPI infrastructure that commodities for malaria control activities are delivered. EPI would also provide the focus for training, surveillance, evaluation and health education activities. Many aspects of the malaria work, including operational research, will require some special expertise in malariology which EPI does not presently have. The shifting of certain PLAP program elements to EPI would rectify most of these deficiencies. Remaining deficiencies could be rectified through training and CCCD supported consultations.

D. <u>Feasibility</u>

(1) <u>Government Commitment</u>

It seems clear that there is no Government of Zaire commitment for the PLAP. While malaria is recognized by the GOZ as a major cause of morbidity and mortality, at the time of the country assessment there was no official government statement with regard to a Government commitment to implement, carry out and maintain the type of malaria control activities that CCCD is proposing to support. With regard to the proposal that EPI assume the responsibility for these malaria control activities, the EPI leadership has offered to provide interim office space and administrative support for the core malaria personnel recommended by the CDC consultant if the Government of

Zaire decides that the EPI should assume those responsibilities. Informal discussions on this subject between the PLAP and EPI directors have already taken place.

The country assessment team feels that there will be a Government commitment for the type of malaria control activities that CCCD proposes to support. The extent of the Government commitment will become clear once the program focus for these activities is identified. If it is the EPI that is given the responsibility, the integration of these malaria control activities into PHC activities would be greatly facilitated.

(2) Resources Required

If CCCD supported malaria control activities are channeled through the EPI, add-on costs to those already anticipated for EPI and CDD activities would be minimal. Add-on costs would be almost exclusively limited to the cost of anti-malarial drugs. Costs for presumptive treatment for children under five in health zones implementing integrated primary health care services are estimated to be $435,000 for the four year period 1983-1986. Of this amount the Government of Zaire would be responsible for the local currency equivalent of $145,000 (see Table 6).

Providing malaria prophylaxis for pregnant women will be considerably more expensive. The cost of providing this service in all the areas projected to have integrated PHC services by 1986 is estimated at more than $4 million during the four year period 1983-1986.

(3) Resources Currently Available/Committed

Assuming EPI resonsibility for proposed CCCD supported malaria activities, the resources needed are for all practical purposes limited to the add-on cost of anti-malarial drugs. The cost of anti-malarials for presumptive treatment for children is modest and should be met. CCCD is proposing to cover 67% of the total cost projected for anti-malarials for 1983-1986. With counterpart fund support, it seems reasonable that the Government of Zaire would be able to meet the remaining 33%.

For the cost of anti-malarials for prophylactic treatment of pregnant women, the situation is different. The Government of Zaire has indicated that it is not willing to assume, by 1986, the obligation to provide the anti-malarials for all pregnant women. The Government recognizes the proven benefits of the strategy, but is not ready to assume the financial obligation of providing 100% of all chloroquine tablets by 1986. The Government of Zaire feels that there might be a more cost-effective strategy. It would like to see the benefits of prophylaxis vs. treatment of pregnant women be carefully assessed. The country assessment team is recommending that malaria prophylaxis as well as other malaria related operational research questions be assessed in an operational research component of CCCD malaria control activities. The cost to the Government of Zaire would be 250,000 Z per year for 1983 and 1984. With counterpart fund support, the Government of Zaire should be able to provide these financial resources.

(4) Summary of Feasibility

Within the context of a PHC system for delivery of integrated health services, as outlined in the National Health Plan, the feasibility of providing presumptive malaria treatment for children under 5 would seem to be very high. This is especially true if it is the EPI through which this malaria control strategy would be implemented. The technology is relatively simple and the add-on costs are modest.

With respect to malaria chemoprophylaxis for pregnant women, the country assessment team is impressed by the caution that the Government of Zaire is showing with regard to assuming the obligation for the cost of a strategy that it cannot yet afford, and is not sure may be the most cost-effective. The country assessment team feels that there is much need for operational research on the economic and logistic aspects of this approach and is anxious to support and cooperate with such research. The projected cost of operational research on the cost-effectiveness of prophylactic malaria treatment for pregnant women and related operational research is modest. The country assessment team feels the it is feasible to carry out the operational research successfully.

3. Proposals for CCCD in Zaire

3.1 Proposed Scope of CCCD in Zaire

A review of existing hospital data on childhood infectious disease morbidity and mortality from Zaire has confirmed that measles, diarrheal diseases and malaria represent a significant proportion of under 5 morbidity and mortality. The country assessment team reviewed hospital registers in 3 zones of Zaire and found that these diseases were responsible for approximately 40-50% of pediatric hospital admissions and over 35% of all pediatric deaths. (See Table 1.)

The National Health Plan acknowledges the priority status of these diseases and recommends that measures be taken to bring them under control. Measles and diarrheal diseases can be addressed through the establishment of a system of integrated Primary Health Care services, as proposed by the Plan. Control of malaria may require extraordinary measures, but certain aspects of malaria control, such as presumptive treatment and chemoprophylaxis of pregnant females, fit well within the PHC integrated concept.

CCCD can best support efforts to control measles, diarrheal diseases and malaria in Zaire by supporting the establishment of an integrated PHC system. CCCD can provide needed support in many areas, but since it is likely that CCCD support would be only part of overall donor support, the importance of Government coordination of donor support cannot be overemphasized. Without such coordination, wasteful duplication of effort may result; and progress toward development of a unified, national PHC system will surely be slowed.

It is proposed by the country assessment team that CCCD provide, as appropriate, both regional and bilateral support to Zaire for EPI, diarrheal disease and malaria treatment activities. It is the judgement of the country assessment team that the three major public health problems of measles, diarrheal diseases and malaria can best be addressed through the integrated PHC approach outlined in the National Health Plan. Measles would be included in EPI activities, diarrheal disease mortality would be addressed through CDD activities and those aspects of malaria control activities that lend themselves to the PHC approach would be included in the services offered by the PHC system.

Through bilateral technical cooperation, CCCD can provide, as appropriate, commodities needed to carry out EPI, diarrheal disease and malaria treatment activities within the context of PHC. Also as part of bilateral technical cooperation, CCCD can support national training courses. Through the CCCD regional support project, assistance can also be offered for training, training development/adaptation, health education/promotion, operations research, and strengthening of health information systems.

The final two sections of this report present proposals for CCCD regional assistance and bilateral technical cooperation to Zaire for EPI, diarrheal diseases and malaria control activities.

3.2 Proposed CCCD Regional Support Project Assistance

The following CCCD regional assistance activities are proposed for 1982-1986 in support of EPI activities and efforts to control diarrheal diseases and malaria. Since it is through the PHC system that these efforts will be made, support for implementation of an integrated PHC system is seen as supporting the more specific objectives of measles, diarrheal diseases and malaria control.

In its National Health Plan, the Government of Zaire has already endorsed utilization of integrated PHC services as the best means of carrying out EPI activities and diarrheal disease control. Such a Government commitment does not presently exist for malaria control; however, treatment for malaria does exist at government and private clinics when drugs are available. The country assessment team feels that a reduction of malaria mortality may be feasible through use of the PHC system for administration of anti-malarial drugs and would support, given Government approval, implementation of presumptive anti-malaria treatment for children under 5 in health zones offering integrated PHC services, and operations research on the cost-effectiveness of chemoprophylaxis of pregnant women vs. presumptive treatment as a means of reducing fetal wastage and the number of low birth weight newborns.

Category	Objective/Activity	Time Target	Responsible for Action
1. Training	1.1 Sponsor interregional participants for 1983 and 1984 integrated PHC training courses.	August, 1983 1984 (date not set)	Regional Coordinator Brazzaville

2. Training Development/ Adaptation	2.1 Assist with effort to develop/adapt integrated PHC course materials so that they can be used throughout Zaire	1982 and 1983	Training Consultant designated by CDC/ Atlanta
	2.2 Assist with development/ adaptation/ of EPI, CDD and malaria training materials that can be incorporated into technical manual stressing integrated approach.	1982 and 1983	Training Consultant designated by CDC Atlanta
	2.3 Assist with development/ adaptation of EPI, CDD and malaria technical and training materials for inclusion in medical and paramedical school curricula.	1982 and 1983	Medical Epidemiologist assigned to Zaire
	2.4 Assist with development of technics to evaluate training efficacy.	1982 and 1983	Training Consultant designated by CDC/ Atlanta
3. Health Education/Promotion	3.1 Develop and improve technics for promoting PHC initiative through "animateurs".	1983<br>1984	Training consultant designated by CDC Atlanta
	3.2 Develop materials and methods to educate mothers and pregnant women on implications of fever and need to avail themselves of an appropriate drug	develop early 1983<br>utilize late 1983	Health Educator
4. Operations Research	4.1 Support, as appropriate, and as authorized by review committee<br>Possible topics:<br>-Hepatitis B transmission among children vaccinated with needle and syringe<br>-Drug sensitivity to chloroquine pyrimethamine in P. falciparum in program areas prior to start of presumptive treatment and annually thereafter	1983<br>1984<br>1985	Technical Officer Assigned to Zaire. Medical Epidemiologist responsible for Zaire

	-Feasibility of cost-effective production of anti-malarials<br>-Assess quality of locally manufactured anti-malarials (if production commenced)<br>-Selected topics for diarrheal disease control<br>-Feasibility of presumptive malaria treatment in integrated PHC setting.<br>-Feasibility and cost-effectiveness of malaria chemoprophylaxis in pregnant women in an integrated PHC setting.<br>-Use of village "animateurs" as malaria detection and treatment agents<br>-Efficacy of the diagnosis and treatment provided by different levels of field personnel		
5. Health Information Systems	5. Develop/improve survey techniques for establishing baseline morbidity and mortality data-including seasonal and geographical patterns for malaria transmission.	1983<br>1984	Medical Epidemiologist

In addition to the above CCCD components, it is proposed that a full time CCCD technical officer be assigned to Zaire and that a Medical Epidemiologist have part-time responsibility for Zaire. Both these individuals will be paid from regional CCCD funds.

3.3 Proposed CCCD Bilateral Assistance

CCCD bilateral assistance to Zaire is proposed for 1983-1986 in support of efforts to control measles, diarrheal diseases and malaria. As is the case with regional assistance, support is proposed within the context of an integrated PHC system.

CCCD bilateral assistance is proposed under individual CCCD components. The CCCD components listed below are the same as those presented under Proposed CCCD Regional Assistance except that an additional component, Program Evaluation, has been added. Travel and per diem for external evaluators supported by CCCD will be paid from regional CCCD funds.

The bilateral assistance proposal includes a proposal for CCCD commodity support. This proposal is based on projected expansion of integrated PHC services and anticipated support from donors other than USAID. It is also based on anticipated support to primary health care activities through the recently initiated Basic Rural Health project of USAID (660-0086). This bilateral project is administered by the medical office of the E.C.Z. (Eglise du Christ au Zaire) which is the Council of Protestant communities within Zaire. This project will provide assistance in 50 rural health zones to support primary health care activities, i.e. 1/3 of the total 140 urban and

rural health zones targeted for integrated PHC activities by 1986 in the National Health Plan. Close collaboration between the Basic Rural Health and CCCD projects will eliminate unnecessary duplication of commodity supply and activities.

CCCD Component	Objective Activity	Time Target	Responsible for Action
1. Training	Participate in integrated PHC training course.	annually	T.O. assigned to Zaire.
2. Training Develop- ment/ Adapta- tion	Assist with de- velopment and adap- tation of PHC course materials	1982-1983	T.O. assigned to Zaire
3. Health Education/ Promotion	(See proposed regional activities)		
4. Operations Research	(See proposed regional activities)		

CCCD Component	Objective Activity	Time Target	Responsible for Action
5. Health Informa- tion Systems	Promote appropriate data collection, analysis and utilization for program planning/ monitoring	on-going	T.O. assigned to Zaire, ME responsible to Zaire
6. Program Evalua- tion tion	Strengthen and utilize appro- priate indicators for program moni- toring/program direction	on-going	T.O. assigned to Zaire
	Participate in program evaluations as requested or as appropriate.		T.O. assigned to Zaire

Table 6 presents proposed CCCD bilateral commodity support to Zaire for 1983-1986. Attention is called to the phased reduction in commodity support as a percentage of estimated needs for the recurrent items of measles vaccine and needles and syringes. For these items support will begin at 100% of estimated needs and will reduce by 25% each succeeding year. This phased reduction in support will enable implementation of EPI activities at the level projected in the EPI Plan of Operations, but by the second year of support will begin shifting the burden of recurrent costs to the Government of Zaire; thereby encouraging subsequent expansion of activities to be kept at a level that the combined public and private sectors can maintain.

For durable commodity items such as vehicles and cold chain equipment a fixed percentage of estimated needs is being proposed.

With respect to recurrent items, the Project Agreement will contain language to the effect that the percentage commitments will be tied to actual needs rather than projected needs. Thus, if the implementation of PHC activities does not take place as rapidly as projected (from 1982 to 1986) an increased of from 20% to 60% of the total Zaire population served or an increase of roughly 10% per year) commodities needed for PHC implementation will be less than projected and CCCD commodity assistance would be correspondingly reduced to conform with revised estimates of actual needs.

Table 6
Proposed CCCD Bilateral Commodity Support, 1983-1986

ITEM	FISCAL YEAR 1983	1984	1985	1986
Measles Vaccine (Doses)	1,000,000	900,000	750,000	450,000
	@ 27¢	@ 30¢	@ 33¢	@ 36¢
Cost	$270,000	$270,000	$247,000	$162,000
% Estimated Needs	100%	75%	50%	25%
Syringes	100,000	87,500	75,000	50,000
	@ $1.40	@ $1.60	@ $1.80	@ $2.00
[illegible]	$140,000	$140,000	$135,000	$100,000
% Estimated Needs	100%	75%	50%	25%
Needles (dozens)	33,350	29,200	25,000	16,700
	@ 75¢	@ 90¢	@ $1.05	@ $1.20
Cost	$25,000	$26,000	$26,000	$20,000
% Estimated Needs	100%	75%	50%	25%
Vehicles/Parts	18	24	18	18
	@ $18,000	@ $20,000	@ $22,000	@ $24,000
Cost	$324,000	$480,000	$396,000	$432,000
% Estimated Needs	75%	75%	60%	60%
Motorbikes (including parts/helmets)	50	50	-	-
	@ $500.00	@ $550.00		
Cost	$25,000	$27,500	-	-
Use of Private Plane	$18,000	$18,000	$18,000	$18,000
Refrigerators	90	125	125	125
	@ $600.00	@ $660.00	@ $725.00	@ $800.00
Cost	$54,000	$82,500	$91,000	$100,000
% Estimated Needs	75%	75%	75%	75%
Vaccine carriers	750	650	300	300
	@ $22.00	@ $24.00	@ $26.00	@ $28.00
Cost	$16,000	$15,600	$7,800	$8,400
Ice Packs	3,750	3,250	1,500	1,500
	@ .80¢	@ .85¢	@ .90¢	@ .95¢
Cost	$3,000	$2,800	$1,400	$1,400
Cold Chain Contingency	$10,000	$10,000	$10,000	$10,000
ORS Packets	$50,000	$50,000	-	-
IV Equipment and solutions	$50,000	$50,000	-	-
Anti-malarial drugs*	$20,000	$55,000	$90,000	$125,000
Laboratory Equipment	$15,000	$15,000	$15,000	$15,000
Single Side Band tranceivers	14	-	-	-
	@ $5,500	-	-	-
Cost	$77,000	-	-	-
Antenna system for radio HQ	$1,000	-	-	-
Photocopy machine	$5,000	-	-	-
Mimeograph equipment	$3,000	-	-	-
Electric typewriters	2	-	-	-
	@ $1,350	-	-	-
Cost	$2,700	-	-	-
Megaphone	150	100	50	-
	@ $75.00	@ $80.00	@ $85.00	-
Cost	$11,250	$8,000	$4,250	
Loudspeaker system for vehicles	10	-	-	-
	@ $250	-	-	-
Cost	$2,500	-	-	-
SUB-TOTAL	1,122,950	1,250,400	1,041,950	991,800
10% contingency	113,000	125,000	105,000	99,000
TOTAL	1,235,950	1,375,400	1,146,950	1,090,800

*Most anti-malarial drugs to be obtained through GOZ and counterpart funds locally (810,000 Z)

ANNEX 1

Persons and Documents Consulted

Persons Consulted

Dr. Kalisa-Ruti, Director, EPI, Zaire
Dr. Mambu Ma Disu, Chief of Technical Services, EPI Zaire
Citoyen Kapitaine Khantaway, EPI Zaire
Citoyen Ilunga Katenda, EPI Zaire
Mr. Norman Sweet, Director, USAID, Zaire
Mr. Walter Boehm, Assistant Director USAID, Zaire
Mr. Richard Thornton, Acting PHO, USAID Zaire
Mr. Derrick Singer, Congo Liason, USAID Zaire
Mr. Robert Hogan, CDD/WHO/Geneva
Mr. Gomez, UNICEF Representative, Zaire
Dr. Zanotto, WHO EPI Advisor, Zaire
Dr. Makengo, Director, Zaire Malaria Control Program (PLAP)
Mr. Robert Turner, USAID Advisor, PLAP, Zaire
Dr. Jack Sexton, CDC/USAID Epidemiology Advisor, PLAP, Zaire
Dr. Miatudila, Government of Zaire Liason, USAID/ECZ Project
Dr. Frank Baer, Project Manager, USAID/ECZ Project
Sister Emily Mullen, Public Health Nurse, Ngidinga Maternity Dispensary
Dr. Muyemebe-Tamfum, Chairman, Department of Microbiology, University of Kinshasa
Dr. Omana, Professor of Pediatrics, University of Kinshasa
Citoyenne Karomba Xavierene, Statistician, EPI Zaire
Mr. Harry Godfrey, CDC/USAID Technical Advisor, EPI Zaire
Mr. Marc Szczeniowski, WHO Monkeypox Advisor, Zaire
Dr. Jean-Francois Ruppol, Chief, Belgian Medical Mission

Documents Consulted

National Health Plan, 1982-1986
National Plan of Actions, EPI Zaire 1982-1986
Outline Plan of Operations, CDD Zaire
National Malaria Control Plan for Extension of Activities in Kinshasa, 1982-1983
U.S. Economic Assistance Program for the Republic of Zaire
Project Grant Agreement for USAID/ECZ Project (USAID Project 660-0086)
Draft Workplan, CCCD Project
Guidelines for CCCD Pre-Project Assessment
Surveillance Records, Kinshasa EPI
Hospital Registers, Mama Yemo, Kisantu and Ngidinga Hospitals, 1976-1982
Annual Report of USAID Consultant to PLAP, 1981

ANNEX 2

List of Technical Officer's Tasks

1. Assist with training of PHC personnel responsible for integration of EPI, CDD and (eventually) Malaria Control Activities.

    A. Assist with development of training materials.
    B. Serve as course facilitator for National and Regional Courses.
    C. Assist in development of technics for evaluation of training efficacy.

2. Assist in organization and implementation of National and International evaluations of project activities.

3. Assist with identification of indicators for measuring monthly program progress.

    A. Improve methods of monitoring selected indicators.
    B. Initiate feedback mechanism so that indicator data can be shared with participating health facilities.

4. Provide administrative and logistical support to regionally funded CCCD activities in Zaire.

    A. Provide follow-up for operational research activities initiated by Regional CCCD Staff.
    B. Provide follow-up for Health Information System Activities initiated by Regional CCCD Staff.

5. Assume responsibility for timely, appropriate and properly-utilized commodity support.

6. Cooperate with WHO in EPI and CDD activities as appropriate.

7. Assist Department of Public Health in development, implementation, evaluation and modification of program plans.

    A. Assist in development of specific vaccination coverage (as opposed to access) targets for EPI diseases.
    B. Assist in development of specific morbidity and mortality reduction targets for EPI activities.
    C. Cooperate with National, WHO and Regional CCCD personnel in development of access and coverage targets and mortality reduction targets for CDD.

8. Collaborate with responsible officials of the USAID/ECZ Project so that activities in PHC are complimentary and not competetive.

9. Assist National Officials to strengthen the Department of Public Health role in supervising and coordinating donor assistance to PHC activities.

ANNEX 3

Projected Expansion of PHC Activities in Zaire

The estimated number of health facilities which will be conducting Primary Health Care activities by the end of 1982 is 1,619.

By the end of the year 1986, it is estimated that there will be a total of 5,195 health facilities in the entire country. Since the National Health Plan projects that integrated PHC activities will be accessible to 60% of the total population by the end of 1986, an assumption is made that about 60% of the health facilities will be involved in integrated PHC activities by this date. This assumption means that approximately 3,000 health facilities will be offering integrated PHC activities by the end of 1986.

Based on an estimate of 1,600 health facilities offering integrated PHC activities by the end of 1982, and 3,000 by the end of 1986, the following estimate of health facilities offering integrated PHC activities for each year from 1982-1986 is made:

Year:	1982	1983	1984	1985	1986
Number:	1600	1950	2300	2650	3000

ANNEX 4

Projected Population Growth in Zaire

The present population of Zaire is estimated at 28,120,000. Assuming that the proportion of the population less than one year of age is 4.23%, the following estimates were made concerning the population increase and the numbers of children less than one year of age for the period 1982-1986.

	Total Population	Population Under One Year of Age
1982	28,120,000	1,190,000
1983	28,935,000	1,224,000
1984	29,775,000	1,260,000
1985	30,640,000	1,296,000
1986	31,525,000	1,334,000

An estimate of the growth of the urban population in Zaire can be obtained from 1975 and 1980 population figures for the 11 largest cities in Zaire.

	1975	1986
Kinshasa	1,944,256	2,410,552
Lubumbashi	551,975	700,000
Kananga	474,657	486,866
Kisangani	360,614	374,594
Mbuji-Mayi	283,219	335,195
Likasi	185,328	200,000
Bukavu	150,000	153,149
Kolwezi	143,287	160,000
Mbandaka	139,856	154,148
Kikwit	127,606	152,247
Matadi	93,963	167,710
