# Supplementary material for: Reaching Never- and Incompletely-Vaccinated Children with Routine Immunization: A Proof-of-Concept Activity Using Geo-Referenced Microplans in Two Health Zones in Maniema Province, Democratic Republic of the Congo
Source: Vaccines (Basel). 2026 Feb 13;14(2):175. doi: 10.3390/vaccines14020175 (PMC12945245; doi:10.3390/vaccines14020175)
Supplement: Supplementary file 1 [file vaccines-14-00175-s001.zip › Tools_Alleman_Maniema_10Feb2026.zip!/A1_Tool_S1A_Maniema_Enumeration_French.pdf]

Fiche de dénombrement des enfants de 0 à 23 mois et leur statut vaccinal

Projet de renforcement de la vaccination de routine chez des enfants non-vaccinés ou insuffisamment vaccinés à l'aide de micro plans validés dans les zones de santé à haut risque de transmission de la polio et de la rougeole

Kindu et Kibombo, Province du Maniema, République Démocratique du Congo, juillet 2022

AS____

Nom du block/village/avenue/campement_______________________________________

Numéro de fiche pour la journée___________

Nom RECO____

Date____________/______________/2022

01	02	03	04	05	06	07	08	09	10	11	12	13	14	15
N° Ménage	Nom, Post nom et Prénom de chaque enfant de 0 à 23 mois dans le ménage	Sexe enfant:<br>*Féminin=F*<br>*Masculin=M*	Code de l'age de l'enfant selon le guide d'age (code uniquement)<br>0<br>1 1/2<br>2 1/2<br>3 1/2<br>9	Existence carte de vaccination pour l'enfant:<br>*OUI=1*<br>*NON=0*	Enfant a une cicatrice BCG sur l'avant bras gauche (visible ou selon la mère)<br>OUI=1<br>NON=0	Enfant a été vacciné au moins une fois à l'hôpital ou un centre de santé:<br>*OUI=1*<br>*NON=0*	Raison principale enfant n'a jamais été vacciné à l'hôpital/centre de santé:<br>*a . pas au courant de l'avantage*<br>*b . site de vaccination trop éloigné ou aucun moyen de transport*<br>*c . longue attente au site de vaccination*<br>*d . peur des effets secondaires*<br>*e . manque de temps pour amener l'enfant*<br>*f. manque d'argent*<br>*g. réfractaire à la vaccination*<br>*h. refus*<br>*i. autre*<br>*j . enfant vacciné*	Est-ce que l'enfant a recu ses vaccins à partir de la la naissance<br>*Jamais été vacciné=2*<br>*OUI=1*<br>*NON=0*<br>*BCG*<br>*VPO0*	Est-ce que l'enfant a recu ses vaccins à partir de 1 mois et demi (6 semaines)<br>*Enfant trop jeune (moins de 1 mois et demi)=3*<br>*Jamais été vacciné=2*<br>*OUI=1*<br>*NON=0*<br>*VPO1*<br>*PENTA1*<br>*PCV1*<br>*ROTA1*	Est-ce que l'enfant a recu ses vaccins à partir de 2 mois et demi (10 semaines)<br>*Enfant trop jeune (moins de 2 mois et demi)=3*<br>*Jamais été vacciné=2*<br>*OUI=1*<br>*NON=0*<br>*VPO2*<br>*PENTA2*<br>*PCV2*<br>*ROTA2*	Est-ce que l'enfant a recu ses vaccins à partir de 3 mois et demi (14 semaines)<br>*Enfant trop jeune (moins de trois mois et demi)=3*<br>*Jamais été vacciné=2*<br>*OUI=1*<br>*NON=0*<br>*VPO3*<br>*PENTA3*<br>*PCV3*<br>*ROTA3 VPI*	Est-ce que l'enfant a recu ses vaccins à partir de 9 mois<br>*Enfant trop jeune (moins de 9 mois)=3*<br>*Jamais été vacciné=2*<br>*OUI=1*<br>*NON=0*<br>*VAR*<br>*VAA*	Est ce que l'enfant est complètement vacciné selon son âge?<br>Jamais été vacciné=2<br>*OUI=1*<br>*NON=0*	Raison principale enfant n'est pas complètement vacciné selon son âge:<br>*a . ne connaissait pas le calendrier ou a oublié le calendrier*<br>*b. site de vaccination trop éloigné ou aucun moyen de transport*<br>*c. longue attente au site de vaccination*<br>*d. peur des effets secondaires*<br>*e. manque de temps pour amener l'enfant*<br>*f. manque d'argent*<br>*g. réfractaire à la vaccination*<br>*h. refus*<br>*i. autre*<br>*j. Non applicable (enfant jamais vacciné ou complètement vacciné)*
	Total nombre d'enfants<br>_________________	Total F= __<br>Total M=__	Total 0 :_____<br>Total 1 et demi:_______<br>Total 2 et demi :______<br>Total 3 et demi:_______<br>Total 9 :______	Total 0=____<br>Total 1=____	Total 0=_____<br>Total 1=_____	Total 0=_____<br>Total 1=_____	Total a=_________<br>Total b=_________<br>Total c=_________<br>Total d=_________<br>Total e=_________<br>Total f=_________<br>Total g=________<br>Total h=________<br>Total i=_________<br>Total j=_________	Total 0=_____<br>Total 1=_____<br>Total 2=_____	Total 0=_______<br>Total 1=_______<br>Total 2=_______<br>Total 3=_______	Total 0=_______<br>Total 1=_______<br>Total 2=_______<br>Total 3=_______	Total 0=_______<br>Total 1=_______<br>Total 2=_______<br>Total 3=_______	Total 0=_______<br>Total 1=_______<br>Total 2=_______<br>Total 3=_______	Total 0=_______<br>Total 1=_______<br>Total 2=_______	Total a=_____<br>Total b=_____<br>Total c=_____<br>Total d=_____<br>Total e=_____<br>Total f=_____<br>Total g=_____<br>Total h=_____<br>Total i=_____<br>Total j=_____

Numéros de ménage dans cette fiche De: ____________ A:____________

Signature du RECO____
