# Supplementary material for: Reaching Never- and Incompletely-Vaccinated Children with Routine Immunization: A Proof-of-Concept Activity Using Geo-Referenced Microplans in Two Health Zones in Maniema Province, Democratic Republic of the Congo
Source: Vaccines (Basel). 2026 Feb 13;14(2):175. doi: 10.3390/vaccines14020175 (PMC12945245; doi:10.3390/vaccines14020175)
Supplement: Supplementary file 1 [file vaccines-14-00175-s001.zip › Tools_Alleman_Maniema_10Feb2026.zip!/A2_Tool_S1B_Maniema_Enumeration_Swahili.pdf]

Fiche de dénombrement des enfants de 0 à 23 mois et leur statut vaccinal
Projet de renforcement de la vaccination de routine chez des enfants non-vaccinés ou insuffisamment vaccinés à l'aide de micro plans validés dans les zones de santé à haut risque de transmission de la polio et de la rougeole
Kindu et Kibombo, Province du Maniema, République Démocratique du Congo, juillet 2022

AS____
Jina ya mungini/mukoba/mangene/musonge/Bloc____________________________________
Namba ya kikartasi ya siku________
Jina ya relais communautaire____
Tarehe_____________/______________/2022

01	02	03	04	05	06	07	08	09	10	11	12	13	14	15
N° Jamaha	Majina ya mtoto ndani ya nyumba tangu kuzalika mpaka miezi makumi mbili na tatu (0-23)	Mtoto :<br>*MwanaMuke=F*<br>*Mwana ume=M*	Code ya miezi ya kuzalika ya mtoto kufatana na guide d'age (jaza tu na chiffre)<br>0<br>$1^{1/2}$<br>$2^{1/2}$<br>$3^{1/2}$<br>9	Kartasi ya chanjo ya mtoto iko? :<br>Ndiyo=*1*<br>*Apana=0*	Mtoto iko na alama ya chanjo ya BCG ku mukono wa kushoto?<br>Ndiyo=*1*<br>*Apana=0*	Mtoto alipata chanjo ku hopitali au ku kituo cha afia mara moja?<br>*Ndiyo=1*<br>*Apana=0*	Sababu kuu gani ilituma mtoto kuto kupata chanjo ku hopitali au ku kituo cha afia?<br>*a. kuto kudjuwa faida ya chanjo*<br>*b. safari ndefu au kukosewa ginsi ya kufika ku hospitali au ku kituo cha afia*<br>*c. Kusubiri muda murefu*<br>*d. kuogopa vinyume ya chanjo*<br>*e. kukosa wa kati ya kupeleka mtoto*<br>*f. Kukosa makuta*<br>*g. bao banapingaka chanjo*<br>*h. bao banakatalaka chanjo*<br>*i. sababu ingine*<br>*j. Mutoto ali pata chanjo*	Mtoto alipata chanjo tangu kuzalika?<br>*Mtoto bado kupata chanjo=2*<br>*Ndiyo=1*<br>*Apana=0*<br>*BCG*<br>*VPO0*	Mtoto alipata chanjo tangu mwezi moja na nusu (juma sita)?<br>*Mtoto mudogo sana (chini ya mwezi moja na nusu)=3*<br>*Mtoto bado kupata chanjo =2*<br>*Ndiyo=1*<br>*Apana=0*<br>*VPO1*<br>*PENTA1*<br>*PCV1*<br>*ROTA1*	Mtoto alipata chanjo tangu miezi mbili na nusu (juma kumi)?<br>*Mtoto mudogo sana (chini ya miezi bili na nusu)=3*<br>*Mtoto bado kupata chanjo =2*<br>*Ndiyo=1*<br>*Apana=0*<br>*VPO2*<br>*PENTA2*<br>*PCV2*<br>*ROTA2*	Mtoto alipata chanjo tangu miezi tatu na nusu (juma kumi na ine)?<br>*Mtoto mudogo sana (chini ya miezi tatu na nusu)=3*<br>*Mtoto bado kupata chanjo=2*<br>*Ndiyo=1*<br>*Apana=0*<br>*VPO3*<br>*PENTA3*<br>*PCV3*<br>*ROTA3 VPI*	Mtoto alipata chanjo tangu miezi kenda?<br>*Mtoto mudogo sana (chini ya miezi kenda)=3*<br>*Mtoto bado kupata chanjo=2*<br>*Ndiyo=1*<br>*Apana=0*<br>*VAR VAA*	Mtoto amekuisha kupata chanjo yote kufatana na miaka yake ya kuzalika?<br>*Mtoto bado kupata chanjo =2*<br>*Ndiyo=1*<br>*Apana=0*	Sababu kuu gani inatuma mtoto aku pata chanjo yake yote kufatana na miaka yake ya kuzalika?<br>*a. kuto kudjua kalendari ya chanjo au kusahawu kalendari ya chanjo*<br>b. safari ndefu au kukosewa ginsi ya kufika ku hopitali au ku kituo cha afia<br>c. kusubiri muda murefu<br>d. kuogopa vinyume ya chanjo<br>e. kukosa wa kati ya kupeleka mtoto<br>f. kukosa makuta<br>g. bao banapingaka chanjo<br>h. bao banakatalaka chanjo<br>i. sababu ingine<br>*j. hakuna sababu (Mtoto bado kupata chanjo ao alisha pata chanjo yote)*
	Esabu ya watoto wote<br>____________	Esabu ya wanawake (F)= __<br>Esabu ya wanawume (M)=__	Esabu ya watoto wa code zero :______<br>Esabu ya watoto wa code moja na nusu:______<br>Esabu ya watoto wa code mbili na nusu:______<br>Esabu ya watoto wa code tatu na nusu:______<br>Esabu ya watoto wa code kenda :______	Esabu 0=____<br>Esabu 1=____	Esabu 0=___<br>Esabu 1=___	Esabu 0=_____<br>Esabu 1=_____	Esabu a=_________<br>Esabu b=_________<br>Esabu c=_________<br>Esabu d=_________<br>Esabu e=_________<br>Esabu f=_________<br>Esabu g=_________<br>Esabu h=_________<br>Esabu i=_________<br>Esabu j=_________	Esabu 0=_____<br>Esabu 1=_____<br>Esabu 2=_____	Esabu 0=_______<br>Esabu 1=_______<br>Esabu 2=_______<br>Esabu 3=_______	Esabu 0=_______<br>Esabu 1=_______<br>Esabu 2=_______<br>Esabu 3=_______	Esabu 0=_______<br>Esabu 1=_______<br>Esabu 2=_______<br>Esabu 3=_______	Esabu 0=_______<br>Esabu 1=_______<br>Esabu 2=_______<br>Esabu 3=_______	Esabu 0=_______<br>Esabu 1=_______<br>Esabu 2=_______	Esabu a=_____<br>Esabu b=_____<br>Esabu c=_____<br>Esabu d=_____<br>Esabu e=_____<br>Esabu f=_____<br>Esabu g=_____<br>Esabu h=_____<br>Esabu i=_____<br>Esabu j=_____

Namba ya nyumba (jamaa) De:____________ A:____________
Signature du RECO____
