# Supplementary material for: Reaching Never- and Incompletely-Vaccinated Children with Routine Immunization: A Proof-of-Concept Activity Using Geo-Referenced Microplans in Two Health Zones in Maniema Province, Democratic Republic of the Congo
Source: Vaccines (Basel). 2026 Feb 13;14(2):175. doi: 10.3390/vaccines14020175 (PMC12945245; doi:10.3390/vaccines14020175)
Supplement: Supplementary file 1 [file vaccines-14-00175-s001.zip › Tools_Alleman_Maniema_10Feb2026.zip!/A3_Tool_S1C_Maniema_Enumeration_English.pdf]

Enumeration form for children aged 0 - 23 months with their vaccination status

Project to reinforce routine vaccination among children never vaccinated or incompletely vaccinated using validated microplans in health zones at high risk for transmission of measles and polio

Kindu and Kibombo, Maniema Province, Democratic Republic of the Congo, July 2022

Name of Health Area____

Name of block/village/avenue/camp______________________________________

Number of form for the day____________

Name of community volunteer____

Date_____________/_______________/2022

01	02	03	04	05	06	07	08	09	10	11	12	13	14	15
No. House	Last name and first name of each children aged 0 - 23 months in the house	Sex of the child<br>*Female=F*<br>*Male=M*	Code for the child's age according to the age guide (enter code only)<br>0<br>1 1/2<br>2 1/2<br>3 1/2<br>9	Does the child have a vaccination card?<br>*YES=1*<br>*NO=0*	Does the child have a BCG scar on the left forearm (visible or according to the mother?)<br>OUI=1<br>NON=0	Has the child been vaccinated at least one time at a hospital or health center?<br>*YES=1*<br>*NO=0*	Principal reason the child was never vaccinated at a hospital or health center?<br>*a . not aware of the benefits*<br>*b . vaccination site is too far or no means of transport*<br>*c . long waits at the vaccination site*<br>*d . fear of adverse events*<br>*e . lack of time to bring the child*<br>*f. lack of money*<br>*g. organized refusal to vaccinate*<br>*h. individual refusal to vaccinate*<br>*i. other*<br>*j . child has been vaccinated*	Did the child receive the recommended birth dose vaccines?<br>*Child has never been vaccinated=2*<br>*YES=1*<br>*NO=0*<br>*BCG*<br>*OPV0*	Did the child receive the vaccines recommended as of one month and a half (6 weeks)<br>*Child is too young (less than one and a half months)=3*<br>*Child has never been vaccinated=2*<br>*YES=1*<br>*NO=0*<br>*OPV1*<br>*PENTA1*<br>*PCV1*<br>*ROTA1*	Did the child receive the vaccines recommended as of two and a half months (10 weeks)<br>*Child is too young (less than two and one half months)=3*<br>*Child has never been vaccinated=2*<br>*YES=1*<br>*NO=0*<br>*OPV2*<br>*PENTA2*<br>*PCV2*<br>*ROTA2*	Did the child receive the vaccines recommended as of three and a half months (14 weeks)<br>*Child is too young (less than three and one half months)=3*<br>*Child has never been vaccinated=2*<br>*YES=1*<br>*NO=0*<br>*OPV3*<br>*PENTA3*<br>*PCV3*<br>*ROTA3 IPV*	Did the child receive the vaccines recommended as of nine months?<br>*Child is too young (less than nine months)=3*<br>*Child has never been vaccinated=2*<br>*YES=1*<br>*NO=0*<br>*Yellow Fever*<br>*Measles*	Is the child completely vacicnated according to their age?<br>Child has never been vaccinated=*2*<br>*YES=1*<br>*NO=0*	Principal reason the child was not completely vaccinated according to their age?<br>*a . do not know, or have forgotten, the vaccination schedule*<br>*b. vaccination site is too far or no means of transport*<br>*c. long waits at the vaccination site*<br>*d. fear of adverse events*<br>*e. lack of time to bring the child*<br>*f. lack of money*<br>*g. organized refusal to vaccinate*<br>*h. individual refusal to vaccinate*<br>*i. other*<br>*j. Non applicable (child has never been vaccinated or is completely vaccinated according to their age)*
	Total number of children<br>__________________	Total F= __<br>Total M=__	Total 0 :_____<br>Total 1 and a half:_______<br>Total 2 and a half :______<br>Total 3 and a half:_______<br>Total 9 :______	Total 0=____<br>Total 1=____	Total 0=_____<br>Total 1=_____	Total 0=_____<br>Total 1=_____	Total a=_________<br>Total b=_________<br>Total c=_________<br>Total d=_________<br>Total e=_________<br>Total f=_________<br>Total g=________<br>Total h=________<br>Total i=_________<br>Total j=_________	Total 0=_____<br>Total 1=_____<br>Total 2=_____	Total 0=_______<br>Total 1=_______<br>Total 2=_______<br>Total 3=_______	Total 0=_______<br>Total 1=_______<br>Total 2=_______<br>Total 3=_______	Total 0=_______<br>Total 1=_______<br>Total 2=_______<br>Total 3=_______	Total 0=_______<br>Total 1=_______<br>Total 2=_______<br>Total 3=_______	Total 0=_______<br>Total 1=_______<br>Total 2=_______	Total a=_____<br>Total b=_____<br>Total c=_____<br>Total d=_____<br>Total e=_____<br>Total f=_____<br>Total g=_____<br>Total h=_____<br>Total i=_____<br>Total j=_____

Number of houses recorded on this form From: _____________ To:_____________

Signature of community volunteer____
