# Supplementary material for: Reaching Never- and Incompletely-Vaccinated Children with Routine Immunization: A Proof-of-Concept Activity Using Geo-Referenced Microplans in Two Health Zones in Maniema Province, Democratic Republic of the Congo
Source: Vaccines (Basel). 2026 Feb 13;14(2):175. doi: 10.3390/vaccines14020175 (PMC12945245; doi:10.3390/vaccines14020175)
Supplement: Supplementary file 1 [file vaccines-14-00175-s001.zip › Tools_Alleman_Maniema_10Feb2026.zip!/B1_Tool_S2A_Maniema_AgeGuide_French.pdf]

Guide d'âge 0-23 mois pour le PEV systématique Juillet 2022

Mois / Année	Janvier	Février	Mars	Avril	Mai	Juin	Juillet	Aout	Septembre	Octobre	Novembre	Décembre
2020								23	22	21	20	19
								9				

Mois / Année	Janvier	Février	Mars	Avril	Mai	Juin	Juillet	Aout	Septembre	Octobre	Novembre	Décembre
2021	18	17	16	15	14	13	12	11	10	9	8	7
	9										$3\frac{1}{2}$	

Mois / Année	Janvier	Février	Mars	Avril	Mai	Juin	Juillet	Aout	Septembre	Octobre	Novembre	Décembre
2022	6	5	4	3	2	1	0					
	$3\frac{1}{2}$			$2\frac{1}{2}$	$1\frac{1}{2}$	0	0					

TABLEAU DE SYNTHESE

**TRANCHE D'AGE DE L'ENFANT**	**CODE A REMPLIR**
De 0 mois à 1 mois sur le guide d'âge<br>Mtoto iko na zero ao mwezi moja	0
2 mois sur le guide d'âge<br>Mtoto iko na miezi mbili ku guide d'âge	$1\frac{1}{2}$
3 mois sur le guide d'âge<br>Mtoto iko miezi tatu ku guide d'age	$2\frac{1}{2}$
De 4 mois à 8 mois sur le guide d'âge<br>Kutoka miezi ine mpaka mnane ku guide d'âge	$3\frac{1}{2}$
De 9 mois à 23 mois sur le guide d'âge<br>Kutoka miezi kenda mpaka makumi bili na tatu ku guide d'âge	9
