# Supplementary material for: Reaching Never- and Incompletely-Vaccinated Children with Routine Immunization: A Proof-of-Concept Activity Using Geo-Referenced Microplans in Two Health Zones in Maniema Province, Democratic Republic of the Congo
Source: Vaccines (Basel). 2026 Feb 13;14(2):175. doi: 10.3390/vaccines14020175 (PMC12945245; doi:10.3390/vaccines14020175)
Supplement: Supplementary file 1 [file vaccines-14-00175-s001.zip › Tools_Alleman_Maniema_10Feb2026.zip!/B2_Tool_S2B_Maniema_AgeGuide_English.pdf]

Age Guide for routine immunization for children aged 0-23 months, July 2022

Month / Year	JAN	FEB	MAR	APR	MAY	JUN	JUL	AUG	SEP	OCT	NOV	DEC
2020								23	22	21	20	19
								9				

Month / Year	JAN	FEB	MAR	APR	MAY	JUN	JUL	AUG	SEP	OCT	NOV	DEC
2021	18	17	16	15	14	13	12	11	10	9	8	7
	9										$3\frac{1}{2}$	

Month / Year	JAN	FEB	MAR	APR	MAY	JUN	JUL	AUG	SEP	OCT	NOV	DEC
2022	6	5	4	3	2	1	0					
	$3\frac{1}{2}$			$2\frac{1}{2}$	$1\frac{1}{2}$	0	0					

Abbreviations: JAN, January; FEB, February; MAR, March; APR, April; JUN, June; JUL, July, AUG, August; SEP, September; OCT, October; NOV, November; DEC, December
