# Supplementary material for: Reaching Never- and Incompletely-Vaccinated Children with Routine Immunization: A Proof-of-Concept Activity Using Geo-Referenced Microplans in Two Health Zones in Maniema Province, Democratic Republic of the Congo
Source: Vaccines (Basel). 2026 Feb 13;14(2):175. doi: 10.3390/vaccines14020175 (PMC12945245; doi:10.3390/vaccines14020175)
Supplement: Supplementary file 1 [file vaccines-14-00175-s001.zip › Tools_Alleman_Maniema_10Feb2026.zip!/C1_Tool_S3A_Maniema_RoutineImmunizationSchedule_Graphic_French.pdf]

Calendrier Vaccinal des Enfants en RDC
(S'il y a une carte de vaccination, s'y référer)

À partir de 0 mois
(dès la naissance)

Les vaccins protègent contre :
VPO0 : poliomyélite (magonjwa ya vunja vunja ao kupooza)
BCG : tuberculose (magonjwa ya kifuwa ki kuu, tibe)

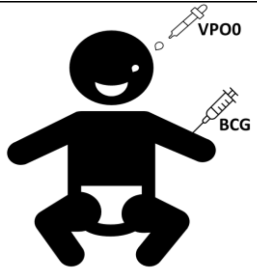

À partir de 1½ mois
(6 semaines)

Les vaccins protègent contre :
VPO1 : poliomyélite (magonjwa ya vunja vunja ao kupooza)
ROTA1 : diarrhées (magonjwa ya kuarisha)
PCV13(1) : pneumonie (fieke)
PENTA1 : diphtérie (kamondo), tétanos (pepo punda), coqueluche (magonjwa jogoo), hépatite virale B, méningite

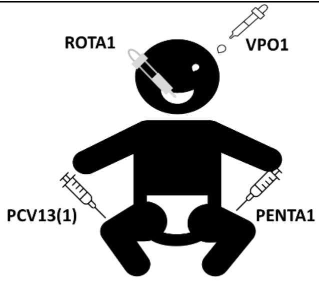

À partir de 2½ mois
(10 semaines)

Les vaccins protègent contre :
VPO2 : poliomyélite (magonjwa ya vunja vunja ao kupooza)
ROTA2 : diarrhées (magonjwa ya kuarisha)
PCV13(2) : pneumonie (fieke)
PENTA2 : diphtérie (kamondo), tétanos (pepo punda), coqueluche (magonjwa jogoo), hépatite virale B, méningite

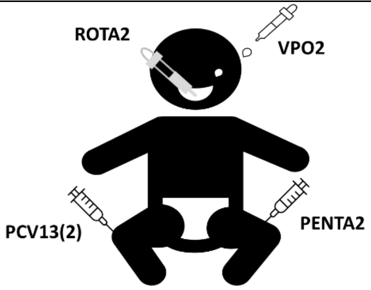

À partir de 3½ mois
(14 semaines)

Les vaccins protègent contre :
VPO3 et VPI(1) : poliomyélite (magonjwa ya vunja vunja ao kupooza)
ROTA3 : diarrhées (magonjwa ya kuarisha)
PCV13(3) : pneumonie (fieke)
PENTA3 : diphtérie (kamondo), tétanos (pepo punda), coqueluche (magonjwa jogoo), hépatite virale B, méningite

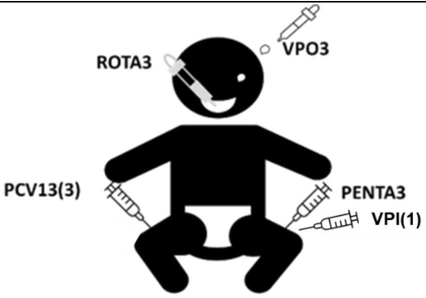

À partir de 9 mois

Les vaccins protègent contre :
VAA : Fièvre jaune (manjano)
VAR1 : Rougeole (magonjwa ya suluba)
VPI(2)* : poliomyélite (magonjwa ya vunja vunja ao kupooza)

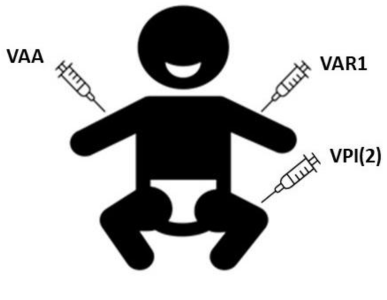

À partir de 15 mois

Le vaccin protège contre :
VAR2* : Rougeole (magonjwa ya suluba)

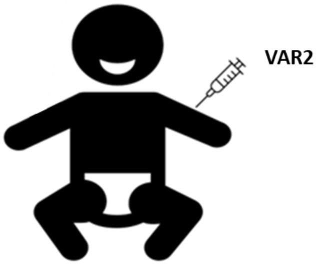

***En 2023, la RDC a introduit une deuxième dose de vaccin antipoliomyélitique inactivé (VPI) à l'âge de 9 mois et une deuxième dose de vaccin contre la rougeole à l'âge de 15 mois.**
