# Supplementary material for: Reaching Never- and Incompletely-Vaccinated Children with Routine Immunization: A Proof-of-Concept Activity Using Geo-Referenced Microplans in Two Health Zones in Maniema Province, Democratic Republic of the Congo
Source: Vaccines (Basel). 2026 Feb 13;14(2):175. doi: 10.3390/vaccines14020175 (PMC12945245; doi:10.3390/vaccines14020175)
Supplement: Supplementary file 1 [file vaccines-14-00175-s001.zip › Tools_Alleman_Maniema_10Feb2026.zip!/C2_Tool_S3B_ Maniema_RoutineImmunizationSchediule_Graphic_Englsh.pdf]

Vaccination Schedule for Children in DRC
(If there is a vaccination card for the child, use the information on the card)

<table>
<tr>
<td>

As of 0 months of age
(birth dose)

These vaccines protect against:
OPV0: polio
BCG: tuberculosis

</td>
<td>
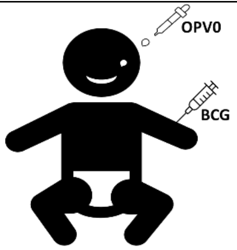

</td>
</tr>
<tr>
<td>

As of 1½ months of age
(6 weeks)

These vaccines protect against:
OPV1: polio
ROTA1: diarrhea
PCV13(1): pneumonia
PENTA1: diphtheria, tetanus, pertussis, viral hepatitis B, meningitis

</td>
<td>
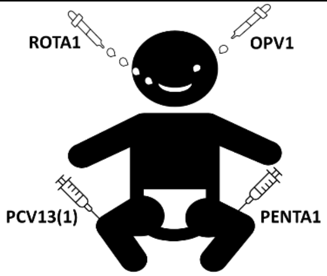

</td>
</tr>
<tr>
<td>

As of 2½ months of age
(10 weeks)

These vaccines protect against:
OPV2: polio
ROTA2: diarrhea
PCV13(2): pneumonia
PENTA2: diphtheria, tetanus, pertussis, viral hepatitis B, meningitis

</td>
<td>
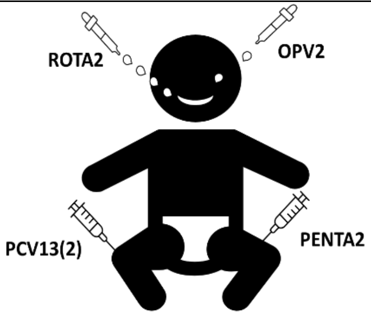

</td>
</tr>
<tr>
<td>

As of 3½ months of age
(14 weeks)

These vaccines protect against:
OPV3 and IPV(1): polio
ROTA2: diarrhea
PCV13(2): pneumonia
PENTA2: diphtheria, tetanus, pertussis, viral hepatitis B, meningitis

</td>
<td>
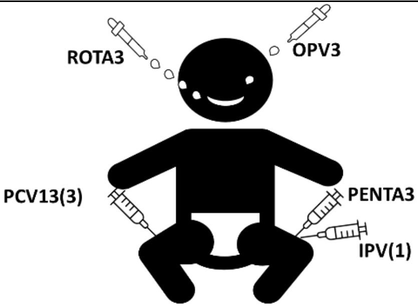

</td>
</tr>
<tr>
<td>

As of 9 months of age

These vaccines protect against:
IPV(2)*: polio
YF: yellow fever
MCV(1): measles (containing vaccine)

</td>
<td>
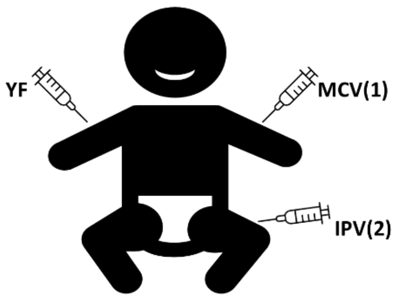

</td>
</tr>
<tr>
<td>

As of 15 months of age

This vaccine protects against:
MCV(2)*: measles (containing vaccine)

</td>
<td>
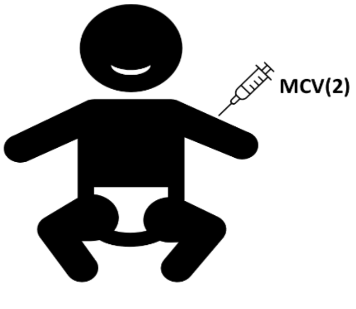

</td>
</tr>
</table>

*In 2023, DRC introduced a second dose of inactivated polio vaccine (IPV) at 9 months and a second dose of measles vaccine at 15 months.
