# Supplementary material for: Reaching Never- and Incompletely-Vaccinated Children with Routine Immunization: A Proof-of-Concept Activity Using Geo-Referenced Microplans in Two Health Zones in Maniema Province, Democratic Republic of the Congo
Source: Vaccines (Basel). 2026 Feb 13;14(2):175. doi: 10.3390/vaccines14020175 (PMC12945245; doi:10.3390/vaccines14020175)
Supplement: Supplementary file 1 [file vaccines-14-00175-s001.zip › Tools_Alleman_Maniema_10Feb2026.zip!/D1_Tool_S4A_Maniema_EnumerationGuide_French.pdf]

Project de Renforcement de la Vaccination de Routine, Phase cartographie : Kindu et Kibombo, MANIEMA
Guide : Formulaire de dénombrement des enfants de l'âge de 0-23 mois et leur statut vaccinal, Version 10 juillet 2022

N°	Question	Valeurs (ligne total)	Validation	Commentaire
Q01	N° Ménage<br><br><u>Pour ce projet, un ménage est composé des enfants qui sont sous la tutelle de même adulte.</u>	Les numéros de ménage dans la fiche sont notés en bas de chaque fiche :<br>De : ________<br>A : _________		• Le numéro de ménage recommence á 01 chaque jour pour chaque RECO.<br>• Le numéro de ménage est le même pour tous les enfants qui habitent dans ce ménage.<br>• Le dernier chiffre correspond au dernier ménage sur la fiche. Le premier et le dernier numéro de ménage dans chaque fiche sont notés en bas de la fiche.<br>• Au cours de la même journée, si l'enregistrement des enfants d'un ménage nécessite des lignes supplémentaires sur une autre fiche, la numérotation des ménages continue sur la fiche suivante.
Q02	Nom, Post Nom et Prénom de chaque enfant de 0 à 23 mois dans le ménage	Total nombre d'enfants	Inférieur ou égal au nombre de lignes sur la fiche	• Chaque fiche a 24 lignes.<br>• Si la famille ne veut pas donner le nom de l'enfant, il faut donner un nom générique comme « enfant 1 », « enfant 2 », « bébé 1 », « bébé 2 », etc.
Q03	Sexe enfant [féminin (F) ou masculin (M)]	Total F=<br>Total M=	Total F + Total M = Total nombre d'enfants (Q02)	
Q04	Code de l'âge de l'enfant (selon le guide d'âge, code uniquement)	Total code 0 =<br>Total code 1 ½ =<br>Total code 2 ½ =<br>Total code 3 ½ =<br>Total code 9 =	Total 0 + Total 1 ½ +<br>Total 2 ½ +<br>Total 3 ½ +<br>Total 9 =Total nombre d'enfants (Q02)	• Il faut suivre le guide d'âge.<br><br>• La réponse est le code uniquement.
Q05	Existence carte de vaccination pour l'enfant	Total 0=<br>Total 1=	Total 0 + Total 1 = Total nombre d'enfants (Q02)	• Si la carte est disponible ou la gardienne confirme qu'il y en a/avait (mais pas disponible, perdue, ou avec une autre personne, etc.), la ligne correspondante pour Q05 devient 1 « Oui ».<br>• Si l'enfant n'a jamais eu une carte de vaccination ou la gardienne ne sait pas, la ligne correspondante pour Q05 devient 0 « Non ».<br>• La carte de vaccination est une preuve pour les vaccins reçus par l'enfant. Si l'enfant a une carte de vaccination, l'information sur la carte doit être utilisée par le RECO pour remplir les questions relatives aux vaccins.

Q06	**Enfant a une cicatrice BCG sur l'avant-bras gauche**	**Total 0=**<br>**Total 1=**	**Total 0 + Total 1 = Total nombre d'enfants (Q02)**	• **Si l'enfant a une cicatrice sur l'avant-bras gauche, c'est une preuve que l'enfant a été vacciné au moins une fois et une preuve pour les vaccins reçus à partir de 0 mois (dès la naissance, BCG/VPO0). La ligne correspondante pour Q06 devient 1 « Oui » et celle pour Q07 devient 1 « Oui » aussi.**<br>• **Si l'enfant est absent et la gardienne dit que l'enfant a une cicatrice, la ligne correspondante pour Q06 devient 1 « Oui » est celle pour Q07 devient 1 « Oui » aussi.**<br>• **Si l'enfant n'a pas une cicatrice sur l'avant-bras gauche ou si l'enfant est absent et la gardienne dit que l'enfant n'a pas une cicatrice ou la gardienne ne sait pas, la ligne correspondante pour Q06 devient 0 « Non ».**
Q07	**Enfant a été vacciné au moins une fois à l'hôpital/centre de santé**	**Total 0=**<br>**Total 1=**	**Total 0 + Total 1 = Total nombre d'enfants (Q02)**<br><br>**Total 0 = non-vaccinés (Jamais été vacciné)**	• **Les enfants qui n'ont jamais été vacciné à l'hôpital/centre de santé sont les enfants non-vaccinés. La ligne correspondante pour Q07 devient 0 « Non ». On ne tient pas compte des campagnes de vaccination.**<br><br>• **Si la ligne pour Q07 est 0 (enfant jamais été vacciné), alors :**<br>✓ **La ligne correspondante pour Q09 devient 2 : « Jamais été vacciné »**<br>✓ **La ligne correspondante pour Q10 devient 2 : « Jamais été vacciné »**<br>✓ **La ligne correspondante pour Q11 devient 2 : « Jamais été vacciné »**<br>✓ **La ligne correspondante pour Q12 devient 2 : « Jamais été vacciné »**<br>✓ **La ligne correspondante pour Q13 devient 2 : « Jamais été vacciné »**<br>✓ **La ligne correspondante pour Q14 devient 2 : « Jamais été vacciné »**<br>✓ **La ligne correspondante pour Q15 devient j : Non applicable (« Jamais été vacciné ou complètement vacciné »)**<br><br>****Si l'enfant a une cicatrice sur l'avant-bras gauche, c'est une preuve que l'enfant a été vacciné au moins une fois et une preuve pour les vaccins reçus à partir de 0 mois (dès la naissance, BCG/VPO0) ou si la carte de vaccination montre que l'enfant a reçu au moins un vaccin, l'information sur la carte doit être utilisée par le RECO pour remplir la question. Le réponse á Q07 devient 1.****

Q08	Raison enfant n'a jamais été vacciné à l'hôpital/centre de santé	Total a=<br>Total b=<br>Total c=<br>Total d=<br>Total e=<br>Total f=<br>Total g=<br>Total h=<br>Total i=<br>Total j=	Total a + Total b + Total c + Total d + Total e + Total f + Total g + Total h + Total i +Total j = Total nombre (Q02) d'enfants<br><br>Total j « enfant vacciné » = Total 1 « Oui » (Q07)	• Pour commencer, « enfant vacciné » (j) : aux enfants qui ont été vacciné au moins une fois à l'hôpital/centre de santé.
Q09	Vaccins reçus à partir de la naissance (BCG/VPO0)	Total 0=<br>Total 1=<br>Total 2=	Total 0 + Total 1 + Total 2=Total nombre d'enfants (Q02)<br><br>Total 2 « Jamais été vacciné » = Total 0 "Non" (Q07)	• Pour commencer, la réponse pour les enfants qui n'ont jamais été vaccines est (2) (Voir ci-dessus, Q07).<br>• Ensuite, si l'enfant a reçu le BCG ou le VPO0, la réponse est 1 « Oui ». Sinon, la réponse est 0 « Non ».<br><br>• Les RECO devront parfois modifier l'information sur la fiche. Par exemple :<br>✓ L'enfant à l'âge de 0 mois (Q04)<br>✓ L'enfant n'a pas de carte de vaccination (Q05=0)<br>✓ L'enfant n'a pas de cicatrice sur l'avant-bras gauche (Q06=0)<br>✓ La gardienne interrogée dit que l'enfant a déjà été vaccinée (Q07=1)<br>✓ mais la gardienne interrogée dit que l'enfant n'a pas reçu de BCG ou VPO0 (Q09 = 0)<br>✓ alors le RECO doit entrer l'information de la façon suivante :<br>✓ La ligne correspondante pour Q07 devient 0 : L'enfant à l'âge de 0 mois est considéré non-vacciné<br>✓ La ligne correspondante pour Q09 devient 2 : « Jamais été vacciné »<br>✓ La ligne correspondante pour Q10 devient 2 : « Jamais été vacciné »<br>✓ La ligne correspondante pour Q11 devient 2 : « Jamais été vacciné »<br>✓ La ligne correspondante pour Q12 devient 2 : « Jamais été vacciné »<br>✓ La ligne correspondante pour Q13 devient 2 : « Jamais été vacciné »<br>✓ La ligne correspondante pour Q14 devient 2 : « Jamais été vacciné »<br>✓ La ligne correspondante pour Q15 devient j : Non applicable (« Jamais été vacciné ou complètement vacciné »)

Q10	Vaccins reçus à partir de 1½ mois (un mois et demi)<br>(6 semaines)<br><br>(VPO1/PENTA1/PCV13(1)/ROTA1)	Total 0=<br>Total 1=<br>Total 2=<br>Total 3=	Total 0 + Total 1 + Total 2 + Total 3 =Total nombre d'enfants (Q02)<br>Total 3 « Enfant trop jeune » + Total 2 « jamais été vacciné » > = Total 0 "Non" (Q07)	• Pour commencer, « Jamais été vacciné » (2) : aux enfants qui n'ont jamais été vaccines (Voir ci-dessus, Q07).<br>• Ensuite, « Enfant trop jeune » (3) : aux enfants trop jeunes pour recevoir ces vaccins.<br>• Enfin, si l'enfant a reçu le VPO1 ou le PENTA1 ou le PCV13(1) ou le ROTA1, la réponse est 1 « Oui ». Sinon, la réponse est 0 « Non ».<br>• Le relais communautaire doit comparer l'âge de l'enfant avec l'âge minimum pour recevoir ces vaccins.
Q11	Vaccins reçus à partir de 2½ mois (2 mois et demi)<br>(10 semaines)<br><br>(VPO2/PENTA2/PCV13(2)/ROTA2)	Total 0=<br>Total 1=<br>Total 2=<br>Total 3=	Total 0 + Total 1 + Total 2 + Total 3 =Total nombre d'enfants (Q02)<br>Total 3 « Enfant trop jeune » + Total 2 « jamais été vacciné » > = Total 0 "Non" (Q07)	• Pour commencer, « Jamais été vacciné » (2) : aux enfants qui n'ont jamais été vaccines (Voir ci-dessus, Q07).<br>• Ensuite, « Enfant trop jeune » (3) : aux enfants trop jeunes pour recevoir ces vaccins.<br>• Enfin, si l'enfant a reçu le VPO2 ou le PENTA2 ou le PCV13(2) ou le ROTA2, la réponse est 1 « Oui ». Sinon, la réponse est 0 « Non ».<br>• Le relais communautaire doit comparer l'âge de l'enfant avec l'âge minimum pour recevoir ces vaccins.
Q12	Vaccins reçus à partir de 3 ½ mois (3 mois et demi)<br>(14 semaines)<br><br>(VPO3/PENTA3/PCV13(3)/ROTA3/ VPI)	Total 0=<br>Total 1=<br>Total 2=<br>Total 3=	Total 0 + Total 1 + Total 2 + Total 3 =Total nombre d'enfants (Q02)<br>Total 3 « Enfant trop jeune » + Total 2 « jamais été vacciné » > = Total 0 "Non" (Q07)	• Pour commencer, « Jamais été vacciné » (2) : aux enfants qui n'ont jamais été vaccines (Voir ci-dessus, Q07).<br>• Ensuite, « Enfant trop jeune » (3) : aux enfants trop jeunes pour recevoir ces vaccins.<br>• Enfin, si l'enfant a reçu le VPO3 ou le PENTA3 ou le PCV13(3) ou le ROTA3 ou le VPI, la réponse est 1 « Oui ». Sinon, la réponse est 0 « Non ».<br>• Le relais communautaire doit comparer l'âge de l'enfant avec l'âge minimum pour recevoir les vaccins
Q13	Vaccin reçus à partir de 9 mois<br><br>(VAR/VAA)	Total 0=<br>Total 1=<br>Total 2=<br>Total 3=	Total 0 + Total 1 + Total 2 + Total 3 =Total nombre d'enfants (Q02)<br>Total 3 « Enfant trop jeune » + Total 2 « jamais été vacciné » > = Total 0 "Non" (Q07)	• Pour commencer, « Jamais été vacciné » (2) : aux enfants qui n'ont jamais été vaccines (Voir ci-dessus, Q07).<br>• Ensuite, « Enfant trop jeune » (3) : aux enfants trop jeunes pour recevoir ces vaccins.<br>• Enfin, si l'enfant a reçu le VAA ou le VAR, la réponse est 1 « Oui ». Sinon, la réponse est 0 « Non ».<br>• Le relais communautaire doit comparer l'âge de l'enfant avec l'âge minimum pour recevoir les vaccins

Q14	**Enfant complètement vacciné selon son âge**	**Total 0=<br>Total 1=<br>Total 2=**	**Total 0 + Total 1 + Total 2=Total nombre d'enfants (Q02)<br><br>Total 2 « Jamais été vacciné » = Total 0 "Non" (Q07)**	**• Règles pour remplir cette colonne :<br>✓ Pour commencer, s'il n'y a que des 2 pour les questions Q09, Q10, Q11, Q12, Q13 alors la ligne correspondante pour Q14 devient 2 « jamais été vacciné » (Voir ci-dessus, Q07).<br>✓ S'il y'a au moins un zéro pour les questions Q09, Q10, Q11, Q12, Q13 alors la ligne correspondante pour Q14 devient 0 : « enfant n'est pas complètement vacciné selon son âge ».<br>✓ S'il y'a des 1 et des 3 (pas de zéro ou 2) pour les questions Q09, Q10, Q11, Q12, Q13 alors la ligne correspondante pour Q14 devient 1 : « Enfant complètement vacciné selon son âge ».<br>• Le relais communautaire doit passer en revue l'âge de l'enfant avec l'âge minimum pour recevoir les vaccins.**
Q15	**Raison enfant n'est pas complètement vacciné selon son âge**	**Total a=<br>Total b=<br>Total c=<br>Total d=<br>Total e=<br>Total f=<br>Total g=<br>Total h=<br>Total i=<br>Total j=**	**Total j « jamais été vacciné ou complètement vacciné » >= Total 0 "Non" (Q07)<br>Total j « jamais été vacciné ou complètement vaccin » = Total 1 « Oui » (Q14) + Total 0 « Non » (Q07)**	**• j « Non applicable (jamais été vacciné ou complètement vacciné) »: aux enfants qui n'ont jamais été vaccines (Voir ci-dessus, Q07).<br>• j « Non applicable (jamais été vacciné ou complètement vacciné) »: aux enfants complètement vacciné selon leur âge.**
