# Supplementary material for: Reaching Never- and Incompletely-Vaccinated Children with Routine Immunization: A Proof-of-Concept Activity Using Geo-Referenced Microplans in Two Health Zones in Maniema Province, Democratic Republic of the Congo
Source: Vaccines (Basel). 2026 Feb 13;14(2):175. doi: 10.3390/vaccines14020175 (PMC12945245; doi:10.3390/vaccines14020175)
Supplement: Supplementary file 1 [file vaccines-14-00175-s001.zip › Tools_Alleman_Maniema_10Feb2026.zip!/D2_Tool_S4B_Maniema_EnumerationGuide_English.pdf]

Project to reinforce routine vaccination, participatory mapping phase: Kindu et Kibombo, Maniema Province, DRC
Guide: Enumeration questionnaire for children aged 0 - 23 months and their vaccination status, July 2022

No.	Question	Value (total)	Validation	Commentaire
Q01	No. of household<br><br><u>For this project, a household is composed of children under the care of the same adult.</u>	The numbering of households on a particular sheet of the questionnaire are noted at the bottom of the questionnaire:<br>From: ________<br>To: _________		• The household numbering restarts each day with 01 for each community volunteer.<br>• The numbering of a household is the same for all infants who live in that household.<br>• The last number on each enumeration questionnaire corresponds to the last household with a child recorded on the sheet. The first and the last household numbers included on a particular sheet of the questionnaire are noted at the bottom of the questionnaire.<br>• Over the course of the same day, if the data entries for the children in the same household require additional rows on a subsequent questionnaire, the numbering of that particular household continues on that next questionnaire and then subsequent households can be added.
Q02	Last and first name of each child aged 0 – 23 months in the household.	Total number of children	Less than or equal to the number of rows on the questionnaire.	• Each questionnaire has 24 rows.<br>• If the family does not wish to give the child's name, please assign a generic name to the child, such as "child 1", "baby 1", "baby 2", etc.
Q03	Sex of the child [female (F) or male (M)]	Total F=<br>Total M=	Total F + Total M = Total number of children (Q02)	
Q04	Code for the age of the child (according to the age guide, only note the appropriate code, not the child's age)	Total code 0 =<br>Total code 1 ½ =<br>Total code 2 ½ =<br>Total code 3 ½ =<br>Total code 9 =	Total 0 + Total 1 ½ +<br>Total 2 ½ +<br>Total 3 ½ +<br>Total 9 =Total number of children (Q02)	• Please use the age guide.<br>• The response should be one of the codes and not the child's age.
Q05	Existence of a vaccination card for the child	Total 0=<br>Total 1=	Total 0 + Total 1 = Total number of children (Q02)	• If the vaccination card is available or the guardian confirms that there is one, or was one (but not available, lost, or with another person, etc.), the answer corresponding to Q05 should be 1 "YES".<br>• If the child has never had a vaccination card or the guardian does not know if the child had one, the answer corresponding to Q05 should be 0 "NO".

				• **The vaccination card is proof of vaccines received by the child. If a vaccination card is available, the community volunteer should use the card to respond to questions about the child's vaccination status.**
**Q06**	**Does the child have a BCG scar on the left forearm?**	**Total 0=**<br>**Total 1=**	**Total 0 + Total 1 = Total number of children (Q02)**	• **If the child has a BCG scar on the left forearm, it is proof that the child was vaccinated at least one time and proof that a birth vaccine (BCG or OPV0) was received as of 0 months of age. The response corresponding to Q06 should be 1 "YES" and that for Q07 should be 1 "YES" also.**<br>• **If the child is absent and the guardian indicates that the child has a scar, the response corresponding to Q06 should be 1 "YES" and that for Q07 should be 1 "YES" also.**<br>• **If the child does not have a scar on the left forearm or if the child is absent, and the guardian indicates that the child does not have a scar or if the guardian does not know, the response corresponding to Q06 should be 0 "NO".**
**Q07**	**The child was vaccinated at least one time at a hospital or health center.**	**Total 0=**<br>**Total 1=**	**Total 0 + Total 1 = Total number of children (Q02)**<br><br>**Total 0 = children never-vaccinated**	• **Children never vaccinated at a hospital or health center are considered "never-vaccinated". The response corresponding to Q07 for such children should be 0 "NO". Vaccination through mass vaccination campaigns is not considered here.**<br><br>• **If the response to Q07 is 0 (child was never vaccinated), then:**<br>✓ **The response corresponding to Q09 should be 2: "Never vaccinated"**<br>✓ **The response corresponding to Q10 should be 2: "Never vaccinated"**<br>✓ **The response corresponding to Q11 should be 2: "Never vaccinated"**<br>✓ **The response corresponding to Q12 should be 2: "Never vaccinated"**<br>✓ **The response corresponding to Q13 should be 2: "Never vaccinated"**<br>✓ **The response corresponding to Q14 should be 2: "Never vaccinated"**<br>✓ **The response corresponding to Q15 should be j: Non applicable ("Never-vaccinated or completely vaccinated")**

				**If the child has a BCG scar on the left forearm, it is proof that the child was vaccinated at least one time and proof that a birth vaccine (BCG or OPV0) was received as of 0 months of age or if the vaccination card indicates that the child received at least one vaccine, the information on the card should be used by the community volunteer to respond to this question. The response to Q07 should be 1 "YES".
Q08	Reason the child was never vaccinated at a hospital or health center	Total a=<br>Total b=<br>Total c=<br>Total d=<br>Total e=<br>Total f=<br>Total g=<br>Total h=<br>Total i=<br>Total j=	Total a + Total b + Total c + Total d + Total e + Total f + Total g + Total h + Total i +Total j = Total number (Q02) of children<br><br>Total j "Child vaccinated" = Total 1 "YES" (Q07)	• Respond "Child vaccinated" (j): if the child was vaccinated at least one time at a hospital or health center.
Q09	Vaccines received as of birth (0 months) (BCG/OPV0)	Total 0=<br>Total 1=<br>Total 2=	Total 0 + Total 1 + Total 2=Total number of children (Q02)<br><br>Total 2 "Never vaccinated" = Total 0 "NO" (Q07)	✓ The response for children never-vaccinated is "2" (see above Q07).<br>✓ If the child received BCG or OPV0, the response should be 1 "YES". If not, the response is 0 "NO".<br><br>✓ The community volunteers will sometimes need to modify the information recorded on the questionnaire. For example:<br>✓ The child is aged 0 months (Q04)<br>✓ The child does not have a vaccination card (Q05=0)<br>✓ The child does not have a BCG scar on the left forearm (Q06=0)<br>✓ The guardian indicated that the child had been vaccinated (Q07=1), but the guardian said that the infant did not receive BCG or OPV0 (Q09 = 0)<br>✓ Consequently, the community volunteer must revise the information recorded as follows:<br>✓ The response to Q07 should be changed to 0: This child aged 0 months is considered never-vaccinated. The response to Q08 must be revised.

				✓ The response corresponding to Q09 should be 2: "Never vaccinated"<br>✓ The response corresponding to Q10 should be 2: "Never vaccinated"<br>✓ The response corresponding to Q11 should be 2: "Never vaccinated"<br>✓ The response corresponding to Q12 should be 2: "Never vaccinated"<br>✓ The response corresponding to Q13 should be 2: "Never vaccinated"<br>✓ The response corresponding to Q14 should be 2: "Never vaccinated"<br>✓ The response corresponding to Q15 should be j: Non applicable ("Never-vaccinated or completely vaccinated")
Q10	Vaccines received as of age 1½ months (one and a half months) (6 weeks)<br><br>(OPV1/PENTA1/PCV13(1)/ROTA1)	Total 0=<br>Total 1=<br>Total 2=<br>Total 3=	Total 0 + Total 1 + Total 2 + Total 3 =Total number of children (Q02)<br>Total 3 "Child too young" + Total 2 "never-vaccinated" >= Total 0 "NO" (Q07)	• "Never vaccinated" (2): for children never vaccinated (See above, Q07).<br>• "Child is too young" (3): for children too young to receive these vaccines.<br>• If the child received OPV1 or PENTA1 or PCV13(1) or ROTA1, the response is 1 "YES". If no, the response is 0 "NO".<br>• The community volunteer should verify the age of the child and that the child was old enough, or not, to receive these vaccines.
Q11	Vaccines received as of age 2½ months (two and a half months) (10 weeks)<br><br>(OPV2/PENTA2/PCV13(2)/ROTA2)	Total 0=<br>Total 1=<br>Total 2=<br>Total 3=	Total 0 + Total 1 + Total 2 + Total 3 =Total number of children (Q02)<br>Total 3 "Child too young" + Total 2 "never-vaccinated" > = Total 0 "NO" (Q07)	• "Never vaccinated" (2): for children never vaccinated (See above, Q07).<br>• "Child is too young" (3): for children too young to receive these vaccines.<br>• If the child received OPV2 or PENTA2 or PCV13(2) or ROTA2, the response is 1 "YES". If no, the response is 0 "NO".<br>• The community volunteer should verify the age of the child and that the child was old enough, or not, to receive these vaccines.
Q12	Vaccines received as of age 3½ months (three and a half months) (14 weeks)	Total 0=<br>Total 1=<br>Total 2=<br>Total 3=	Total 0 + Total 1 + Total 2 + Total 3 =Total number of children (Q02)	• "Never vaccinated" (2): for children never vaccinated (See above, Q07).<br>• "Child is too young" (3): for children too young to receive these vaccines.

	(OPV3/PENTA3/PCV13(3)/ROTA3/ IPV)		Total 3 "Child too young" + Total 2 "never-vaccinated" > = Total 0 "NO" (Q07)	• If the child received OPV3 or PENTA3 or PCV13(3) or ROTA3 or IPV, the response is 1 "YES". If no, the response is 0 "NO".<br>• The community volunteer should verify the age of the child and that the child was old enough, or not, to receive these vaccines.
Q13	Vaccines received as of 9 months<br><br>(MCV/YF)	Total 0=<br>Total 1=<br>Total 2=<br>Total 3=	Total 0 + Total 1 + Total 2 + Total 3 =Total number of children (Q02)<br>Total 3 "Child too young" + Total 2 "never-vaccinated" > = Total 0 "NO" (Q07)	• "Never vaccinated" (2): for children never vaccinated (See above, Q07).<br>• "Child is too young" (3): for children too young to receive these vaccines.<br>• If the child received MCV or YF, the response is 1 "YES". If no, the response is 0 "NO".<br>• The community volunteer should verify the age of the child and that the child was old enough, or not, to receive these vaccines.
Q14	Child completely vaccinated according to their age	Total 0=<br>Total 1=<br>Total 2=	Total 0 + Total 1 + Total 2=Total number of children (Q02)<br><br>Total 2 "never-vaccinated" = Total 0 "NO" (Q07)	• Rules for completing this column:<br>✓ First, if the answers to Q09, Q10, Q11, Q12, and Q13 are all « 2 », then the response to Q14 should be 2 "never-vaccinated" (see Q07 above).<br>✓ If there is at least one zero among the responses for Q09, Q10, Q11, Q12, and Q13, then the response to Q14 should be 0 "child is incompletely-vaccinated according to their age".<br>✓ If there are only 1s and 3s (no zeros or 2s) among responses for Q09, Q10, Q11, Q12, and Q1, then the response corresponding to Q14 should be 1 "child is completely-vaccinated according to their age".<br>• The community volunteer should verify that the vaccines noted as having been received correspond to the child's age.
Q15	Reason child is not vaccinated according to their age	Total a=<br>Total b=<br>Total c=<br>Total d=<br>Total e=<br>Total f=	Total j "never-vaccinated or completely-vaccinated" >= Total 0 "NO" (Q07)	• j "Not applicable (never-vaccinated or completely-vaccinated)" for children never-vaccinated (see above Q07).<br>• j « "Not applicable (never-vaccinated or completely-vaccinated)" for children completely vaccinated according to their age.

		**Total g=**<br>**Total h=**<br>**Total i=**<br>**Total j=**	**Total j "never-vaccinated or completely-vaccinated" = Total 1 "YES" (Q14) + Total 0 "NO" (Q07)**	
