# Supplementary material for: Reaching Never- and Incompletely-Vaccinated Children with Routine Immunization: A Proof-of-Concept Activity Using Geo-Referenced Microplans in Two Health Zones in Maniema Province, Democratic Republic of the Congo
Source: Vaccines (Basel). 2026 Feb 13;14(2):175. doi: 10.3390/vaccines14020175 (PMC12945245; doi:10.3390/vaccines14020175)
Supplement: Supplementary file 1 [file vaccines-14-00175-s001.zip › Tools_Alleman_Maniema_10Feb2026.zip!/E1_Tool_S5_Maniema_Kindu_Populations_By_Locality_French.pdf]

Kindu HZ, Maniema Province, RDC

Basoko (54 localités);*dénombré X 2.5 = **estimation total (T), jamais vaccinés (J), incomplètement vaccinés (I)						
Localité	0-23 mois 2022*			0-59 mois 2023**		
	T	J	I	T	J	I
Ba Jules	3	2	0	8	5	0
Baikuba	11	5	0	28	13	0
Bloc concasseur	121	51	4	303	128	10
Bloc sadala	39	11	0	98	28	0
Bokelele 1	127	78	6	318	195	15
Bokelele 2	129	69	12	323	173	30
Makuta	227	51	8	568	128	20
Sncc	181	27	11	453	68	28
De La Paix 1	61	10	2	153	25	5
De La Paix 2	39	18	0	98	45	0
DE L'HÔPITAL	103	18	3	258	45	8
Des jeunes	49	28	4	123	70	10
DIDINE KOPI	177	90	6	443	225	15
Ikese 1	31	4	0	78	10	0
Ikese 3	66	19	6	165	48	15
Industriel	129	34	4	323	85	10
Kambakamba Camp	3	3	0	8	8	0
Kampene	39	15	3	98	38	8
Kankulu	4	1	0	10	3	0
Kimbuyungu	172	87	3	430	218	8
Kowe	40	24	9	100	60	23
Loseke 1	95	44	5	238	110	13
Loseke 2	143	66	5	358	165	13
LOSEKE 3	79	39	4	198	98	10
Losomba	2	2	0	5	5	0
Lowa	71	42	1	178	105	3
Lubao 2	11	3	0	28	8	0
Lubenga	11	1	3	28	3	8
Lufungula 1	142	40	7	355	100	18
Lufungula 2	128	55	0	320	138	0
Lwalaba	42	35	6	105	88	15
Malonga	26	9	3	65	23	8
Mapindo	6	2	0	15	5	0
Masaka	131	61	0	328	153	0
Misekwa	17	4	0	43	10	0
Mitoni mbili	5	1	0	13	3	0
Moboko	12	5	0	30	13	0
Mongali	4	1	0	10	3	0
Mwanandeke Avenue	134	42	6	335	105	15
Ngongo 1	82	22	4	205	55	10
Ngongo 2	44	17	13	110	43	33
Okito 1	24	6	0	60	15	0
Okito 2	76	20	3	190	50	8
Pampela	171	85	18	428	213	45
Pensionné 1	48	10	2	120	25	5
Pensionné 2	139	68	1	348	170	3
Pensionné 3	122	39	3	305	98	8
Petro Congo	121	29	19	303	73	48
Tompi	2	0	0	5	0	0
Tuminga	122	45	17	305	113	43
Ubundu 1	111	18	8	278	45	20
Ubundu 2	191	72	13	478	180	33
Yenga 1	200	90	2	500	225	5
Yenga 2	129	28	6	323	70	15
	4392	1646	230	10980	4115	575

Brazza (40 localités); *dénombré X 2.5 = **estimation total (T), jamais vaccinés (J), incomplètement vaccinés (I)						
Localité	0-23 mois 2022*			0-59 mois 2023**		
	T	J	I	T	J	I
Ali	30	16	0	75	40	0
Bakari	32	21	0	80	53	0
Bethesaida	48	29	0	120	73	0
Djanga	21	8	0	53	20	0
Doky	34	25	0	85	63	0
Du Marche 1	22	13	0	55	33	0
EUGIDE	42	29	0	105	73	0
Inde	55	23	12	138	58	30
Kalidjiwe	63	40	0	158	100	0
Kasai	34	23	0	85	58	0
Kepoke 1	37	24	0	93	60	0
Kepoke 2	39	18	0	98	45	0
Kibinda	41	19	0	103	48	0
Kibombo 3	18	9	0	45	23	0
Likita	27	15	3	68	38	8
Lononga	86	68	1	215	170	3
Lukingama	25	16	0	63	40	0
Lutamba	72	49	0	180	123	0
Lwama Avenue	54	38	0	135	95	0
MAIMINGI	55	36	6	138	90	15
Manga 1	21	8	0	53	20	0
Manga 2	40	24	0	100	60	0
Manga 3	33	23	0	83	58	0
Maniema 3	39	27	0	98	68	0
Maniema 6	39	21	0	98	53	0
Manima Bis	20	12	0	50	30	0
Masangu	63	32	0	158	80	0
Mulu	39	17	4	98	43	10
Nafisa	41	16	0	103	40	0
Neno	132	95	1	330	238	3
Nouvelle	16	9	0	40	23	0
Omari 1	89	49	0	223	123	0
Salongo 1	30	11	0	75	28	0
Salongo 2	32	23	0	80	58	0
Shulungu 1	78	58	1	195	145	3
Tito	35	23	0	88	58	0
Uchudi 1	32	21	0	80	53	0
Unganda 1	53	29	0	133	73	0
Unganda 2	45	23	0	113	58	0
Utchudi 2	39	28	0	98	70	0
	1751	1068	28	4378	2670	70

Kasuku 1 (48 localités); *dénombré X 2.5 = **estimation total (T), jamais vaccinés (J), incomplètement vaccinés (I)						
Localité	0-23 mois 2022*			0-59 mois 2023**		
	T	J	I	T	J	I
3 Z	31	5	0	78	13	0
Bakadjika	40	5	0	100	13	0
Boma 2	24	7	0	60	18	0
Bv Joseph Kabila	126	13	0	315	33	0
Camp Office	73	31	1	183	78	3
Camp Prison	53	14	0	133	35	0
Chumbechumbe	123	40	13	308	100	33
Circo 1	120	46	0	300	115	0
DE L'HÔPITAL	43	12	1	108	30	3
Des Sport	57	5	15	143	13	38
Dibele 2	31	21	0	78	53	0
Du 24/Novembre Bis	168	0	22	420	0	55
Du 4 janvier	174	55	0	435	138	0
DU BAC	18	7	4	45	18	10
Du Commerce	10	2	0	25	5	0
Du Fleuve	31	19	2	78	48	5
Du Marche	46	20	0	115	50	0
Du rail stade	57	17	0	143	43	0
Du rail ville	69	22	0	173	55	0
Eveche 1	67	1	0	168	3	0
Eveche Bis	72	4	0	180	10	0
INGA DE LA JUSTICE	30	11	0	75	28	0
INSTALLATION SNCC	22	3	3	55	8	8
KAMAKAMA	144	16	0	360	40	0
KILIMA	61	7	0	153	18	0
Kizozo	30	3	1	75	8	3
LISAMBA	96	13	0	240	33	0
Lombe 1	93	2	0	233	5	0
Lubamba	29	11	0	73	28	0
LUMUMBA 3	24	10	3	60	25	8
LUNDULA	84	20	0	210	50	0
Lusambo 2	75	14	0	188	35	0
Lusangi	62	24	0	155	60	0
Maindombe	146	42	9	365	105	23
Malumalu	96	54	3	240	135	8
Mulamba	39	5	0	98	13	0
Musofu 1	69	16	14	173	40	35
Musofu 2	48	9	20	120	23	50
Mwendambali	83	8	0	208	20	0
Ngwangwata	112	24	7	280	60	18
Nkoy 1	97	12	4	243	30	10
NKOY 2	85	9	9	213	23	23
Numbiliki	72	16	7	180	40	18
Okenge 1	59	13	0	148	33	0
Potopoto 1	72	14	2	180	35	5
Regideso	245	89	0	613	223	0
Uchudi	56	15	3	140	38	8
YUMA	39	19	1	98	48	3
	3501	825	144	8753	2063	360

Kasuku 2 (35 localités); *dénombré X 2.5 = **estimation total (T), jamais vaccinés (J), incomplètement vaccinés (I)						
Localité	0-23 mois 2022*			0-59 mois 2023**		
	T	J	I	T	J	I
3 Z 1	36	6	0	90	15	0
3 Z 2	22	1	0	55	3	0
Baruani	15	8	0	38	20	0
Bloc Tchomba	32	12	2	80	30	5
Boma 1	47	11	12	118	28	30
Bunia	7	0	0	18	0	0
Mopaya	52	20	0	130	50	0
Carrière 1	29	9	4	73	23	10
Carrière 2	30	16	10	75	40	25
De La Coline	17	9	1	43	23	3
De l'évêché	5	1	0	13	3	0
De L'Univercite	19	11	1	48	28	3
Djamba 1	47	2	0	118	5	0
Djamba 2	22	4	6	55	10	15
Kabondo	48	18	11	120	45	28
Kananga 1	38	13	8	95	33	20
Kasongo 2	22	7	0	55	18	0
Kindu	21	7	0	53	18	0
Lusambo	13	0	2	33	0	5
Maniema 4	29	8	4	73	20	10
Maniema 2	18	2	3	45	5	8
Mununga 1	53	14	22	133	35	55
Mununga 2	49	15	13	123	38	33
Musijenge	44	18	0	110	45	0
Ocean	14	5	0	35	13	0
Olangati 1	132	40	1	330	100	3
Olangati 2	18	6	0	45	15	0
Otondo 1	25	10	0	63	25	0
Otondo 2	37	9	0	93	23	0
Pilipili	7	1	0	18	3	0
Ribariba	56	7	0	140	18	0
Salamabila 1	85	16	14	213	40	35
Salamabila 2	41	7	0	103	18	0
Tchambi	69	33	1	173	83	3
Tchambi 1	31	7	0	78	18	0
	1230	353	115	3075	883	288

Libenga (35 localités); *dénombré X 2.5 = **estimation total (T), jamais vaccinés (J), incomplètement vaccinés (I)						
Localité	0-23 mois 2022*			0-59 mois 2023**		
	T	J	I	T	J	I
BA RAMAZANI	8	0	0	20	0	0
BA ZAIRE	5	2	0	13	5	0
Basongola	5	3	1	13	8	3
FURAHA Avenue	37	1	2	93	3	5
Kamatanda	6	3	0	15	8	0
Kamitete City	3	0	0	8	0	0
Kanganga	2	0	0	5	0	0
Kanyanya	2	0	0	5	0	0
Kebile	3	1	0	8	3	0
Kepala	80	14	3	200	35	8
Kibaraka	139	35	4	348	88	10
KIKWAMA	5	0	0	13	0	0
KISHABONGO	56	4	0	140	10	0
Kitawala	3	1	0	8	3	0
Kitingi 1	30	5	0	75	13	0
Lombela	25	6	3	63	15	8
Losaka	7	3	0	18	8	0
Luyeye	4	1	0	10	3	0
LUYUBA	64	12	0	160	30	0
MABOLO OSOMBO CAMP	10	0	0	25	0	0
MOKOLOTCHE	30	1	2	75	3	5
MOTENDWA	83	18	1	208	45	3
Musa kayaya	7	2	0	18	5	0
Odimba	46	0	0	115	0	0
OSOMBO1	56	0	0	140	0	0
OSOMBO2	75	17	3	188	43	8
OSombo3	16	9	3	40	23	8
PAUL BABINGI	6	0	0	15	0	0
SIMON	8	1	3	20	3	8
Sudi	6	2	0	15	5	0
SUMBULANGA Camp	6	0	0	15	0	0
Tohoka 1	16	3	0	40	8	0
Tohoka 2	55	5	1	138	13	3
Tohoka 3	84	4	1	210	10	3
WASONGOLA	5	0	0	13	0	0
	993	153	27	2483	383	68

Lumbulumbu (22 localités); *dénombré X 2.5 = **estimation total (T), jamais vaccinés (J), incomplètement vaccinés (I)						
Localité	0-23 mois 2022*			0-59 mois 2023**		
	T	J	I	T	J	I
Atibu 1	116	26	15	290	65	38
Atibu 2	30	6	0	75	15	0
Butembo	216	62	2	540	155	5
Goma	152	65	12	380	163	30
Kabila I	139	56	21	348	140	53
Kinshasa 2	105	38	11	263	95	28
Kosa Kosa	131	73	0	328	183	0
Lukunda 1	105	36	7	263	90	18
Lukunda 2	78	49	3	195	123	8
Lumbulumbu	66	26	6	165	65	15
Lumumba	74	32	8	185	80	20
Makopo 1	84	25	1	210	63	3
Makopo 2	165	87	7	413	218	18
Makopo 3	65	15	5	163	38	13
Mayoko 1	97	35	0	243	88	0
Mayoko 2	125	56	0	313	140	0
Mayoko 3	129	66	1	323	165	3
Mbandaka District	160	95	15	400	238	38
Mpolo	54	31	0	135	78	0
Mukamba 1	86	31	2	215	78	5
Mukamba 2	57	21	0	143	53	0
Uvira	52	17	7	130	43	18
	2286	948	123	5715	2370	308

Lwama (39 localités); *dénombré X 2.5 = **estimation total (T), jamais vaccinés (J), incomplètement vaccinés (I)						
Localité	0-23 mois 2022*			0-59 mois 2023**		
	T	J	I	T	J	I
Aero	717	236	34	1793	590	85
Awazi	320	156	18	800	390	45
Ba Abeli	1	1	0	3	3	0
Ba Awazi	2	1	1	5	3	3
Ba TIPHON	1	1	0	3	3	0
Bilengalenga	3	2	0	8	5	0
Bisese	24	9	1	60	23	3
Camp Lwama	80	35	12	200	88	30
Kimbangu	24	2	0	60	5	0
Faba	36	8	0	90	20	0
Hewabora	576	131	18	1440	328	45
Kabele	5	5	0	13	13	0
Kabolongo City	373	187	21	933	468	53
Kakolende	1	1	0	3	3	0
Kasombe	5	5	0	13	13	0
Kenya	5	4	0	13	10	0
Kilabula	3	3	0	8	8	0
Lokema	3	2	0	8	5	0
Lomanga	1	1	0	3	3	0
Lombo ya Mozindo	4	3	0	10	8	0
Lwama City	299	149	20	748	373	50
Mabondo	15	13	0	38	33	0
Makoti meliya	24	21	0	60	53	0
Mangobo 1	527	199	51	1318	498	128
Mazembe	3	3	0	8	8	0
Mingazi	1	1	0	3	3	0
Mizizi	385	142	33	963	355	83
Modernité	683	310	53	1708	775	133
Mupasa	356	174	64	890	435	160
Ngombe	2	2	0	5	5	0
Omata	560	209	38	1400	523	95
Ongala 1,2	481	197	10	1203	493	25
Osombo 1	3	2	0	8	5	0
Penitanciel	604	247	42	1510	618	105
Yaka na Kongo	24	19	3	60	48	8
Yalombe	4	4	0	10	10	0
Yeriko	3	3	0	8	8	0
Yongima	10	9	0	25	23	0
Yongima City	2	0	0	5	0	0
	6170	2497	419	15425	6243	1048

Mikelenge (46 localités); *dénombré X 2.5 = **estimation total (T), jamais vaccinés (J), incomplètement vaccinés (I)						
Localité	0-23 mois 2022*			0-59 mois 2023**		
	T	J	I	T	J	I
117	24	9	5	60	23	13
1212/65	117	35	1	293	88	3
6Km/Rail	51	20	2	128	50	5
7Km/Rail	33	13	0	83	33	0
ANDRE KITCHINDJA	3	3	0	8	8	0
Baruti District	7	3	0	18	8	0
BARUTI/SWEDI	14	5	0	35	13	0
Bloc Hewa Bora	92	33	1	230	83	3
Bloc Kipalamoto	206	128	9	515	320	23
Bloc Tokedi 1	156	44	29	390	110	73
Bloc Tokedi 2	176	145	0	440	363	0
Bukavu	15	7	2	38	18	5
Buta	59	14	21	148	35	53
SUSA	3	2	0	8	5	0
DJANGA 1	37	16	9	93	40	23
FURAH	37	23	0	93	58	0
HEWA BORA	4	4	0	10	10	0
Irumu	82	34	6	205	85	15
Jean Frich	99	42	3	248	105	8
Kabambare	20	5	0	50	13	0
Kambakamba District	24	2	8	60	5	20
Kasongo 3	85	28	10	213	70	25
Kasuku 1	64	24	5	160	60	13
KIBOMBO 2	189	62	20	473	155	50
KINGOMBE	41	12	2	103	30	5
KINGOMBE/ OBETA	13	2	2	33	5	5
Kisangani	79	34	5	198	85	13
Kivu	110	39	13	275	98	33
Kongolo	68	22	4	170	55	10
Lokando	158	51	19	395	128	48
LOKOMBA KAITCHUYA	2	0	0	5	0	0
LOKOMBA MA ALIMA	6	6	0	15	15	0
Lubelenge	63	16	3	158	40	8
Lweki	15	5	0	38	13	0
Makopo	33	11	6	83	28	15
Maman Anie	154	38	9	385	95	23
Mbandaka	55	33	7	138	83	18
Mikelenge	113	33	32	283	83	80
Monuc	48	15	9	120	38	23
Niangara 2	91	49	12	228	123	30
Obeta	7	2	1	18	5	3
Onyembo	37	17	3	93	43	8
Swedi	15	4	0	38	10	0
VALENTIN	10	7	0	25	18	0
Venas	66	40	5	165	100	13
WANDJA	3	2	0	8	5	0
	2784	1139	263	6960	2848	658

RVA (39 localités); *dénombré X 2.5 = **estimation total (T), jamais vaccinés (J), incomplètement vaccinés (I)						
Localité	0-23 mois 2022*			0-59 mois 2023**		
	T	J	I	T	J	I
Aero 1	27	8	4	68	20	10
Bdom	67	26	0	168	65	0
Boulevard Joseph Kabila	14	3	0	35	8	0
Bouve	76	17	0	190	43	0
BUSHIRI	57	22	3	143	55	8
Monusco	70	10	0	175	25	0
Chute	54	20	8	135	50	20
Cinquantenaire	90	38	7	225	95	18
Cite De Jeune	116	36	16	290	90	40
Clinique Universitaire	28	5	10	70	13	25
De La Paix	37	20	3	93	50	8
DIDI	153	40	3	383	100	8
Elila	77	28	16	193	70	40
EPA basoko	89	42	16	223	105	40
Ferme	116	39	32	290	98	80
Kamundala	107	51	22	268	128	55
Kiboko	91	19	2	228	48	5
Kilimadjaro	56	15	0	140	38	0
Kisima Pori	65	21	13	163	53	33
Makila	73	10	16	183	25	40
Miba	46	12	5	115	30	13
Mufula	146	32	8	365	80	20
Mukaya	85	27	4	213	68	10
Mungazi	69	15	1	173	38	3
Mwanandeke 1	157	34	5	393	85	13
Mwanandeke 2	139	45	9	348	113	23
Ngwangwata District	29	10	5	73	25	13
Nyangwe	63	17	2	158	43	5
Nyombo	90	11	0	225	28	0
Okapi	67	22	4	168	55	10
Oms	48	8	0	120	20	0
Pepiniere	70	12	7	175	30	18
Ponio	60	31	0	150	78	0
SALAMA 1	115	49	6	288	123	15
SALAMA 2	60	22	12	150	55	30
Steven	75	18	4	188	45	10
Tchololo District	101	39	13	253	98	33
Toka	74	22	20	185	55	50
Tuungane	63	12	2	158	30	5
	3020	908	278	7550	2270	695

Tokolote (57 localités); *dénombré X 2.5 = **estimation total (T), jamais vaccinés (J), incomplètement vaccinés (I)						
Localité	0-23 mois 2022*			0-59 mois 2023**		
	T	J	I	T	J	I
100 MAISONS	52	31	4	130	78	10
Amani	44	23	0	110	58	0
B doom	17	4	0	43	10	0
Bakongola	139	60	10	348	150	25
BAMBO	20	10	2	50	25	5
Bandal	29	10	1	73	25	3
Baraka	63	36	1	158	90	3
Belle Aire	66	23	0	165	58	0
Bloc Panda	76	49	16	190	123	40
Blvd idrisa	107	77	3	268	193	8
Boma 3	14	2	5	35	5	13
De L'Antene	71	42	1	178	105	3
Dela justice	21	10	2	53	25	5
Dela paix	20	5	0	50	13	0
Dibele 1	133	63	2	333	158	5
Didjango	106	78	1	265	195	3
Enano	61	41	6	153	103	15
Kabolili	23	11	6	58	28	15
KAMWENA	33	11	2	83	28	5
Kananga	107	38	12	268	95	30
Kasese	29	12	0	73	30	0
Kasumba	57	35	2	143	88	5
Kizozo	44	12	0	110	30	0
Lokenye	109	28	5	273	70	13
Lomame	208	93	14	520	233	35
Lononga	66	34	0	165	85	0
Lubefu	90	60	0	225	150	0
Lusangi	98	39	0	245	98	0
Lutu	19	9	0	48	23	0
LWALABA 1	97	51	7	243	128	18
Lwama 1	18	14	0	45	35	0
Maendeleo	34	15	4	85	38	10
Malumalu	79	37	7	198	93	18
Maniema	20	8	0	50	20	0
Marcel	13	6	5	33	15	13
Matadi	194	95	5	485	238	13
Mikelenge	18	9	0	45	23	0
Mont Ngaliema	46	23	0	115	58	0
Mukindji	20	12	4	50	30	10
Mussa	41	22	0	103	55	0
Mwinyi	1	0	0	3	0	0
Ngoy	7	4	0	18	10	0
Numbiliki	52	27	0	130	68	0
Okenge	32	10	0	80	25	0
Okoko	7	2	3	18	5	8
Omari	90	65	0	225	163	0
Ombeka	41	28	2	103	70	5
OMENYA	48	26	0	120	65	0
Onyonga	84	20	0	210	50	0
RAPHAËL	30	22	0	75	55	0
Sankuru	199	101	13	498	253	33
Sayo	45	22	0	113	55	0
Shulungu	50	24	3	125	60	8
Taratibu	14	7	0	35	18	0
TCHOLOLO	48	24	0	120	60	0
Utchudi	24	14	0	60	35	0
Victoire	37	23	5	93	58	13
	3311	1657	153	8278	4143	383

Trois Z (15 localités); *dénombré X 2.5 = **estimation total (T), jamais vaccinés (J), incomplètement vaccinés (I)						
Localité	0-23 mois 2022*			0-59 mois 2023**		
	T	J	I	T	J	I
Aero Bis	32	10	4	80	25	10
Air Congo	22	1	1	55	3	3
Boulevard	14	3	1	35	8	3
Cardinal	42	6	5	105	15	13
Elongo	21	3	3	53	8	8
Eveche	2	0	1	5	0	3
Eveche Bis 1	4	1	0	10	3	0
Kithima	29	6	3	73	15	8
Lwambo	38	8	1	95	20	3
Lwangi	32	13	3	80	33	8
Lwembe	31	3	9	78	8	23
Openda 1	30	13	4	75	33	10
Openda 2	29	5	8	73	13	20
Tchomba	31	5	19	78	13	48
Trois Z	42	17	3	105	43	8
	399	94	65	998	235	163
