# Supplementary material for: Reaching Never- and Incompletely-Vaccinated Children with Routine Immunization: A Proof-of-Concept Activity Using Geo-Referenced Microplans in Two Health Zones in Maniema Province, Democratic Republic of the Congo
Source: Vaccines (Basel). 2026 Feb 13;14(2):175. doi: 10.3390/vaccines14020175 (PMC12945245; doi:10.3390/vaccines14020175)
Supplement: Supplementary file 1 [file vaccines-14-00175-s001.zip › Tools_Alleman_Maniema_10Feb2026.zip!/F1_Tool_S6A_Maniema_PIRI_Vaccines_By_Age_Graphic_French.pdf]

Activités de vaccination, 2023/2024, Projet de renforcement de la vaccination de routine chez des enfants non-vaccinés ou insuffisamment vaccinés à l'aide de micro plans validés dans les zones de santé à haut risque de transmission de la polio et de la rougeole, Kindu/Kibombo, Maniema, RDC

Age de l'enfant	Vaccins disponibles pour les activités de vaccination	Quand administrer	Ne pas administrer
A la naissance	BCG; VPOb 0	VPOb jusqu'à 14 jours; BCG jusqu'à 23 mois	
6 semaines/ $1\frac{1}{2}$ mois	VPOb 1; Penta 1; Rota 1; PCV 1	Dés que possible après l'âge de 6 semaines	Avant l'âge de 6 semaines; ROTA 1 après l'âge de 23 mois
10 semaines $2\frac{1}{2}$ mois	VPOb 2; Penta 2; Rota 2; PCV 2	Dés que possible après l'âge de 10 semaines et au minimum 4 semaines après la première dose.	Moins de 4 semaine après les doses précédents; ROTA 2 après l'âge de 23 mois
14 semaines $3\frac{1}{2}$ mois	VPOb 3; Penta 3; Rota 3; PCV 3; VPI 1	Dés que possible après l'âge de 14 semaines et au minimum 4 semaines après la deuxième dose.	Moins de 4 semaine après les doses précédents; ROTA 3 après l'âge de 23 mois
9 mois	VPI 2; VAR 1; VAA	Dés que possible après l'âge de 9 mois; aucune limite supérieure d'âge	Avant l'âge de 9 mois
12 mois	PCV 1; PCV 2	Dés que possible après l'âge de 12 mois et au minimum 4 semaines après PCV 1	
15 mois	VAR 2	Dés que possible après l'âge de 15 mois et au minimum 4 semaines après VAR 1	VAR2: Moins de 4 semaines après le VAR 1
24-59 mois	PCV 1	Dés que possible après l'âge de 24 mois	

59 mois

Il n'est pas nécessaire de reprendre depuis le début l'administration des doses des vaccines même s'il s'est écolulé beaucoup de temps entre les doses. Il n'y a pas de limite supérieure d'âge pour les doses de vaccine — à l'exception des vaccins BCG et ROTA qui ne seront pas administrés après l'âge de 23 mois. Graphique modifié à partir de celui figurant à la page 29 du document de l'Organisation mondiale de la santé intitulé « Ne laisser personne de côté : Guide pour la planification et la mise en œuvre de la vaccination de rattrapage ». 2021. https://www.who.int/publications/i/item/9789240016514
