# Supplementary material for: Reaching Never- and Incompletely-Vaccinated Children with Routine Immunization: A Proof-of-Concept Activity Using Geo-Referenced Microplans in Two Health Zones in Maniema Province, Democratic Republic of the Congo
Source: Vaccines (Basel). 2026 Feb 13;14(2):175. doi: 10.3390/vaccines14020175 (PMC12945245; doi:10.3390/vaccines14020175)
Supplement: Supplementary file 1 [file vaccines-14-00175-s001.zip › Tools_Alleman_Maniema_10Feb2026.zip!/F2_Tool_S6B_Maniema_PIRI_Vaccines_By_Age_Graphic_English.pdf]

Vaccination Activities, 2023/2024, Project to strengthen routine immunization among unvaccinated or incompletely children using validated micro-plans in the Health Zones at the highest risk of poliovirus and measles transmission, Kindu/Kibombo, Maniema, RDC

Vaccines Available during Vaccination Activities

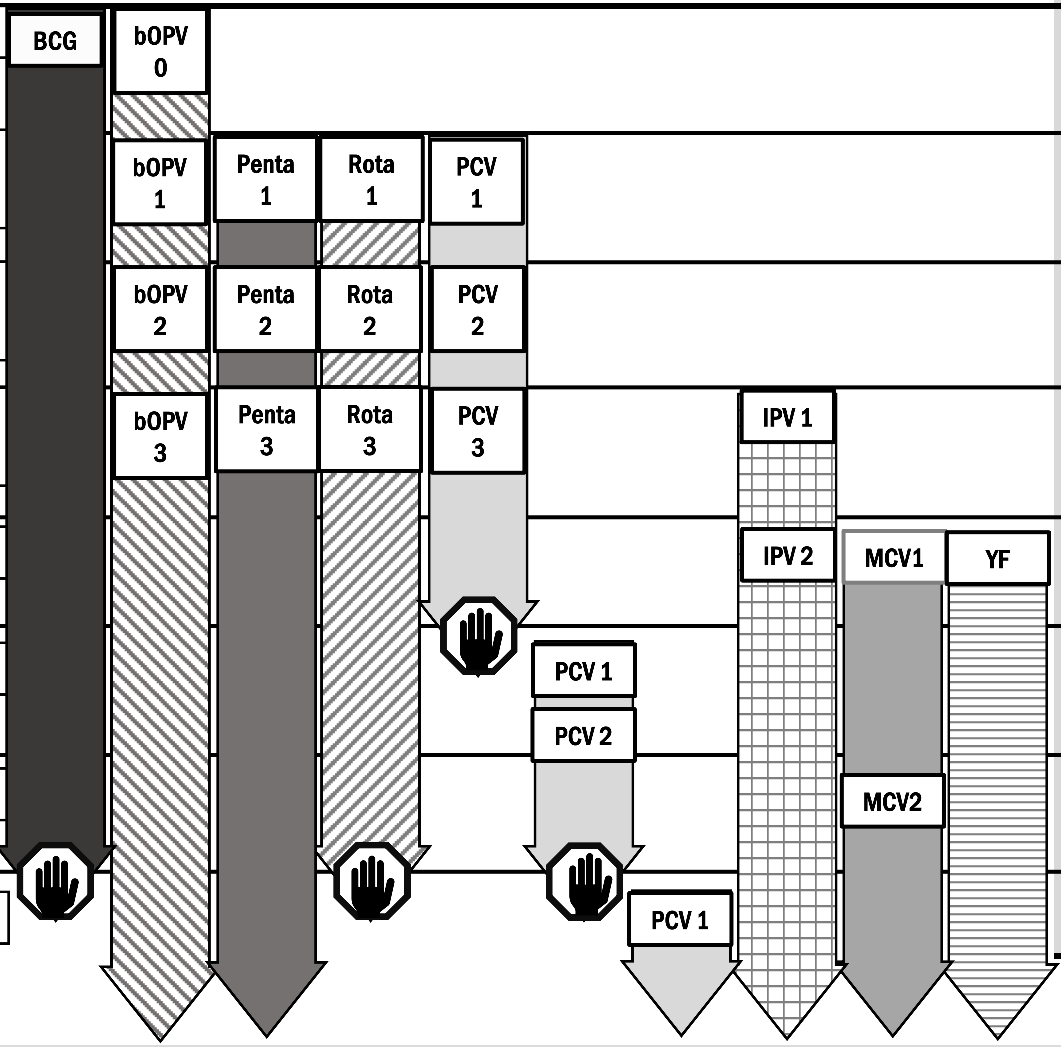

Child age	When to give	When not to give
Birth	bOPV until 14 days; BCG until 23 months	
6 weeks/ $1\frac{1}{2}$ months	As soon as possible after 6 weeks	Before the age of 6 weeks; ROTA 1 after the age of 23 months
10 weeks/ $2\frac{1}{2}$ months	As soon as possible after the age of 10 weeks and a minimum of 4 weeks after the previous dose.	Less than 4 weeks since the preceding dose; ROTA 2 after the age of 23 months
14 weeks/ $3\frac{1}{2}$ months	As soon as possible after the age of 14 weeks and at least 4 weeks after the second dose.	Less than 4 weeks since the preceding dose; ROTA 3 after the age of 23 months
9 months	As soon as possible after 9 months; no upper age limit	Before the age of 9 months
12 months	As soon as possible after 12 months of age and at least 4 weeks after PCV 1	
15 months	As soon as possible after 15 months of age and a minimum of 4 weeks after MCV 1	MCV2: Less than 4 weeks after MCV1
24-59 months	As soon as possible after the age of 24 months	

59 months

Even if a long time has passed between doses, there is no need to restart the series from the beginning. There is no upper age limit for most vaccines (except rotavirus and BCG, which cannot be administered after 23 months of age). This figure has been modified from a corresponding Figure on page 29 in the World Health Organization document, « Leave no one behind: guidance for planning and implementing catch up vaccination » 2021. https://www.who.int/publications/i/item/9789240016514
