# Supplementary material for: Reaching Never- and Incompletely-Vaccinated Children with Routine Immunization: A Proof-of-Concept Activity Using Geo-Referenced Microplans in Two Health Zones in Maniema Province, Democratic Republic of the Congo
Source: Vaccines (Basel). 2026 Feb 13;14(2):175. doi: 10.3390/vaccines14020175 (PMC12945245; doi:10.3390/vaccines14020175)
Supplement: Supplementary file 1 [file vaccines-14-00175-s001.zip › Tools_Alleman_Maniema_10Feb2026.zip!/G1_Tool_S7A_Maniema_AgeGuide_First_PIRI_French.pdf]

Guide d'age 0-59 mois, Activites de ratrappage en RDC
Passage Septembre 2023 (les groupes d'âge sont approximatifs ; utilisez la date de naissance si connu)

Mois/Année	Janvier	Février	Mars	Avril	Mai	Juin	Juillet	Aout	Septembre	Octobre	Novembre	Décembre
2023	8	7	6	5	4	3	2	1	0			
	3 ½ (14 semaines) - 8 mois					2 ½ mois 10 semaines	1 ½ mois 6 semaines	0	0			

Mois/Année	Janvier	Février	Mars	Avril	Mai	Juin	Juillet	Aout	Septembre	Octobre	Novembre	Décembre
2022	20	19	18	17	16	15	14	13	12	11	10	9
	15 - 23 mois						12-14 mois			9-11 mois		

Mois/Année	Janvier	Février	Mars	Avril	Mai	Juin	Juillet	Aout	Septembre	Octobre	Novembre	Décembre
2021	32	31	30	29	28	27	26	25	24	23	22	21
	24-59 mois									15-23 mois		

Mois/Année	Janvier	Février	Mars	Avril	Mai	Juin	Juillet	Aout	Septembre	Octobre	Novembre	Décembre
2020	44	43	42	41	40	39	38	37	36	35	34	33
	24-59 mois											

Mois/Année	Janvier	Février	Mars	Avril	Mai	Juin	Juillet	Aout	Septembre	Octobre	Novembre	Décembre
2019	56	55	54	53	52	51	50	49	48	47	46	45
	24-59 mois											

Mois/Année	Janvier	Février	Mars	Avril	Mai	Juin	Juillet	Aout	Septembre	Octobre	Novembre	Décembre
2018										59	58	57
										24-59 mois		
