# Supplementary material for: Reaching Never- and Incompletely-Vaccinated Children with Routine Immunization: A Proof-of-Concept Activity Using Geo-Referenced Microplans in Two Health Zones in Maniema Province, Democratic Republic of the Congo
Source: Vaccines (Basel). 2026 Feb 13;14(2):175. doi: 10.3390/vaccines14020175 (PMC12945245; doi:10.3390/vaccines14020175)
Supplement: Supplementary file 1 [file vaccines-14-00175-s001.zip › Tools_Alleman_Maniema_10Feb2026.zip!/G2_Tool_S7B_Maniema_AgeGuide_First_PIRI_English.pdf]

Age Guide 0-59 months, Vaccination activities in DRC
PIRI round September 2023 (age groupings are approximate; use the child's birth date if known)

Month/Year	January	February	March	April	May	June	July	August	September	October	November	December
2023	8	7	6	5	4	3	2	1	0			
	$3\frac{1}{2}$ (14 weeks) - 8 months					$2\frac{1}{2}$ months 10 weeks	$1\frac{1}{2}$ months 6 weeks	0	0			

Month/Year	January	February	March	April	May	June	July	August	September	October	November	December
2022	20	19	18	17	16	15	14	13	12	11	10	9
	15 - 23 months						12 - 14 months			9 - 11 months		

Month/Year	January	February	March	April	May	June	July	August	September	October	November	December
2021	32	31	30	29	28	27	26	25	24	23	22	21
	24-59 months									15-23 months		

Month/Year	January	February	March	April	May	June	July	August	September	October	November	December
2020	44	43	42	41	40	39	38	37	36	35	34	33
	24-59 months											

Month/Year	January	February	March	April	May	June	July	August	September	October	November	December
2019	56	55	54	53	52	51	50	49	48	47	46	45
	24-59 months											

Month/Year	January	February	March	April	May	June	July	August	September	October	November	December
2018										59	58	57
										24-59 months		
