# Supplementary material for: Reaching Never- and Incompletely-Vaccinated Children with Routine Immunization: A Proof-of-Concept Activity Using Geo-Referenced Microplans in Two Health Zones in Maniema Province, Democratic Republic of the Congo
Source: Vaccines (Basel). 2026 Feb 13;14(2):175. doi: 10.3390/vaccines14020175 (PMC12945245; doi:10.3390/vaccines14020175)
Supplement: Supplementary file 1 [file vaccines-14-00175-s001.zip › Tools_Alleman_Maniema_10Feb2026.zip!/H1_Tool_S8A_Maniema_PIRI_Coupon_Vaccines_Needed_French.pdf]

Activités de vaccination 2023/2024. Projet de renforcement de la vaccination de routine chez des enfants non-vaccinés ou insuffisamment vaccinés à l'aide de micro-plan validés dans les zones de sante à haut risque de transmission de la polio et de la rougeole, Kindu/Kibombo, Maniema, RDC

Jeton de rattrapage vaccinal, 2023/2024

Ticket de référence pour la vaccination systématique

Emmener la carte avec vous au poste de vaccination

Zone de santé: ____________ Aire de santé: ____________

Localité: ____________

Nom de l'enfant: ____________

L'âge de l'enfant: 0-11 mois ☐ 12-23 mois ☐ 24-59 mois ☐

Enfant jamais vacciné à l'hôpital ou centre de santé? Oui ☐ No ☐

Enfant est incomplètement vacciné selon son âge? Oui ☐ No ☐

Localisation du poste de vaccination: ____________

nom de RECO 1er passage ____________ Date ____________

nom de RECO 2e passage ____________ Date ____________

nom de RECO 3e passage ____________ Date ____________
