# Supplementary material for: Reaching Never- and Incompletely-Vaccinated Children with Routine Immunization: A Proof-of-Concept Activity Using Geo-Referenced Microplans in Two Health Zones in Maniema Province, Democratic Republic of the Congo
Source: Vaccines (Basel). 2026 Feb 13;14(2):175. doi: 10.3390/vaccines14020175 (PMC12945245; doi:10.3390/vaccines14020175)
Supplement: Supplementary file 1 [file vaccines-14-00175-s001.zip › Tools_Alleman_Maniema_10Feb2026.zip!/H2_Tool_S8B_Maniema_PIRI_Coupon_Vaccines_Needed_English.pdf]

Vaccination Activities 2023/2024. Project to reinforce routine vaccination among children never vaccinated or incompletely vaccinated using validated microplans in health zones at high risk for transmission of measles and polio, Kindu and Kibombo, Maniema Province, DRC

Coupon for Catch-Up Vaccination, 2023/2024

Ticket de référence pour la vaccination systématique

Bring this card with you to the vaccination site

Health Zone: ____________________ Health Area: ____________________

Locality: ____________________

Name of the child: ____________________

Age of the child: 0-11 months ☐ 12-23 months ☐ 24-59 months ☐

Was child ever vaccinatedat a hospital or health center? Yes ☐ No ☐

Is the child incompletely vaccinated accirding to their age? Yes ☐ No ☐

Location of vaccination site: ____________________

Name of volunteer 1st PIRI ____________________ Date ____________

Name of volunteer 2nd PIRI ____________________ Date ____________

Name of volunteer 3rd PIRI ____________________ Date ____________
