# Supplementary material for: Reaching Never- and Incompletely-Vaccinated Children with Routine Immunization: A Proof-of-Concept Activity Using Geo-Referenced Microplans in Two Health Zones in Maniema Province, Democratic Republic of the Congo
Source: Vaccines (Basel). 2026 Feb 13;14(2):175. doi: 10.3390/vaccines14020175 (PMC12945245; doi:10.3390/vaccines14020175)
Supplement: Supplementary file 1 [file vaccines-14-00175-s001.zip › Tools_Alleman_Maniema_10Feb2026.zip!/I1_Tool_S9A_Maniema_PIRI_TrainingMaterials_French.pdf]

FORMATION DES ACTEURS IMPLIQUES DAND LE SECOND TOUR DE L'INTENSIFICATION PERIODIQUE DE LA VACCINATION SYSTEMATIQUE (IPVS)

Manuel de formation des coordonnateurs des aires de santé, des superviseurs d'axes et des Presi-Codesa.

Note au sujet de la source des outils référés dans le manuel :

- Outil élaboré ou adapté par l'équipe CDC/GRID3
- Outil élaboré par le centre de santé/l'équipe cadre de la zone de santé

I. Les formations à effectuer :

Trois types de formation qui regroupent des acteurs aux responsabilités similaires :

Formation	Cible (les personnes qui vont être formées)	Durée	Payée
Formation des coordonnateurs des aires de santé, des superviseurs d'axes et des Presi-Codesa	IT, Presi-Codesa et agents bureau central de la zone	1 jour	Oui
Formation des vaccinateurs et des encodeurs	ITA, autres infirmiers et encodeurs	1 jour	Non
Formation des relais communautaires	Relais communautaires (crieurs, identification)	1 jour	Non

Trois manuels de formation seront disponibles et serviront de rappel pour les activités à mener en lien avec l'IPVS :

- Manuel de formation des coordonnateurs des aires de santé, des superviseurs d'axes et des Presi-Codesa.
- Manuel de formation des vaccinateurs et des encodeurs (voir Page 9).
- Manuel de formation des relais communautaires (voir Page 10).

II. Le contexte de l'IPVS :

1. **Cible en termes d'âge** :

   La cible pour l'IPVS comprend les enfants de 0 à 59 mois qui n'ont jamais été vaccinés ou qui ne sont pas complètement vaccinés selon leur âge.

2. **Nombre d'enfants attendus par localité :**

Pour déterminer le nombre d'enfants attendus dans chaque localité, il est suggéré d'utiliser les estimations sur les listes des localités [fiches vertes (Kindu), oranges (Kibombo)].

3. **Les antigènes et le calendrier de vaccination :**

Le but est d'administrer huit antigènes (BCG, VPOB, Penta, PCV13, ROTA, VPI, VAR, VAA) et d'utiliser le graphique « Antigènes selon l'âge », le guide d'âge et le calendrier vaccinal pour guider l'administration des vaccins.

III. Actions à mener avant la campagne :

1. Sensibilisation (IT + Presi-Codesa) :

1.1. Sélection des relais communautaires :

Il est conseillé d'utiliser les listes de relais communautaires reconduits pour la phase 2 sur la base de la performance durant la phase 1 et de designer les crieurs pour chaque équipe de vaccination (2 crieurs par équipe à Kindu et 2 crieurs par village/quartier à Kibombo).

L'IT doit mettre le plan de communication à la disposition des Presi-Codesa.

1.2. Gestion des jetons jaunes :

Donner un nombre suffisant de jetons jaunes aux relais (prévoir plus que la cible d'enfants pour éviter les ruptures) et demander au Presi-Codesa de faire le suivi de la distribution des jetons.

1.3. Identification et ratissage des enfants :

- Les relais communautaires vont utiliser la fiche de suivi des distributions des jetons jaunes aux enfants zéros dose et insuffisamment vaccinés par les relais pour fournir des informations fiables sur les ménages visités et sur le nombre d'enfants cibles identifiés.
- Identifier tous les enfants qui ont besoin d'être vaccinés, sur la base des cartes CPS, des témoignages des mamans, des fiches de dénombrement de 2022, des listes des enfants à récupérer disponibles dans les centres de santé, etc.
- Informer les ménages au sujet des séances de vaccinations : rappeler les dates et les sites de vaccinations.

1.4. Remplissage des jetons jaunes :

S'assurer que les jetons jaunes sont correctement remplis (nom enfant, localité, sexe, âge, statut vaccinal, site de vaccination, la date de distribution du jeton jaune et le nom du relais).

1.5. Supervision par le Presi-Codesa :

Les Presi-Codesa doivent être impliqués dans la sélection des relais communautaires, dans la gestion et supervision des relais communautaires sur le terrain ainsi que dans la distribution des jetons jaunes. Ils doivent s'assurer que les crieurs passent dans les rues autour des sites pour informer au sujet de la vaccination et de la localisation des sites de vaccination. Ils doivent s'assurer de sélectionner des acteurs familiers avec leur avenue/quartier, qui sont disponibles pour toute la durée de l'activité et qui bénéficient de la confiance de la communauté.

1.6. Paiement :

Le paiement sera effectué par Mobile money, ce qui nécessite la mise à jour et la transmission des listes de personnes à payer.

Les Presi-Codesa recevront 10$ par personne pour toute la durée de l'activité, les relais communautaires recevront 8$ par personne pour toute la durée de l'activité, les crieurs recevront 5$ par personne pour toute la durée de l'activité.

2. Plan de déploiement des équipes (IT)

2.1. Sélection des vaccinateurs et des encodeurs :

2.1.1. Vaccinateurs :

- La sélection devra privilégier ceux qui sont familiers avec la vaccination de routine et l'évaluation du statut vaccinal après interrogation des mamans ou consultation des jetons jaunes, qui sont capables d'administrer les vaccins, et qui maitrisent le remplissage des jetons bleus, des cartes CPS, des fiches de provenance, des fiches de pointage et des fiches synthèses des fiches de pointage et de provenance. Il faudra également privilégier les acteurs qui sont crédibles et disponibles pour toute la durée de l'activité.
- Le premier vaccinateur doit communiquer les vaccins à administrer au deuxième vaccinateur à l'aide d'un bout de papier.
  Note : la fiche de provenance est remplie par le vaccinateur 1. La fiche de pointage est remplie par le vaccinateur 2.

2.1.2. Encodeurs :

- La sélection devra privilégier ceux qui sont capables de bien comprendre les fiches de provenance et les fiches de pointage, d'entrer les données de ces fiches dans le téléphone et qui sont capables de remplir les fiches synthèses des fiches de pointage et de provenance ainsi que le cahier de vaccination.
  Note : La fiche de provenance et la fiche de pointage sont remplies par les vaccinateurs, mais l'encodeur doit également être familier avec le remplissage de ces deux fiches pour bien rentrer l'information dans le téléphone.
- Les encodeurs doivent garder les jetons jaunes.

2.2. Mouvement des équipes :

2.2.1. Réviser la cartographie des mouvements des équipes :

- Le mouvement des équipes sera basé sur la cartographie des mouvements des équipes élaborée lors de la micro-planification.

- Il faudra s'assurer que les relais communautaires sont familiers avec la cartographie des mouvements des équipes pour donner la bonne information à la population au sujet des sites de vaccinations.

2.2.2. Respecter les mouvements des sites :

S'assurer que les équipes, avec tous leurs membres au complet, soient présentes dans tous les sites planifiés pour chacun des jours de vaccination prévus.

2.2.3. Priorisation sites éloignés :

Demander aux équipes assignées aux sites éloignés ou à accès difficile de commencer les séances de vaccination par les sites les plus éloignés pour terminer par les sites les plus proches pour s'assurer de rejoindre le maximum des enfants qui vivent dans ces zones.

2.2.4. Outils :

- Cartes de regroupement des villages (rayon de 500 m en zone urbaine et 5km en zone rurale) et des centres de santé avec une chaine de froid opérationnelle.
- Listes des membres des équipes de vaccination (vaccinateurs et encodeurs).
- Equipes de vaccination selon le microplan

3. Formation des vaccinateurs, encodeurs et relais communautaires (IT, Presi-Codesa, agents du bureau central de la zone)

3.1. Calendrier de formation :

Former les acteurs avec des séances pratiques dans les ménages ou dans les sites de simulation.

Pour les relais communautaires, former suffisamment à l'avance pour faire une bonne sensibilisation et bien identifier les enfants à vacciner dans la communauté (prévoir au moins 3 jours pour l'identification des enfants et 2 jours de ratissage).

A la fin de la formation :

- Remettre aux vaccinateurs et aux encodeurs le Manuel de formation des vaccinateurs et des encodeurs.
- Remettre aux relais communautaires le Manuel de formation des relais communautaires.

3.2. Calendrier d'accompagnement :

Respecter le calendrier d'accompagnement élaboré avec le bureau central zone de santé avec le soutien de GRID3.

4. Sites de stockage (IT) :

- Identifier les sites de stockage de relais et établir un plan de ravitaillement avec l'appui du bureau central de la zone.
- Prévoir le ravitaillement avec une avance sur le début des activités de vaccination.

- Communiquer aux équipes de vaccination la localisation des sites de stockage.

5. **Préfinancement des équipes pour les zones à accès difficile (IT) :**
    - Les ITs doivent se porter garant de l'utilisation des fonds préfinancés pour atteindre les sites éloignés et / ou à accès difficile et vacciner les enfants.
    - Les membres des équipes assignés aux sites éloignés et / ou à accès difficile doivent signer un acte d'engagement pour le transport.

6. **Modalités de paiement :**

    - Le paiement se fera par mobile money et nécessite la mise à jour des listes des membres des équipes de vaccination (vaccinateurs et encodeurs).
    - Les vaccinateurs et les encodeurs recevront 5$ par personne, par jour, pendant 5 jours sur la base de l'évaluation de la performance.

IV. Actions à mener pendant la campagne :

1. **Techniques vaccinales (IT) :**

   Respecter les normes du Plan Elargi de Vaccination (PEV) pour la conservation, la préparation des vaccins, l'administration des vaccins, la gestion des déchets et la gestion des Manifestations Adverses Post Immunisation (MAPI).

2. **Organisation du site (IT) :**
    - Chaque équipe est composée de trois membres (deux vaccinateurs et un encodeur).
    - L'organisation du site suit le schéma proposé dans l'outil « Circuit de Vaccination »
    - L'équipe doit se procurer des tables et des chaises, en demandant l'aide de la communauté, au besoin, pour bien organiser le site de vaccination.
    - Les responsabilités des membres de l'équipe de vaccination :
        1. Les responsabilités du Vaccinateur 1 : vérifier le statut vaccinal des enfants, communiquer les vaccins à administrer au deuxième vaccinateur sur un bout de papier, remplir les généralités dans les cartes CPS et jeton bleu, remplir la fiche de provenance sur papier au fur et à mesure de l'arrivée des enfants, expliquer les effets secondaires et la conduite à tenir en cas de MAPI, inviter les enfants pour la prochaine IPVS, faire la gestion des déchets, s'assurer que l'encodeur a saisi les données dans le téléphone, remplir la fiche synthèses des fiches de pointage et de provenance avec le reste de l'équipe a la fin de la journée.

        NOTE IMPORTANTE :

        - **La localité des enfants dans la fiche de pointage est la localité du lieu de résidence/provenance de l'enfant, pas la localité du site de vaccination. Se servir de la liste de localité fournie à l'équipe pour inscrire la bonne localité.**
        - **Les informations pour la gestion des MAPI se trouvent au revers de la fiche de Pointage.**

2. Les responsabilités du Vaccinateur 2 : valider le statut vaccinal des enfants, préparer et administrer les vaccins, remplir les informations vaccinales dans les cartes CPS et le jeton bleu et dans la fiche de pointage.
3. Les responsabilités de l'Encodeur :
   - Pendant la séance : remplir la fiche de provenance sur téléphone pendant les temps morts (temps ou le circuit n'a pas d'enfants à vacciner), inviter les enfants pour la prochaine IPVS, remplir les informations vaccinales dans le cahier de vaccination.
   - A la fin de la séance : terminer la saisie de la fiche de provenance dans le téléphone, saisir la fiche de pointage dans le téléphone
   - S'assurer que les fiches de provenance et de pointage sont rentrées dans le téléphone au site même de vaccination.
4. Les responsabilités de l'équipe :
   - Remplir la fiches synthèses des fiches de pointage et de provenance à la fin de la journée.

3. **Supervision :**
   - Les Presi-Codesa vont effectuer la supervision des relais communautaires et des crieurs à l'aide de l'outil fiche de suivi des distributions des jetons jaunes aux enfants zéros dose et insuffisamment vaccinés par les relais
   - Les IT et les agents du bureau central de la zone vont effectuer la supervision des vaccinateurs et des encodeurs
   - Tous les superviseurs (à l'exception des Presi-Codesa) vont utiliser une Formulaire de monitorage au format ODK et un guide des actions à prendre durant le monitorage sur la base des observations de la supervision.
4. **Récupération des enfants (IT + Presi-Codesa) :**
   - Les ITs doivent collaborer avec les Presi-Codesa pour déterminer les quotas d'enfants que chaque relais communautaire doit ramener au site de vaccination
   - Ils doivent faire le suivi des quotas d'enfants à vacciner sur la base des jetons jaunes distribués et de la fiche de suivi des distributions des jetons jaunes aux enfants zéros dose et insuffisamment vaccinés par les relais et demander aux relais communautaires de ramener les enfants qui ne se présentent pas au site de vaccination.
5. **Chaine de communication :**
   - La première personne à contacter en cas de problèmes est l'IT pour les vaccinateurs et les encodeurs et le Presi-Codesa pour les relais communautaires.
6. **Circuit de collecte de données :**
   - Au niveau des sites de vaccination :
     5. Les jetons jaunes doivent être collectés par l'équipe
     6. Les jetons bleus et les cartes CPS sont remis aux mamans/accompagnateurs
     7. La saisie des fiches de provenance et des fiches de pointage sur papier et dans le téléphone se fait tous les jours au site même de vaccination
     8. Chaque équipe aura un numéro d'équipe (Liste des équipes avec leur codes)
     9. La fiches synthèses des fiches de pointage et de provenance est complétée par l'équipe chaque jour à la fin de la journée.

- Au niveau de l'aire :
    10. L'IT récupère les jetons jaunes
    11. Les fiches synthèses des fiches de pointage et de provenance sont envoyées chaque jour à l'IT pour une synthèse journalière.
    12. Le gestionnaire de données commence la saisie des fiches synthèses des fiches de pointage et de provenance dans la base de données dès le premier jour de la campagne

7. **Utilisation des téléphones :**
    - Chaque équipe aura un téléphone pour entrer les informations de la fiche de provenance et de la fiche de pointage
    - Chaque superviseur aura un téléphone pour entrer les informations du Formulaire de monitorage.
    - L'utilisation des téléphones est strictement réservée à la collecte de données pour l'IPVS. Il est strictement interdit de s'en servir pour des raisons personnelles (prise de photos, téléchargement d'application etc.).
    - Se référer au Guide pour l'utilisation des téléphones pour former les agents sur l'utilisation des téléphones
    - Une fois que la saisie des données est finale, il n'est plus possible de corriger/visualiser le contenu de la fiche entrée dans le téléphone. Les ITs qui désirent vérifier le contenu des fiches rentrées dans le téléphone devront contacter GRID3 pour vérifier la possibilité d'extraire des données pour analyse.
    - Contacter l'équipe GRID3 pour tout problème lie à l'utilisation des téléphones.

V. Actions à mener après la campagne :

- Analyse de la performance de la campagne et présentation des résultats aux acteurs impliqués dans l'IPVS.
- Récupération de toutes les fiches papier dans les sites par l'IT pour les ramener à GRID 3 lors de l'évaluation de l'IPVS.

VI. Récapitulatif des outils utilisés pour l'IPVS :

L'utilisation des outils (prise de décision, collecte de données etc.) doit être revue durant la formation avec des séances de pratique pour assurer une bonne assimilation :

- Acte d'engagement pour le transport (format papier)
- Antigènes selon l'âge (format papier)
- Cahier de vaccination (format papier)
- Calendrier d'accompagnement (format papier et électronique)
- Calendrier vaccinal (format papier)
- Cartes CPS (format papier)
- Cartes de regroupement des villages (format papier)
- Cartographie des mouvements des équipes (format papier)

- Circuit de Vaccination (format papier)
- Formulaire de monitorage (format ODK)
- Fiche de pointage (format papier et ODK)
- Fiche de provenance (format papier et ODK)
- Fiche de suivi des distributions des jetons jaunes aux enfants zéros dose et insuffisamment vaccinés par les relais (format papier)
- Fiche synthèse des fiches de pointage et de provenance (format papier)
- Fiches de dénombrement de 2022 (format papier)
- Guide d'âge (format papier)
- Guide des actions à prendre durant le monitorage (format papier)
- Guide pour l'utilisation des téléphones (format papier)
- Jeton bleu (format papier)
- Jeton jaune (format papier)
- Liste des équipes avec leurs codes (format papier et électronique)
- Liste des membres des équipes de vaccination (format papier et électronique)
- Liste de relais communautaires reconduits pour la phase 2 (format papier et électronique)
- Liste des enfants à récupérer (format papier)
- Liste des localités [fiches vertes (Kindu), oranges (Kibombo)] (format papier)

Manuel de formation des coordonnateurs de formation des vaccinateurs et des encodeurs.

Sections du Manuel de formation des coordonnateurs des aires de santé, des superviseurs d'axes et des Presi-Codesa à partager avec les vaccinateurs et les encodeurs

- Section II : Le contexte de l'IPVS :
- Section III : Actions à mener avant la campagne :
  - Section III – 2 : Plan de déploiement des équipes (IT)
  - Section III – 3 : Formation des vaccinateurs, encodeurs et relais communautaires (IT, Presi-Codesa, agents du bureau central de la zone
  - Section III – 4 : Sites de stockage (IT)
  - Section III – 5 : Préfinancement des équipes pour les zones à accès difficile (IT)
  - Section III – 6 : Modalités de paiement
- Section IV : Actions à mener pendant la campagne
- Section V : Actions à mener après la campagne
- Section VI : Récapitulatif des outils utilisés pour l'IPVS
  - Acte d'engagement pour le transport (format papier)
  - Antigènes selon l'âge (format papier)
  - Cahier de vaccination (format papier)
  - Calendrier vaccinal (format papier)
  - Cartes CPS (format papier)
  - Cartographie des mouvements des équipes (format papier)
  - Circuit de Vaccination (format papier)
  - Fiche de pointage (format papier et ODK)
  - Fiche de provenance (format papier et ODK)
  - Fiche synthèse des fiches de pointage et de provenance (format papier)
  - Guide d'âge (format papier)
  - Guide pour l'utilisation des téléphones (format papier)
  - Jeton bleu (format papier)
  - Liste des équipes avec leurs codes (format papier et électronique)
  - Liste des localités [fiches vertes (Kindu), oranges (Kibombo)] (format papier)

Manuel de formation des coordonnateurs de formation des relais communautaires.

Sections du Manuel de formation des coordonnateurs des aires de santé, des superviseurs d'axes et des Presi-Codesa à partager avec les relais communautaires

- Section II : Le contexte de l'IPVS :
- Section III : Actions à mener avant la campagne :
  - Section III – 1 : Sensibilisation (IT + Presi-Codesa)
  - Section III – 3 : Formation des vaccinateurs, encodeurs et relais communautaires (IT, Presi-Codesa, agents du bureau central de la zone
- Section IV : Actions à mener pendant la campagne
  - Section IV – 3 : Supervision
  - Section IV – 4 : Récupération des enfants (IT + Presi-Codesa)
  - Section IV – 5 : Chaine de communication
- Section V : Actions à mener après la campagne
- Section VI : Récapitulatif des outils utilisés pour l'IPVS
  - Calendrier vaccinal (format papier)
  - Fiche de suivi des distributions des jetons jaunes aux enfants zéros dose et insuffisamment vaccinés par les relais (format papier)
  - Fiches de dénombrement de 2022 (format papier)
  - Guide d'âge (format papier)
  - Jeton jaune (format papier)
  - Liste des enfants à récupérer (format papier)
