# Supplementary material for: Reaching Never- and Incompletely-Vaccinated Children with Routine Immunization: A Proof-of-Concept Activity Using Geo-Referenced Microplans in Two Health Zones in Maniema Province, Democratic Republic of the Congo
Source: Vaccines (Basel). 2026 Feb 13;14(2):175. doi: 10.3390/vaccines14020175 (PMC12945245; doi:10.3390/vaccines14020175)
Supplement: Supplementary file 1 [file vaccines-14-00175-s001.zip › Tools_Alleman_Maniema_10Feb2026.zip!/I2_Tool_S9B_Maniema_PIRI_TrainingMaterials_English.pdf]

TRAINING OF STAKEHOLDORS INVOLVED IN THE SECOND ROUND OF PERIODIC INTENSIFICATION OF ROUTINE IMMUNIZATION (PIRI)

November 25–26 to December 1–2, 2023

Training manual for health area coordinators, health area axis supervisors, and Presi-Codesa.

Note on the source of tools referenced in the manual:

- Tool developed or adapted by the CDC/GRID3 team
- Tool developed by the health center / health zone management team

I. Types of training to be conducted:

Three types of training grouping actors with similar responsibilities:

Training	Target (people to train)	Duration	Paid
Training of health area coordinators, axis supervisors, and Presi-Codesa	Health Facility In-Charge (Nurse) – hereafter referred to as IT, Presidents of the CODESA (Health Area Development Committees) – hereafter referred to as Presi-Codesa, and central health zone staff	1 day	Yes
Training of vaccinators and data clerks	Deputy Health Facility In-Charge (Nurse) – hereafter referred to as ITAs, nurses, and data clerks	1 day	No
Training of community health workers	Community health workers (mobilizers, children trackers)	1 day	No

Three training manuals will be available and will serve as a reminder of activities to complete for the PIRIs:

- Manual for coordinators, supervisors, and Presi-Codesa
- Manual for vaccinators and data clerks
- Manual for community health workers.

II. PIRI context:

1. **Target Age group**:

   Target for the PIRIs include children aged 0–59 months who are zero-dose or incompletely vaccinated based on vaccines children of similar age should have received.

2. **Expected number of children per locality:**

   To identify the number of children expected in each locality, please use estimates from the locality lists (green sheets for Kindu, orange for Kibombo).

3. **Vaccines and Vaccination Calendar:**

   The goal is to provide eight antigens: BCG, OPV, Penta, PCV13, ROTA, IPV, Measles, Yellow Fever. Please use the "Antigens by Age" chart, the Age guide, and the Vaccination Calendar to guide vaccine administration.

III. Tasks to complete before the campaign:

1. **Community awareness (IT + Presi-Codesa):**

1.1. Selection of community workers:

It is recommended to use the lists of community health workers renewed for Phase 2 based on performance during Phase 1 and to designate town criers for each vaccination team (2 town criers per team in Kindu and 2 town criers per village/neighborhood in Kibombo).

The IT must make the communication plan available to the Presi-Codesa.

1.2. Management of Yellow Tokens:

Provide a sufficient number of Yellow Tokens to the community health workers (plan for more than the target number of children to avoid shortages) and ask the Presi-Codesa to monitor the distribution of the tokens.

1.3. Children Tracing and Referring:

- Community health workers will use the Tracking sheet for the distribution of yellow tokens to zero-dose and under-vaccinated children by community health workers to provide reliable information on the households visited and the number of target children identified.
- Identify all children who need to be vaccinated, based on their Vaccination Cards, mothers' testimonies, 2022 Child Enumeration Forms, the Defaulter Tracking List available in health centers, etc.
- Inform households about vaccination sessions: remind them of the dates and locations of the vaccination sites.

1.4. Yellow Tokens Completion:

Ensure that the Yellow Tokens are properly filled out (child's name, location, gender, age, vaccination status, vaccination site, the date the Yellow Token was issued, and the name of the community health worker).

1.5. Supervision by Presi-Codesa:

The Presi-Codesa must be involved in selecting community health workers, managing and supervising these workers in the field, and distributing the Yellow Tokens. They must ensure that town criers go through the streets around the vaccination sites to inform people about the vaccination campaign and the location of the vaccination sites. They must also ensure that the selected individuals are familiar with their street/neighborhood, are available for the entire duration of the activity, and are trusted by the community.

1.6. Payment:

Payment will be made via mobile money, which requires updating and transmitting the lists of people to be paid.

The Presi-Codesa will receive $10 per person for the entire duration of the activity, the community health workers will receive $8 per person for the entire duration of the activity, and the town criers will receive $5 per person for the entire duration of the activity.

2. Deployment of vaccination teams (IT)

2.1. Selection of vaccinators and data entry clerks:

2.1.1. Vaccinators:

- The selection process should prioritize individuals who are familiar with routine vaccination and assessing vaccination status by questioning mothers or consulting the Yellow Tokens, who are able to administer vaccines, and who are proficient in filling out the Blue Tokens, Vaccination Cards, Child Provenance Form, Vaccination Tally Sheet, and the Vaccination Tally and Child Provenance Summary Form. Preference should also be given to individuals who are credible and available for the entire duration of the activity.
- The first vaccinator must communicate the vaccines to be administered to the second vaccinator using a piece of paper.
  Note: The Child Provenance Form is filled out by vaccinator 1. The Vaccination Tally Sheet is filled out by vaccinator 2.

2.1.2. Data entry clerk:

- The selection process should prioritize those who are able to understand the Child Provenance Form and Vaccination Tally Sheet, enter the data from these sheets into the phone, and who are capable of completing the Vaccination Tally and Child Provenance Summary Form, as well as entering information in the Vaccination Register.

Note: The Child Provenance Form and the Vaccination Tally Sheet are filled out by the vaccinators, but the data entry clerk must also be familiar with filling out these two forms in order to correctly enter the information into the phone.

- The data entry clerk must keep the Yellow Tokens.

2.2. Movement of Vaccination Teams Between Mobile Sites:

2.2.1. Review the Mobile Vaccination Site Deployment Map:

- Team deployments will be based on the map of mobile vaccination sites developed during micro-planning.
- It will be necessary to ensure that community health workers are familiar with the map of mobile vaccination sites and the schedule of team deployments in order to provide the correct information to the population regarding vaccination sites.

2.2.2. Comply with the map of mobile vaccination sites:

Ensure that the teams, with all their members present, are present at all the planned sites for each of the scheduled vaccination days.

2.2.3. Prioritization of remote sites:

Instruct the teams assigned to remote or difficult-to-access locations to begin vaccination sessions at the furthest sites and work their way back to the nearest sites to ensure they reach as many children as possible living in these areas.

2.2.4. Tools:

- Village Grouping Maps: maps showing the grouping of villages (500 m radius in urban areas and 5 km in rural areas) and health centers with an operational cold chain.
- Lists of vaccination team members (vaccinators and data entry clerks).
- List of vaccination teams with their codes: list of vaccination teams according to the microplan.

3. Training of vaccinators, data entry clerks and community health workers (IT, Presi-Codesa, central zone office staff)

3.1. Training Schedule:

Train the participants through practical sessions in households or at simulation sites.

For community health workers, provide training sufficiently in advance to conduct effective awareness campaigns and properly identify the children to be vaccinated in the community (allow at least 3 days for identifying the children and 2 days for follow-up visits).

At the end of the training:

- Provide vaccinators and data entry clerks with the Vaccinator and Data Entry Personnel Training Manual.
- Provide community health workers with the Community Health Worker Training Manual.

3.2. Supportive Supervision Schedule:

Comply with the Supportive Supervision Schedule developed with the central health zone office with the support of GRID3.

4. Storage sites (IT):

- Identify temporary relay storage points and establish a resupply plan with support from the Health Zone central office.
- Plan for supply in advance of the start of vaccination activities.
- Communicate the location of the storage sites to the vaccination teams.

5. Advance Funding for Teams Operating in Hard-to-Reach Areas (IT):

- The ITs are responsible for ensuring that advance funds are used to reach remote and/or hard-to-reach sites and vaccinate children.
- Team members assigned to remote and/or hard-to-reach sites must sign a Transport Commitment Form.

6. Payment methods:

- Payment will be made via mobile money and requires updating the List of vaccination team members (vaccinators and data entry clerks).
- Vaccinators and data entry clerks will receive $5 per person, per day, for 5 days, based on performance evaluation.

IV. Tasks to complete during the campaign:

1. Vaccination techniques (IT):

Comply with Expanded Program on Immunization (EPI) standards for vaccine storage, preparation, administration, waste management, and management of Adverse Events Following Immunization (AEFI).

2. Organization of the vaccination site (IT):

- Each team consists of three members (two vaccinators and one data entry clerk).
- Site organization follows the layout outlined in the Vaccination Circuit tool.
- The team must obtain tables and chairs, requesting assistance from the community if needed, to properly organize the vaccination site.
- The responsibilities of the vaccination team members:
    1. The responsibilities of Vaccinator 1: verify the children's vaccination status, communicate the vaccines to be administered to the second vaccinator on a piece of paper, fill in the general information on the Vaccination Cards and Blue Tokens, fill out the Child Provenance Form on paper as the children arrive, explain the side effects and what to do in case of adverse events following immunization (AEFI), invite the children for the next vaccination session, manage waste, ensure that the data

entry clerk has entered the data into the phone, and complete the Vaccination Tally and Child Provenance Summary Form with the rest of the team at the end of the day.

IMPORTANT NOTE:

- **The locality of the children on the Vaccination Tally Sheet is the locality of the child's place of residence/origin, not the locality of the vaccination site. Use the List of Localities provided to the team to record the correct locality.**
- **Information for managing adverse events following immunization (AEFIs) can be found on the back of the Vaccination Tally Sheet.**

2. The responsibilities of the Vaccinator 2: Validate children's vaccination status, prepare and administer vaccines, fill in vaccination information on the Vaccination Cards and the Blue Tokens, and on the Vaccination Tally Sheet.
3. The responsibilities of the data entry clerk:
    - During the session: fill out the Child Provenance Form on your phone during downtime (times when the Vaccination Circuit does not have children to vaccinate), invite the children for the next PIRI, and fill in the vaccination information in the Vaccination Register.
    - At the end of the session: finish entering the Child Provenance Form information on the phone and enter the Vaccination Tally Sheet information on the phone.
    - Ensure that the Child Provenance Form and Vaccination Tally Sheet information is entered into the phone at the vaccination site and not elsewhere.
    -
4. The team's responsibilities:
    - Ensure that the Child Provenance Form and Vaccination Tally Sheet information is entered into the phone at the vaccination site and not elsewhere.

3. **Supervision:**
    - The Presi-Codesa will supervise community health workers and town criers using a Tracking sheet for the distribution of yellow tokens to zero-dose and under-vaccinated children by community health workers.
    - The IT and central zone office agents will supervise the vaccinators and data entry personnel.
    - All supervisors (except for the Presi-Codesa) will use an ODK Monitoring Form and a guide outlining the actions to be taken during monitoring based on observations made during supervision (Guide for Actions During Monitoring).
4. **Referring the children (IT + Presi-Codesa):**
    - The ITs must collaborate with the Presi-Codesa to determine the quotas of children that each community health worker must bring to the vaccination site.
    - They must monitor the quotas of children to be vaccinated based on the Yellow Tokens distributed and the Tracking sheet for the distribution of yellow tokens to zero-dose and under-vaccinated children by community health workers (paper format) and ask the

community health workers to bring in the children who do not show up at the vaccination site.

5. Communication chain:
   - The first point of contact in case of problems is the IT for vaccinators and data entry personnel, and Presi-Codesa for community health workers.
6. Data collection circuit:
   - At the vaccination sites:
     5. The Yellow Tokens must be collected by the team.
     6. The Blue Tokens and Vaccination Cards are given to the mothers/caregivers.
     7. The entry of the information on Child Provenance Forms and Vaccination Tally Sheet, both on paper and on mobile phones, is done daily at the vaccination site
     8. Each team will have a team number (List of teams with their codes)
     9. The Vaccination Tally and Child Provenance Summary Form is completed by the team every day at the end of the day.
   - At the area level:
     10. IT retrieves the Yellow Tokens.
     11. The Vaccination Tally and Child Provenance Summary Forms are sent daily to the IT for a daily summary.
     12. The data manager begins entering the Vaccination Tally and Child Provenance Summary Forms into the database from the first day of the campaign.
7. Using GRID3 mobile phones:
   - Each team will have a phone to enter the information from the Vaccination Tally Sheet and the Child Provenance Form.
   - Each supervisor will have a phone to enter the information from the Monitoring Form.
   - The use of mobile phones is strictly reserved for data collection for the PIRI project. Using them for personal reasons (taking photos, downloading applications, etc.) is strictly prohibited.
   - Refer to the GRID3 Mobile Phone User Guide to train agents on how to use the phones.
   - Once data entry is complete, it is no longer possible to correct or view the content of the form entered on the phone. IT who wish to verify the content of the forms entered on the phone must contact GRID3 to inquire about the possibility of extracting the data for analysis.
   - Contact the GRID 3 team for any problems related to using the phones.

V. Tasks to complete after the campaign:

- Analysis of campaign performance and presentation of results to stakeholders involved in the PIRIs.
- Collection of all paper forms from the sites by the ITs to bring them back to GRID 3 during the PIRI evaluation.

VI. Summary of the tools used for the PIRIs:

The use of the tools (decision-making, data collection, etc.) must be reviewed during the training with practical sessions to ensure proper understanding and assimilation:

- 2022 Child Enumeration Forms (paper format)
- Age guide (paper format)
- Antigens by age (paper format)
- Blue Tokens (paper format)
- Child Provenance Form (paper format and ODK)
- Defaulter Tracking List (paper format)
- GRID3 Mobile Phone User Guide (paper format)
- Guide for Actions During Monitoring (paper format)
- List of community health workers renewed for Phase 2 (paper and electronic format)
- List of localities [green sheets (Kindu), orange sheets (Kibombo)] (paper format)
- List of vaccination team members (paper and electronic format)
- List of vaccination teams with their codes (paper and electronic format)
- Mobile Vaccination Site Deployment Map (paper format)
- Monitoring form (ODK format)
- Supportive Supervision Schedule (paper and electronic formats)
- Tracking sheet for the distribution of yellow tokens to zero-dose and under-vaccinated children by community health workers (paper format)
- Transport Commitment Form (paper format)
- Vaccination Calendar (paper format)
- Vaccination Cards (paper format)
- Vaccination Circuit (paper form)
- Vaccination Register (paper format)
- Vaccination Tally and Child Provenance Summary Form (paper format)
- Vaccination Tally Sheet (paper format and ODK)
- Village Grouping Maps (paper format)
- Yellow Token (paper format)

Training manual for vaccinators and data entry clerks.

Sections of the Training manual for health area coordinators, health area axis supervisors, and Presi-Codesa to be shared with vaccinators and data entry clerks.

- Section II: PIRI context:
- Section III: Tasks to complete before the campaign.
    - Section III – 2: Deployment of vaccination teams (IT).
    - Section III – 3: Training of vaccinators, data entry clerks and community health workers (IT, Presi-Codesa, central zone office staff).
    - Section III – 4: Storage sites (IT).
    - Section III – 5: Advance Funding for Teams Operating in Hard-to-Reach Areas (IT).
    - Section III – 6: Payment methods.
- Section IV: Tasks to complete during the campaign.
- Section V: Tasks to complete after the campaign.
- Section VI: Summary of the tools used for the PIRIs.
    - Age guide (paper format).
    - Antigens by age (paper format).
    - Blue Tokens (paper format).
    - Child Provenance Form (paper format and ODK).
    - GRID3 Mobile Phone User Guide (paper format).
    - List of localities [green sheets (Kindu), orange sheets (Kibombo)] (paper format).
    - List of vaccination teams with their codes (paper and electronic format).
    - Mobile Vaccination Site Deployment Map (paper format).
    - Transport Commitment Form (paper format).
    - Vaccination Calendar (paper format).
    - Vaccination Cards (paper format).
    - Vaccination Circuit (paper form).
    - Vaccination Register (paper format).
    - Vaccination Tally and Child Provenance Summary Form (paper format).
    - Vaccination Tally Sheet (paper format and ODK).

Training manual for community health workers.

Sections of the Training manual for health area coordinators, health area axis supervisors, and Presi-Codesa to be shared with Community Health Workers.

- Section II: PIRI context.
- Section III: Tasks to complete before the campaign.
    - Section III – 1: Community awareness (IT + Presi-Codesa).
    - Section III – 3: Training of vaccinators, data entry clerks and community health workers (IT, Presi-Codesa, central zone office staff).
- Section IV: Tasks to complete during the campaign.
    - Section IV – 3: Supervision.
    - Section IV – 4: Referring the children (IT + Presi-Codesa).
    - Section IV – 5: Communication chain.
- Section V: Tasks to complete after the campaign.
- Section VI: Summary of the tools used for the PIRIs.
    - Vaccination Cards (paper format).
    - Tracking sheet for the distribution of yellow tokens to zero-dose and under-vaccinated children by community health workers (paper format).
    - 2022 Child Enumeration Forms (paper format).
    - Age guide (paper format).
    - Yellow Token (paper format).
    - Defaulter Tracking List (paper format).
