# Supplementary material for: Reaching Never- and Incompletely-Vaccinated Children with Routine Immunization: A Proof-of-Concept Activity Using Geo-Referenced Microplans in Two Health Zones in Maniema Province, Democratic Republic of the Congo
Source: Vaccines (Basel). 2026 Feb 13;14(2):175. doi: 10.3390/vaccines14020175 (PMC12945245; doi:10.3390/vaccines14020175)
Supplement: Supplementary file 1 [file vaccines-14-00175-s001.zip › Tools_Alleman_Maniema_10Feb2026.zip!/J1_Tool_S10A_Maniema_PIRI_TallySheet_French.pdf]

Fiche de pointage:

Activités de vaccination, Projet de renforcement de la vaccination de routine chez des enfants non-vaccinés ou insuffisamment vaccinés à l'aide de micro plans validés dans les zones de santé à haut risque de transmission de la polio et de la rougeole, Kindu/Kibombo, Maniema, RDC

1. Zone de Santé :	2. Aire de Santé :	3. Noms des membres de l'équipe de vaccination :	4. Code Equipe :
5. Date de la séance (Tarehe ya siku ya chanjo) :	6. Nom du poste de vaccination (Nom de l'établissement de soin de santé ou de la localité du poste si hors d'une établissement de soin de santé) (Jina ya fasi yenye ku fanyika chanjo) :		
7. Le poste de vaccination, est-il une établissement de soin de santé privée? (Faites un X à côté de la réponse) : _____Oui ____Non	8. Type de stratégie de vaccination (Faites un X à côté de la stratégie) : Fasi ya chanjo (Tiya musalaba nyuma ya fasi ya chanjo, kama ni ku baraza ya afya ao fasi ingine) : _____Fixe _____Avancé _____Mobile	9. Numéro de passage (Faites un X à côté du passage actuel) : _____1er _____2e _____3e	

0-11 mois: Faites un X sur le numéro; M=Masculin, F=Féminin

BCG	M	1	2	3	4	5	6	7	8	9	10	11	12	13	14	15	16	17	18	19	20
		21	22	23	24	25	26	27	28	29	30	31	32	33	34	35	36	37	38	39	40
	F	1	2	3	4	5	6	7	8	9	10	11	12	13	14	15	16	17	18	19	20
		21	22	23	24	25	26	27	28	29	30	31	32	33	34	35	36	37	38	39	40
VPOb(0)	M	1	2	3	4	5	6	7	8	9	10	11	12	13	14	15	16	17	18	19	20
		21	22	23	24	25	26	27	28	29	30	31	32	33	34	35	36	37	38	39	40
	F	1	2	3	4	5	6	7	8	9	10	11	12	13	14	15	16	17	18	19	20
		21	22	23	24	25	26	27	28	29	30	31	32	33	34	35	36	37	38	39	40
VPOb(1)	M	1	2	3	4	5	6	7	8	9	10	11	12	13	14	15	16	17	18	19	20
		21	22	23	24	25	26	27	28	29	30	31	32	33	34	35	36	37	38	39	40
	F	1	2	3	4	5	6	7	8	9	10	11	12	13	14	15	16	17	18	19	20
		21	22	23	24	25	26	27	28	29	30	31	32	33	34	35	36	37	38	39	40
PENTA(1)	M	1	2	3	4	5	6	7	8	9	10	11	12	13	14	15	16	17	18	19	20
		21	22	23	24	25	26	27	28	29	30	31	32	33	34	35	36	37	38	39	40
	F	1	2	3	4	5	6	7	8	9	10	11	12	13	14	15	16	17	18	19	20
		21	22	23	24	25	26	27	28	29	30	31	32	33	34	35	36	37	38	39	40
PCV13(1)	M	1	2	3	4	5	6	7	8	9	10	11	12	13	14	15	16	17	18	19	20
		21	22	23	24	25	26	27	28	29	30	31	32	33	34	35	36	37	38	39	40
	F	1	2	3	4	5	6	7	8	9	10	11	12	13	14	15	16	17	18	19	20
		21	22	23	24	25	26	27	28	29	30	31	32	33	34	35	36	37	38	39	40
ROTA(1)	M	1	2	3	4	5	6	7	8	9	10	11	12	13	14	15	16	17	18	19	20
		21	22	23	24	25	26	27	28	29	30	31	32	33	34	35	36	37	38	39	40
	F	1	2	3	4	5	6	7	8	9	10	11	12	13	14	15	16	17	18	19	20
		21	22	23	24	25	26	27	28	29	30	31	32	33	34	35	36	37	38	39	40
VPOb(2)	M	1	2	3	4	5	6	7	8	9	10	11	12	13	14	15	16	17	18	19	20
		21	22	23	24	25	26	27	28	29	30	31	32	33	34	35	36	37	38	39	40
	F	1	2	3	4	5	6	7	8	9	10	11	12	13	14	15	16	17	18	19	20
		21	22	23	24	25	26	27	28	29	30	31	32	33	34	35	36	37	38	39	40
PENTA(2)	M	1	2	3	4	5	6	7	8	9	10	11	12	13	14	15	16	17	18	19	20
		21	22	23	24	25	26	27	28	29	30	31	32	33	34	35	36	37	38	39	40
	F	1	2	3	4	5	6	7	8	9	10	11	12	13	14	15	16	17	18	19	20
		21	22	23	24	25	26	27	28	29	30	31	32	33	34	35	36	37	38	39	40
PCV13(2)	M	1	2	3	4	5	6	7	8	9	10	11	12	13	14	15	16	17	18	19	20
		21	22	23	24	25	26	27	28	29	30	31	32	33	34	35	36	37	38	39	40
	F	1	2	3	4	5	6	7	8	9	10	11	12	13	14	15	16	17	18	19	20
		21	22	23	24	25	26	27	28	29	30	31	32	33	34	35	36	37	38	39	40
ROTA(2)	M	1	2	3	4	5	6	7	8	9	10	11	12	13	14	15	16	17	18	19	20
		21	22	23	24	25	26	27	28	29	30	31	32	33	34	35	36	37	38	39	40
	F	1	2	3	4	5	6	7	8	9	10	11	12	13	14	15	16	17	18	19	20
		21	22	23	24	25	26	27	28	29	30	31	32	33	34	35	36	37	38	39	40
VPOb(3)	M	1	2	3	4	5	6	7	8	9	10	11	12	13	14	15	16	17	18	19	20
		21	22	23	24	25	26	27	28	29	30	31	32	33	34	35	36	37	38	39	40
	F	1	2	3	4	5	6	7	8	9	10	11	12	13	14	15	16	17	18	19	20
		21	22	23	24	25	26	27	28	29	30	31	32	33	34	35	36	37	38	39	40
PENTA(3)	M	1	2	3	4	5	6	7	8	9	10	11	12	13	14	15	16	17	18	19	20
		21	22	23	24	25	26	27	28	29	30	31	32	33	34	35	36	37	38	39	40
	F	1	2	3	4	5	6	7	8	9	10	11	12	13	14	15	16	17	18	19	20
		21	22	23	24	25	26	27	28	29	30	31	32	33	34	35	36	37	38	39	40
PCV13(3)	M	1	2	3	4	5	6	7	8	9	10	11	12	13	14	15	16	17	18	19	20
		21	22	23	24	25	26	27	28	29	30	31	32	33	34	35	36	37	38	39	40
	F	1	2	3	4	5	6	7	8	9	10	11	12	13	14	15	16	17	18	19	20
		21	22	23	24	25	26	27	28	29	30	31	32	33	34	35	36	37	38	39	40
ROTA(3)	M	1	2	3	4	5	6	7	8	9	10	11	12	13	14	15	16	17	18	19	20
		21	22	23	24	25	26	27	28	29	30	31	32	33	34	35	36	37	38	39	40
	F	1	2	3	4	5	6	7	8	9	10	11	12	13	14	15	16	17	18	19	20
		21	22	23	24	25	26	27	28	29	30	31	32	33	34	35	36	37	38	39	40
VPI(1)	M	1	2	3	4	5	6	7	8	9	10	11	12	13	14	15	16	17	18	19	20
		21	22	23	24	25	26	27	28	29	30	31	32	33	34	35	36	37	38	39	40
	F	1	2	3	4	5	6	7	8	9	10	11	12	13	14	15	16	17	18	19	20
		21	22	23	24	25	26	27	28	29	30	31	32	33	34	35	36	37	38	39	40
VAR(1)	M	1	2	3	4	5	6	7	8	9	10	11	12	13	14	15	16	17	18	19	20
		21	22	23	24	25	26	27	28	29	30	31	32	33	34	35	36	37	38	39	40
	F	1	2	3	4	5	6	7	8	9	10	11	12	13	14	15	16	17	18	19	20
		21	22	23	24	25	26	27	28	29	30	31	32	33	34	35	36	37	38	39	40
VPI(2)	M	1	2	3	4	5	6	7	8	9	10	11	12	13	14	15	16	17	18	19	20
		21	22	23	24	25	26	27	28	29	30	31	32	33	34	35	36	37	38	39	40
	F	1	2	3	4	5	6	7	8	9	10	11	12	13	14	15	16	17	18	19	20
		21	22	23	24	25	26	27	28	29	30	31	32	33	34	35	36	37	38	39	40
VAA	M	1	2	3	4	5	6	7	8	9	10	11	12	13	14	15	16	17	18	19	20
		21	22	23	24	25	26	27	28	29	30	31	32	33	34	35	36	37	38	39	40
	F	1	2	3	4	5	6	7	8	9	10	11	12	13	14	15	16	17	18	19	20
		21	22	23	24	25	26	27	28	29	30	31	32	33	34	35	36	37	38	39	40

12-23 mois: Faites un X sur le numéro; M=Masculin, F=Féminin

VPOb(1)	M	1	2	3	4	5	6	7	8	9	10	11	12	13	14	15	16	17	18	19	20
		21	22	23	24	25	26	27	28	29	30	31	32	33	34	35	36	37	38	39	40
	F	1	2	3	4	5	6	7	8	9	10	11	12	13	14	15	16	17	18	19	20
		21	22	23	24	25	26	27	28	29	30	31	32	33	34	35	36	37	38	39	40
PENTA(1)	M	1	2	3	4	5	6	7	8	9	10	11	12	13	14	15	16	17	18	19	20
		21	22	23	24	25	26	27	28	29	30	31	32	33	34	35	36	37	38	39	40
	F	1	2	3	4	5	6	7	8	9	10	11	12	13	14	15	16	17	18	19	20
		21	22	23	24	25	26	27	28	29	30	31	32	33	34	35	36	37	38	39	40
PCV13(1)	M	1	2	3	4	5	6	7	8	9	10	11	12	13	14	15	16	17	18	19	20
		21	22	23	24	25	26	27	28	29	30	31	32	33	34	35	36	37	38	39	40
	F	1	2	3	4	5	6	7	8	9	10	11	12	13	14	15	16	17	18	19	20
		21	22	23	24	25	26	27	28	29	30	31	32	33	34	35	36	37	38	39	40
ROTA(1)	M	1	2	3	4	5	6	7	8	9	10	11	12	13	14	15	16	17	18	19	20
		21	22	23	24	25	26	27	28	29	30	31	32	33	34	35	36	37	38	39	40
	F	1	2	3	4	5	6	7	8	9	10	11	12	13	14	15	16	17	18	19	20
		21	22	23	24	25	26	27	28	29	30	31	32	33	34	35	36	37	38	39	40
VPOb(2)	M	1	2	3	4	5	6	7	8	9	10	11	12	13	14	15	16	17	18	19	20
		21	22	23	24	25	26	27	28	29	30	31	32	33	34	35	36	37	38	39	40
	F	1	2	3	4	5	6	7	8	9	10	11	12	13	14	15	16	17	18	19	20
		21	22	23	24	25	26	27	28	29	30	31	32	33	34	35	36	37	38	39	40
PENTA(2)	M	1	2	3	4	5	6	7	8	9	10	11	12	13	14	15	16	17	18	19	20
		21	22	23	24	25	26	27	28	29	30	31	32	33	34	35	36	37	38	39	40
	F	1	2	3	4	5	6	7	8	9	10	11	12	13	14	15	16	17	18	19	20
		21	22	23	24	25	26	27	28	29	30	31	32	33	34	35	36	37	38	39	40
PCV13(2)	M	1	2	3	4	5	6	7	8	9	10	11	12	13	14	15	16	17	18	19	20
		21	22	23	24	25	26	27	28	29	30	31	32	33	34	35	36	37	38	39	40
	F	1	2	3	4	5	6	7	8	9	10	11	12	13	14	15	16	17	18	19	20
		21	22	23	24	25	26	27	28	29	30	31	32	33	34	35	36	37	38	39	40
ROTA(2)	M	1	2	3	4	5	6	7	8	9	10	11	12	13	14	15	16	17	18	19	20
		21	22	23	24	25	26	27	28	29	30	31	32	33	34	35	36	37	38	39	40
	F	1	2	3	4	5	6	7	8	9	10	11	12	13	14	15	16	17	18	19	20
		21	22	23	24	25	26	27	28	29	30	31	32	33	34	35	36	37	38	39	40
VPOb(3)	M	1	2	3	4	5	6	7	8	9	10	11	12	13	14	15	16	17	18	19	20
		21	22	23	24	25	26	27	28	29	30	31	32	33	34	35	36	37	38	39	40
	F	1	2	3	4	5	6	7	8	9	10	11	12	13	14	15	16	17	18	19	20
		21	22	23	24	25	26	27	28	29	30	31	32	33	34	35	36	37	38	39	40
PENTA(3)	M	1	2	3	4	5	6	7	8	9	10	11	12	13	14	15	16	17	18	19	20
		21	22	23	24	25	26	27	28	29	30	31	32	33	34	35	36	37	38	39	40
	F	1	2	3	4	5	6	7	8	9	10	11	12	13	14	15	16	17	18	19	20
		21	22	23	24	25	26	27	28	29	30	31	32	33	34	35	36	37	38	39	40
ROTA(3)	M	1	2	3	4	5	6	7	8	9	10	11	12	13	14	15	16	17	18	19	20
		21	22	23	24	25	26	27	28	29	30	31	32	33	34	35	36	37	38	39	40
	F	1	2	3	4	5	6	7	8	9	10	11	12	13	14	15	16	17	18	19	20
		21	22	23	24	25	26	27	28	29	30	31	32	33	34	35	36	37	38	39	40
VPI(1)	M	1	2	3	4	5	6	7	8	9	10	11	12	13	14	15	16	17	18	19	20
		21	22	23	24	25	26	27	28	29	30	31	32	33	34	35	36	37	38	39	40
	F	1	2	3	4	5	6	7	8	9	10	11	12	13	14	15	16	17	18	19	20
		21	22	23	24	25	26	27	28	29	30	31	32	33	34	35	36	37	38	39	40
VAR(1)	M	1	2	3	4	5	6	7	8	9	10	11	12	13	14	15	16	17	18	19	20
		21	22	23	24	25	26	27	28	29	30	31	32	33	34	35	36	37	38	39	40
	F	1	2	3	4	5	6	7	8	9	10	11	12	13	14	15	16	17	18	19	20
		21	22	23	24	25	26	27	28	29	30	31	32	33	34	35	36	37	38	39	40
VPI(2)	M	1	2	3	4	5	6	7	8	9	10	11	12	13	14	15	16	17	18	19	20
		21	22	23	24	25	26	27	28	29	30	31	32	33	34	35	36	37	38	39	40
	F	1	2	3	4	5	6	7	8	9	10	11	12	13	14	15	16	17	18	19	20
		21	22	23	24	25	26	27	28	29	30	31	32	33	34	35	36	37	38	39	40
VAR(2)	M	1	2	3	4	5	6	7	8	9	10	11	12	13	14	15	16	17	18	19	20
		21	22	23	24	25	26	27	28	29	30	31	32	33	34	35	36	37	38	39	40
	F	1	2	3	4	5	6	7	8	9	10	11	12	13	14	15	16	17	18	19	20
		21	22	23	24	25	26	27	28	29	30	31	32	33	34	35	36	37	38	39	40
VAA	M	1	2	3	4	5	6	7	8	9	10	11	12	13	14	15	16	17	18	19	20
		21	22	23	24	25	26	27	28	29	30	31	32	33	34	35	36	37	38	39	40
	F	1	2	3	4	5	6	7	8	9	10	11	12	13	14	15	16	17	18	19	20
		21	22	23	24	25	26	27	28	29	30	31	32	33	34	35	36	37	38	39	40

24-59 mois: Faites un X sur le numéro; M=Masculin, F=Féminin

VPOb(1)	M	1	2	3	4	5	6	7	8	9	10	11	12	13	14	15	16	17	18	19	20
		21	22	23	24	25	26	27	28	29	30	31	32	33	34	35	36	37	38	39	40
	F	1	2	3	4	5	6	7	8	9	10	11	12	13	14	15	16	17	18	19	20
		21	22	23	24	25	26	27	28	29	30	31	32	33	34	35	36	37	38	39	40
PENTA(1)	M	1	2	3	4	5	6	7	8	9	10	11	12	13	14	15	16	17	18	19	20
		21	22	23	24	25	26	27	28	29	30	31	32	33	34	35	36	37	38	39	40
	F	1	2	3	4	5	6	7	8	9	10	11	12	13	14	15	16	17	18	19	20
		21	22	23	24	25	26	27	28	29	30	31	32	33	34	35	36	37	38	39	40
PCV13(1)	M	1	2	3	4	5	6	7	8	9	10	11	12	13	14	15	16	17	18	19	20
		21	22	23	24	25	26	27	28	29	30	31	32	33	34	35	36	37	38	39	40
	F	1	2	3	4	5	6	7	8	9	10	11	12	13	14	15	16	17	18	19	20
		21	22	23	24	25	26	27	28	29	30	31	32	33	34	35	36	37	38	39	40
VPOb(2)	M	1	2	3	4	5	6	7	8	9	10	11	12	13	14	15	16	17	18	19	20
		21	22	23	24	25	26	27	28	29	30	31	32	33	34	35	36	37	38	39	40
	F	1	2	3	4	5	6	7	8	9	10	11	12	13	14	15	16	17	18	19	20
		21	22	23	24	25	26	27	28	29	30	31	32	33	34	35	36	37	38	39	40
PENTA(2)	M	1	2	3	4	5	6	7	8	9	10	11	12	13	14	15	16	17	18	19	20
		21	22	23	24	25	26	27	28	29	30	31	32	33	34	35	36	37	38	39	40
	F	1	2	3	4	5	6	7	8	9	10	11	12	13	14	15	16	17	18	19	20
		21	22	23	24	25	26	27	28	29	30	31	32	33	34	35	36	37	38	39	40
VPOb(3)	M	1	2	3	4	5	6	7	8	9	10	11	12	13	14	15	16	17	18	19	20
		21	22	23	24	25	26	27	28	29	30	31	32	33	34	35	36	37	38	39	40
	F	1	2	3	4	5	6	7	8	9	10	11	12	13	14	15	16	17	18	19	20
		21	22	23	24	25	26	27	28	29	30	31	32	33	34	35	36	37	38	39	40
PENTA(3)	M	1	2	3	4	5	6	7	8	9	10	11	12	13	14	15	16	17	18	19	20
		21	22	23	24	25	26	27	28	29	30	31	32	33	34	35	36	37	38	39	40
	F	1	2	3	4	5	6	7	8	9	10	11	12	13	14	15	16	17	18	19	20
		21	22	23	24	25	26	27	28	29	30	31	32	33	34	35	36	37	38	39	40
VPI(1)	M	1	2	3	4	5	6	7	8	9	10	11	12	13	14	15	16	17	18	19	20
		21	22	23	24	25	26	27	28	29	30	31	32	33	34	35	36	37	38	39	40
	F	1	2	3	4	5	6	7	8	9	10	11	12	13	14	15	16	17	18	19	20
		21	22	23	24	25	26	27	28	29	30	31	32	33	34	35	36	37	38	39	40
VAR(1)	M	1	2	3	4	5	6	7	8	9	10	11	12	13	14	15	16	17	18	19	20
		21	22	23	24	25	26	27	28	29	30	31	32	33	34	35	36	37	38	39	40
	F	1	2	3	4	5	6	7	8	9	10	11	12	13	14	15	16	17	18	19	20
		21	22	23	24	25	26	27	28	29	30	31	32	33	34	35	36	37	38	39	40
VPI(2)	M	1	2	3	4	5	6	7	8	9	10	11	12	13	14	15	16	17	18	19	20
		21	22	23	24	25	26	27	28	29	30	31	32	33	34	35	36	37	38	39	40
	F	1	2	3	4	5	6	7	8	9	10	11	12	13	14	15	16	17	18	19	20
		21	22	23	24	25	26	27	28	29	30	31	32	33	34	35	36	37	38	39	40
VAR(2)	M	1	2	3	4	5	6	7	8	9	10	11	12	13	14	15	16	17	18	19	20
		21	22	23	24	25	26	27	28	29	30	31	32	33	34	35	36	37	38	39	40
	F	1	2	3	4	5	6	7	8	9	10	11	12	13	14	15	16	17	18	19	20
		21	22	23	24	25	26	27	28	29	30	31	32	33	34	35	36	37	38	39	40
VAA	M	1	2	3	4	5	6	7	8	9	10	11	12	13	14	15	16	17	18	19	20
		21	22	23	24	25	26	27	28	29	30	31	32	33	34	35	36	37	38	39	40
	F	1	2	3	4	5	6	7	8	9	10	11	12	13	14	15	16	17	18	19	20
		21	22	23	24	25	26	27	28	29	30	31	32	33	34	35	36	37	38	39	40
