# Supplementary material for: Reaching Never- and Incompletely-Vaccinated Children with Routine Immunization: A Proof-of-Concept Activity Using Geo-Referenced Microplans in Two Health Zones in Maniema Province, Democratic Republic of the Congo
Source: Vaccines (Basel). 2026 Feb 13;14(2):175. doi: 10.3390/vaccines14020175 (PMC12945245; doi:10.3390/vaccines14020175)
Supplement: Supplementary file 1 [file vaccines-14-00175-s001.zip › Tools_Alleman_Maniema_10Feb2026.zip!/J2_Tool_S10B_Maniema_PIRI_TallySheet_English.pdf]

Tally sheet:

Vaccination Activities 2023. Project to reinforce routine vaccination among children never vaccinated or incompletely vaccinated using validated microplans in health zones at high risk for transmission of measles and polio, Kindu and Kibombo, Maniema Province, Democratic Republic of the Congo

Health Zone:	Health Area:	Names of the members of the vaccination team:
Date of the session:	Name of the vaccination site (Name of the health facility or the locality of the session if held outside of a health facility:	
Is the vaccination site a private health facility (Mark an X next to the response) : _____Yes ____No	Type of vaccination strategy (Mark an X next to the strategy) : _____Fixed _____Advanced _____Mobile	Which PIRI? (Mark an X next to the ongoing PIRI) : _____1st _____2nd _____3rd

0-11 months: Mark an X on the number; M=Male, F=Female

BCG	M	1	2	3	4	5	6	7	8	9	10	11	12	13	14	15	16	17	18	19	20
		21	22	23	24	25	26	27	28	29	30	31	32	33	34	35	36	37	38	39	40
	F	1	2	3	4	5	6	7	8	9	10	11	12	13	14	15	16	17	18	19	20
		21	22	23	24	25	26	27	28	29	30	31	32	33	34	35	36	37	38	39	40
bOPV(0)	M	1	2	3	4	5	6	7	8	9	10	11	12	13	14	15	16	17	18	19	20
		21	22	23	24	25	26	27	28	29	30	31	32	33	34	35	36	37	38	39	40
	F	1	2	3	4	5	6	7	8	9	10	11	12	13	14	15	16	17	18	19	20
		21	22	23	24	25	26	27	28	29	30	31	32	33	34	35	36	37	38	39	40
bOPV(1)	M	1	2	3	4	5	6	7	8	9	10	11	12	13	14	15	16	17	18	19	20
		21	22	23	24	25	26	27	28	29	30	31	32	33	34	35	36	37	38	39	40
	F	1	2	3	4	5	6	7	8	9	10	11	12	13	14	15	16	17	18	19	20
		21	22	23	24	25	26	27	28	29	30	31	32	33	34	35	36	37	38	39	40
PENTA(1)	M	1	2	3	4	5	6	7	8	9	10	11	12	13	14	15	16	17	18	19	20
		21	22	23	24	25	26	27	28	29	30	31	32	33	34	35	36	37	38	39	40
	F	1	2	3	4	5	6	7	8	9	10	11	12	13	14	15	16	17	18	19	20
		21	22	23	24	25	26	27	28	29	30	31	32	33	34	35	36	37	38	39	40
PCV13(1)	M	1	2	3	4	5	6	7	8	9	10	11	12	13	14	15	16	17	18	19	20
		21	22	23	24	25	26	27	28	29	30	31	32	33	34	35	36	37	38	39	40
	F	1	2	3	4	5	6	7	8	9	10	11	12	13	14	15	16	17	18	19	20
		21	22	23	24	25	26	27	28	29	30	31	32	33	34	35	36	37	38	39	40
ROTA(1)	M	1	2	3	4	5	6	7	8	9	10	11	12	13	14	15	16	17	18	19	20
		21	22	23	24	25	26	27	28	29	30	31	32	33	34	35	36	37	38	39	40
	F	1	2	3	4	5	6	7	8	9	10	11	12	13	14	15	16	17	18	19	20
		21	22	23	24	25	26	27	28	29	30	31	32	33	34	35	36	37	38	39	40
bOPV(2)	M	1	2	3	4	5	6	7	8	9	10	11	12	13	14	15	16	17	18	19	20
		21	22	23	24	25	26	27	28	29	30	31	32	33	34	35	36	37	38	39	40
	F	1	2	3	4	5	6	7	8	9	10	11	12	13	14	15	16	17	18	19	20
		21	22	23	24	25	26	27	28	29	30	31	32	33	34	35	36	37	38	39	40
PENTA(2)	M	1	2	3	4	5	6	7	8	9	10	11	12	13	14	15	16	17	18	19	20
		21	22	23	24	25	26	27	28	29	30	31	32	33	34	35	36	37	38	39	40
	F	1	2	3	4	5	6	7	8	9	10	11	12	13	14	15	16	17	18	19	20
		21	22	23	24	25	26	27	28	29	30	31	32	33	34	35	36	37	38	39	40
PCV13(2)	M	1	2	3	4	5	6	7	8	9	10	11	12	13	14	15	16	17	18	19	20
		21	22	23	24	25	26	27	28	29	30	31	32	33	34	35	36	37	38	39	40
	F	1	2	3	4	5	6	7	8	9	10	11	12	13	14	15	16	17	18	19	20
		21	22	23	24	25	26	27	28	29	30	31	32	33	34	35	36	37	38	39	40
ROTA(2)	M	1	2	3	4	5	6	7	8	9	10	11	12	13	14	15	16	17	18	19	20
		21	22	23	24	25	26	27	28	29	30	31	32	33	34	35	36	37	38	39	40
	F	1	2	3	4	5	6	7	8	9	10	11	12	13	14	15	16	17	18	19	20
		21	22	23	24	25	26	27	28	29	30	31	32	33	34	35	36	37	38	39	40
bOPV(3)	M	1	2	3	4	5	6	7	8	9	10	11	12	13	14	15	16	17	18	19	20
		21	22	23	24	25	26	27	28	29	30	31	32	33	34	35	36	37	38	39	40
	F	1	2	3	4	5	6	7	8	9	10	11	12	13	14	15	16	17	18	19	20
		21	22	23	24	25	26	27	28	29	30	31	32	33	34	35	36	37	38	39	40
PENTA(3)	M	1	2	3	4	5	6	7	8	9	10	11	12	13	14	15	16	17	18	19	20
		21	22	23	24	25	26	27	28	29	30	31	32	33	34	35	36	37	38	39	40
	F	1	2	3	4	5	6	7	8	9	10	11	12	13	14	15	16	17	18	19	20
		21	22	23	24	25	26	27	28	29	30	31	32	33	34	35	36	37	38	39	40
PCV13(3)	M	1	2	3	4	5	6	7	8	9	10	11	12	13	14	15	16	17	18	19	20
		21	22	23	24	25	26	27	28	29	30	31	32	33	34	35	36	37	38	39	40
	F	1	2	3	4	5	6	7	8	9	10	11	12	13	14	15	16	17	18	19	20
		21	22	23	24	25	26	27	28	29	30	31	32	33	34	35	36	37	38	39	40
ROTA(3)	M	1	2	3	4	5	6	7	8	9	10	11	12	13	14	15	16	17	18	19	20
		21	22	23	24	25	26	27	28	29	30	31	32	33	34	35	36	37	38	39	40
	F	1	2	3	4	5	6	7	8	9	10	11	12	13	14	15	16	17	18	19	20
		21	22	23	24	25	26	27	28	29	30	31	32	33	34	35	36	37	38	39	40
IPV(1)	M	1	2	3	4	5	6	7	8	9	10	11	12	13	14	15	16	17	18	19	20
		21	22	23	24	25	26	27	28	29	30	31	32	33	34	35	36	37	38	39	40
	F	1	2	3	4	5	6	7	8	9	10	11	12	13	14	15	16	17	18	19	20
		21	22	23	24	25	26	27	28	29	30	31	32	33	34	35	36	37	38	39	40
MCV(1)	M	1	2	3	4	5	6	7	8	9	10	11	12	13	14	15	16	17	18	19	20
		21	22	23	24	25	26	27	28	29	30	31	32	33	34	35	36	37	38	39	40
	F	1	2	3	4	5	6	7	8	9	10	11	12	13	14	15	16	17	18	19	20
		21	22	23	24	25	26	27	28	29	30	31	32	33	34	35	36	37	38	39	40
IPV(2)	M	1	2	3	4	5	6	7	8	9	10	11	12	13	14	15	16	17	18	19	20
		21	22	23	24	25	26	27	28	29	30	31	32	33	34	35	36	37	38	39	40
	F	1	2	3	4	5	6	7	8	9	10	11	12	13	14	15	16	17	18	19	20
		21	22	23	24	25	26	27	28	29	30	31	32	33	34	35	36	37	38	39	40
YF	M	1	2	3	4	5	6	7	8	9	10	11	12	13	14	15	16	17	18	19	20
		21	22	23	24	25	26	27	28	29	30	31	32	33	34	35	36	37	38	39	40
	F	1	2	3	4	5	6	7	8	9	10	11	12	13	14	15	16	17	18	19	20
		21	22	23	24	25	26	27	28	29	30	31	32	33	34	35	36	37	38	39	40

12-23 months: Mark an X on the number; M=Male, F=Female

bOPV(1)	M	1	2	3	4	5	6	7	8	9	10	11	12	13	14	15	16	17	18	19	20
		21	22	23	24	25	26	27	28	29	30	31	32	33	34	35	36	37	38	39	40
	F	1	2	3	4	5	6	7	8	9	10	11	12	13	14	15	16	17	18	19	20
		21	22	23	24	25	26	27	28	29	30	31	32	33	34	35	36	37	38	39	40
PENTA(1)	M	1	2	3	4	5	6	7	8	9	10	11	12	13	14	15	16	17	18	19	20
		21	22	23	24	25	26	27	28	29	30	31	32	33	34	35	36	37	38	39	40
	F	1	2	3	4	5	6	7	8	9	10	11	12	13	14	15	16	17	18	19	20
		21	22	23	24	25	26	27	28	29	30	31	32	33	34	35	36	37	38	39	40
PCV13(1)	M	1	2	3	4	5	6	7	8	9	10	11	12	13	14	15	16	17	18	19	20
		21	22	23	24	25	26	27	28	29	30	31	32	33	34	35	36	37	38	39	40
	F	1	2	3	4	5	6	7	8	9	10	11	12	13	14	15	16	17	18	19	20
		21	22	23	24	25	26	27	28	29	30	31	32	33	34	35	36	37	38	39	40
ROTA(1)	M	1	2	3	4	5	6	7	8	9	10	11	12	13	14	15	16	17	18	19	20
		21	22	23	24	25	26	27	28	29	30	31	32	33	34	35	36	37	38	39	40
	F	1	2	3	4	5	6	7	8	9	10	11	12	13	14	15	16	17	18	19	20
		21	22	23	24	25	26	27	28	29	30	31	32	33	34	35	36	37	38	39	40
bOPV(2)	M	1	2	3	4	5	6	7	8	9	10	11	12	13	14	15	16	17	18	19	20
		21	22	23	24	25	26	27	28	29	30	31	32	33	34	35	36	37	38	39	40
	F	1	2	3	4	5	6	7	8	9	10	11	12	13	14	15	16	17	18	19	20
		21	22	23	24	25	26	27	28	29	30	31	32	33	34	35	36	37	38	39	40
PENTA(2)	M	1	2	3	4	5	6	7	8	9	10	11	12	13	14	15	16	17	18	19	20
		21	22	23	24	25	26	27	28	29	30	31	32	33	34	35	36	37	38	39	40
	F	1	2	3	4	5	6	7	8	9	10	11	12	13	14	15	16	17	18	19	20
		21	22	23	24	25	26	27	28	29	30	31	32	33	34	35	36	37	38	39	40
PCV13(2)	M	1	2	3	4	5	6	7	8	9	10	11	12	13	14	15	16	17	18	19	20
		21	22	23	24	25	26	27	28	29	30	31	32	33	34	35	36	37	38	39	40
	F	1	2	3	4	5	6	7	8	9	10	11	12	13	14	15	16	17	18	19	20
		21	22	23	24	25	26	27	28	29	30	31	32	33	34	35	36	37	38	39	40
ROTA(2)	M	1	2	3	4	5	6	7	8	9	10	11	12	13	14	15	16	17	18	19	20
		21	22	23	24	25	26	27	28	29	30	31	32	33	34	35	36	37	38	39	40
	F	1	2	3	4	5	6	7	8	9	10	11	12	13	14	15	16	17	18	19	20
		21	22	23	24	25	26	27	28	29	30	31	32	33	34	35	36	37	38	39	40
bOPV(3)	M	1	2	3	4	5	6	7	8	9	10	11	12	13	14	15	16	17	18	19	20
		21	22	23	24	25	26	27	28	29	30	31	32	33	34	35	36	37	38	39	40
	F	1	2	3	4	5	6	7	8	9	10	11	12	13	14	15	16	17	18	19	20
		21	22	23	24	25	26	27	28	29	30	31	32	33	34	35	36	37	38	39	40
PENTA(3)	M	1	2	3	4	5	6	7	8	9	10	11	12	13	14	15	16	17	18	19	20
		21	22	23	24	25	26	27	28	29	30	31	32	33	34	35	36	37	38	39	40
	F	1	2	3	4	5	6	7	8	9	10	11	12	13	14	15	16	17	18	19	20
		21	22	23	24	25	26	27	28	29	30	31	32	33	34	35	36	37	38	39	40
ROTA(3)	M	1	2	3	4	5	6	7	8	9	10	11	12	13	14	15	16	17	18	19	20
		21	22	23	24	25	26	27	28	29	30	31	32	33	34	35	36	37	38	39	40
	F	1	2	3	4	5	6	7	8	9	10	11	12	13	14	15	16	17	18	19	20
		21	22	23	24	25	26	27	28	29	30	31	32	33	34	35	36	37	38	39	40
IPV(1)	M	1	2	3	4	5	6	7	8	9	10	11	12	13	14	15	16	17	18	19	20
		21	22	23	24	25	26	27	28	29	30	31	32	33	34	35	36	37	38	39	40
	F	1	2	3	4	5	6	7	8	9	10	11	12	13	14	15	16	17	18	19	20
		21	22	23	24	25	26	27	28	29	30	31	32	33	34	35	36	37	38	39	40
MCV(1)	M	1	2	3	4	5	6	7	8	9	10	11	12	13	14	15	16	17	18	19	20
		21	22	23	24	25	26	27	28	29	30	31	32	33	34	35	36	37	38	39	40
	F	1	2	3	4	5	6	7	8	9	10	11	12	13	14	15	16	17	18	19	20
		21	22	23	24	25	26	27	28	29	30	31	32	33	34	35	36	37	38	39	40
IPV(2)	M	1	2	3	4	5	6	7	8	9	10	11	12	13	14	15	16	17	18	19	20
		21	22	23	24	25	26	27	28	29	30	31	32	33	34	35	36	37	38	39	40
	F	1	2	3	4	5	6	7	8	9	10	11	12	13	14	15	16	17	18	19	20
		21	22	23	24	25	26	27	28	29	30	31	32	33	34	35	36	37	38	39	40
MCV(2)	M	1	2	3	4	5	6	7	8	9	10	11	12	13	14	15	16	17	18	19	20
		21	22	23	24	25	26	27	28	29	30	31	32	33	34	35	36	37	38	39	40
	F	1	2	3	4	5	6	7	8	9	10	11	12	13	14	15	16	17	18	19	20
		21	22	23	24	25	26	27	28	29	30	31	32	33	34	35	36	37	38	39	40
YF	M	1	2	3	4	5	6	7	8	9	10	11	12	13	14	15	16	17	18	19	20
		21	22	23	24	25	26	27	28	29	30	31	32	33	34	35	36	37	38	39	40
	F	1	2	3	4	5	6	7	8	9	10	11	12	13	14	15	16	17	18	19	20
		21	22	23	24	25	26	27	28	29	30	31	32	33	34	35	36	37	38	39	40

24-59 months: Mark an X on the number; M=Male, F=Female

bOPV(1)	M	1	2	3	4	5	6	7	8	9	10	11	12	13	14	15	16	17	18	19	20
		21	22	23	24	25	26	27	28	29	30	31	32	33	34	35	36	37	38	39	40
	F	1	2	3	4	5	6	7	8	9	10	11	12	13	14	15	16	17	18	19	20
		21	22	23	24	25	26	27	28	29	30	31	32	33	34	35	36	37	38	39	40
PENTA(1)	M	1	2	3	4	5	6	7	8	9	10	11	12	13	14	15	16	17	18	19	20
		21	22	23	24	25	26	27	28	29	30	31	32	33	34	35	36	37	38	39	40
	F	1	2	3	4	5	6	7	8	9	10	11	12	13	14	15	16	17	18	19	20
		21	22	23	24	25	26	27	28	29	30	31	32	33	34	35	36	37	38	39	40
PCV13(1)	M	1	2	3	4	5	6	7	8	9	10	11	12	13	14	15	16	17	18	19	20
		21	22	23	24	25	26	27	28	29	30	31	32	33	34	35	36	37	38	39	40
	F	1	2	3	4	5	6	7	8	9	10	11	12	13	14	15	16	17	18	19	20
		21	22	23	24	25	26	27	28	29	30	31	32	33	34	35	36	37	38	39	40
bOPV(2)	M	1	2	3	4	5	6	7	8	9	10	11	12	13	14	15	16	17	18	19	20
		21	22	23	24	25	26	27	28	29	30	31	32	33	34	35	36	37	38	39	40
	F	1	2	3	4	5	6	7	8	9	10	11	12	13	14	15	16	17	18	19	20
		21	22	23	24	25	26	27	28	29	30	31	32	33	34	35	36	37	38	39	40
PENTA(2)	M	1	2	3	4	5	6	7	8	9	10	11	12	13	14	15	16	17	18	19	20
		21	22	23	24	25	26	27	28	29	30	31	32	33	34	35	36	37	38	39	40
	F	1	2	3	4	5	6	7	8	9	10	11	12	13	14	15	16	17	18	19	20
		21	22	23	24	25	26	27	28	29	30	31	32	33	34	35	36	37	38	39	40
bOPV(3)	M	1	2	3	4	5	6	7	8	9	10	11	12	13	14	15	16	17	18	19	20
		21	22	23	24	25	26	27	28	29	30	31	32	33	34	35	36	37	38	39	40
	F	1	2	3	4	5	6	7	8	9	10	11	12	13	14	15	16	17	18	19	20
		21	22	23	24	25	26	27	28	29	30	31	32	33	34	35	36	37	38	39	40
PENTA(3)	M	1	2	3	4	5	6	7	8	9	10	11	12	13	14	15	16	17	18	19	20
		21	22	23	24	25	26	27	28	29	30	31	32	33	34	35	36	37	38	39	40
	F	1	2	3	4	5	6	7	8	9	10	11	12	13	14	15	16	17	18	19	20
		21	22	23	24	25	26	27	28	29	30	31	32	33	34	35	36	37	38	39	40
IPV(1)	M	1	2	3	4	5	6	7	8	9	10	11	12	13	14	15	16	17	18	19	20
		21	22	23	24	25	26	27	28	29	30	31	32	33	34	35	36	37	38	39	40
	F	1	2	3	4	5	6	7	8	9	10	11	12	13	14	15	16	17	18	19	20
		21	22	23	24	25	26	27	28	29	30	31	32	33	34	35	36	37	38	39	40
MCV(1)	M	1	2	3	4	5	6	7	8	9	10	11	12	13	14	15	16	17	18	19	20
		21	22	23	24	25	26	27	28	29	30	31	32	33	34	35	36	37	38	39	40
	F	1	2	3	4	5	6	7	8	9	10	11	12	13	14	15	16	17	18	19	20
		21	22	23	24	25	26	27	28	29	30	31	32	33	34	35	36	37	38	39	40
IPV(2)	M	1	2	3	4	5	6	7	8	9	10	11	12	13	14	15	16	17	18	19	20
		21	22	23	24	25	26	27	28	29	30	31	32	33	34	35	36	37	38	39	40
	F	1	2	3	4	5	6	7	8	9	10	11	12	13	14	15	16	17	18	19	20
		21	22	23	24	25	26	27	28	29	30	31	32	33	34	35	36	37	38	39	40
MCV(2)	M	1	2	3	4	5	6	7	8	9	10	11	12	13	14	15	16	17	18	19	20
		21	22	23	24	25	26	27	28	29	30	31	32	33	34	35	36	37	38	39	40
	F	1	2	3	4	5	6	7	8	9	10	11	12	13	14	15	16	17	18	19	20
		21	22	23	24	25	26	27	28	29	30	31	32	33	34	35	36	37	38	39	40
YF	M	1	2	3	4	5	6	7	8	9	10	11	12	13	14	15	16	17	18	19	20
		21	22	23	24	25	26	27	28	29	30	31	32	33	34	35	36	37	38	39	40
	F	1	2	3	4	5	6	7	8	9	10	11	12	13	14	15	16	17	18	19	20
		21	22	23	24	25	26	27	28	29	30	31	32	33	34	35	36	37	38	39	40
