# Supplementary material for: Reaching Never- and Incompletely-Vaccinated Children with Routine Immunization: A Proof-of-Concept Activity Using Geo-Referenced Microplans in Two Health Zones in Maniema Province, Democratic Republic of the Congo
Source: Vaccines (Basel). 2026 Feb 13;14(2):175. doi: 10.3390/vaccines14020175 (PMC12945245; doi:10.3390/vaccines14020175)
Supplement: Supplementary file 1 [file vaccines-14-00175-s001.zip › Tools_Alleman_Maniema_10Feb2026.zip!/K1_Tool_S11A_Post_Vaccinarion_PIRI_Coupon_French.pdf]

Activités de vaccination 2023/2024. Projet de renforcement de la vaccination de routine chez des enfants non-vaccinés ou insuffisamment vaccinés à l’aide de micro-plan validés dans les zones de sante à haut risque de transmission de la polio et de la rougeole, Kindu/Kibombo, Maniema, RDC

Jeton de rattrapage vaccinal 2023/2024, uniquement pour les enfants 24-59 mois

Emmenez votre enfant au poste de vaccination pour le protéger!

Faites un X à côté de la réponse pour chaque vaccin que l'enfant a reçu

Nom de l'enfant: ______________________________

Penta1	☐	Penta2	☐	Penta3	☐
PCV13(1)	☐				
VPOb(1)	☐	VPOb(2)	☐	VPOb(3)	☐
VPI(1)	☐	VPI(2)	☐		
VAR(1)	☐	VAR(2)	☐		
VAA	☐				
