# Supplementary material for: Reaching Never- and Incompletely-Vaccinated Children with Routine Immunization: A Proof-of-Concept Activity Using Geo-Referenced Microplans in Two Health Zones in Maniema Province, Democratic Republic of the Congo
Source: Vaccines (Basel). 2026 Feb 13;14(2):175. doi: 10.3390/vaccines14020175 (PMC12945245; doi:10.3390/vaccines14020175)
Supplement: Supplementary file 1 [file vaccines-14-00175-s001.zip › Tools_Alleman_Maniema_10Feb2026.zip!/K2_Tool_S11B_Post_Vaccination_PIRI_Coupon_English.pdf]

Vaccination Activities 2023/2024. Project to reinforce routine vaccination among children never vaccinated or incompletely vaccinated using validated microplans in health zones at high risk for transmission of measles and polio, Kindu and Kibombo, Maniema Province, DRC

Coupon for Catch-Up Vaccination, 2023/2024, only for children 24-59 months

Bring your child to the vaccination site to protect them!

Mark an X next to the vaccine that the child received

Name of the child: ____

Penta1	☐	Penta2	☐	Penta3	☐
PCV13(1)	☐				
bOPV(1)	☐	bOPV(2)	☐	bOPV(3)	☐
IPV(1)	☐	IPV(2)	☐		
MCV(1)	☐	MCV(2)	☐		
YF	☐				
