# Supplementary material for: Reaching Never- and Incompletely-Vaccinated Children with Routine Immunization: A Proof-of-Concept Activity Using Geo-Referenced Microplans in Two Health Zones in Maniema Province, Democratic Republic of the Congo
Source: Vaccines (Basel). 2026 Feb 13;14(2):175. doi: 10.3390/vaccines14020175 (PMC12945245; doi:10.3390/vaccines14020175)
Supplement: Supplementary file 1 [file vaccines-14-00175-s001.zip › Tools_Alleman_Maniema_10Feb2026.zip!/L1_Tool_S12A_Maniema_PIRI_Provenance_Tally_French.pdf]

Fiche de provenance:

Activités de vaccination, Projet de renforcement de la vaccination de routine chez des enfants non-vaccinés ou insuffisamment vaccinés à l'aide de micro plans validés dans les zones de santé à haut risque de transmission de la polio et de la rougeole, Kindu/Kibombo, Maniema, RDC

1. Zone de Santé :	2. Aire de Santé :	3. Noms des membres de l'équipe de vaccination (Jina ya wanamemba wenye ku chanja) :	4. Code Equipe :
5. Date de la séance (Tarehe ya siku ya chanjo) :		6. Nom du poste de vaccination (Nom de l'établissement de soin de santé ou de la localité du poste si hors d'une établissement de soin de santé) (Jina ya fasi yenye ku fanyika chanjo) :	
7. Le poste de vaccination, est-il une établissement de soin de santé privée? (Faites un X à côté de la réponse) :<br>_____ Oui ____Non		8. Type de stratégie de vaccination (Faites un X à côté de la stratégie) :<br>Fasi ya chanjo (Tiya musalaba nyuma ya fasi ya chanjo, kama ni ku baraza ya afya ao fasi ingine) : _______Fixe ______Avancée ______Mobile	9. Numéro de passage (Faites un X à côté du passage<br>_____1er _____2e _____3e

	Provenance d'enfant (Fasi mutoto anaishi) (localité d'habitation ou de visite, utiliser la liste des aires de santé et localités pour Kindu/Kibombo) (jina ya village ama avenue ama bloc ya Kindu/Kibombo)		Enfant avec jeton jaune IPVS (Mtoto iko na jeton)	Catégorie d'âge Fasi ya miaka ya mtoto			Enfant jamais vacciné à l'hôpital ou centre de santé ? (Mtoto anaishaka chanjiwa?)	Participé aux passage(s) précédent(s) (Mtoto alichanjwa mara ya kwanza ya pili) #1	#2
	Aire de Santé (de la liste, si autre que Kindu/Kibombo, écrivez "Autres")	Localité (de la liste, si autre que Kindu/Kibombo, écrivez "Autres")	Oui(Ndiyo)=1 Non(Apana)=0	0 - 11 mois	12 - 23 mois	24-59 mois	OUI (Ndiyo) =1 NON (Apana) =0	OUI (Ndiyo) =1 NON (Apana) =0	OUI (Ndiyo) =1 NON (Apana) =0
1									
2									
3									
4									
5									
6									
7									
8									
9									
10									
11									
12									
13									
14									
15									
16									
17									
18									
19									
20									
21									
22									
23									
24									
25									
26									
27									
28									
29									
30									
31									
32									
33									
34									
35									
36									
37									

	Provenance d'enfant (Fasi mutoto anaishi) (localité d'habitation ou de visite, utiliser la liste des aires de santé et localités pour Kindu/Kibombo) (jina ya village ama avenue ama bloc ya Kindu/Kibombo)		Enfant avec jeton IPVS (Mtoto iko na jeton)	Catégorie d'âge Fasi ya miaka ya mtoto			Enfant jamais vacciné à l'hôpital ou centre de santé ? (Mtoto anaishaka chanjiwa?)	Participé aux passag (Mtoto alichanjwa ya pi #1
	Aire de Santé (de la liste, si autre que Kindu/Kibombo, écrivez "Autres")	Localité (de la liste, si autre que Kindu/Kibombo, écrivez "Autres")	OUI (Ndiyo) =1 NON (Apana) =0	0 - 11 mois	12 - 23 mois	24-59 mois	OUI (Ndiyo) =1 NON (Apana) =0	OUI (Ndiyo) =1 NON (Apana) =0
38								
39								
40								
41								
42								
43								
44								
45								
46								
47								
48								
49								
50								
51								
52								
53								
54								
55								
56								
57								
58								
59								
60								
61								
62								
63								
64								
65								
66								
67								
68								
69								
70								
71								
72								
73								
74								

actuel) :
;e(s) précédent(s) mara ya kwanza ili) #2
OUI (Ndiyo) =1 NON (Apana) =0
