# Supplementary material for: Reaching Never- and Incompletely-Vaccinated Children with Routine Immunization: A Proof-of-Concept Activity Using Geo-Referenced Microplans in Two Health Zones in Maniema Province, Democratic Republic of the Congo
Source: Vaccines (Basel). 2026 Feb 13;14(2):175. doi: 10.3390/vaccines14020175 (PMC12945245; doi:10.3390/vaccines14020175)
Supplement: Supplementary file 1 [file vaccines-14-00175-s001.zip › Tools_Alleman_Maniema_10Feb2026.zip!/L2_Tool_S12B_Maniema_PIRI_Provenance_Tally_English.pdf]

Provenance Form:

Vaccination Activities 2023. Project to reinforce routine vaccination among children never vaccinated or incompletely vaccinated using validated microplans in health zones at high risk for transmission of measles and polio, Kindu and Kibombo, Maniema Province, Democratic Republic of the Congo

1. Health Zone:	2. Health Area:	3. Names of the members of the vaccination team:	4. Team Code:
5. Date of the session:		6. Name of the vaccination site (Name of the health facility or the locality of the session if held outside of a health facility:	
7. Is the vaccination site a private health facility (Mark an X next to the response):<br>_____ Yes ____No		8. Type of vaccination strategy (Mark an X next to the strategy):<br>_______Fixed ______Avanced ______Mobile	9. Which PIRI? (Mark an X next to the ongoing PIRI):<br>_____1st _____2nd _____3rd

	Residence (Provenance of the child (locality of habitation or current visitation, use the list of health areas and localities provided for Kindu/Kibombo)		Did the child arrive with a coupon?	Age category			Was the child previously vaccinated at a hospital or health center at any time?	Did the child participate in previous PIRIs? #1	#2		Residence (Provenance of the child (locality of habitation or current visitation, use the list of health areas and localities provided for Kindu/Kibombo)		Did the child arrive with a coupon?	Age category			Was the child previously vaccinated at a hospital or health center at any time?	Did the child participate in previous PIRIs? #1	#2
	Health Area (from the list, if other than in Kindu/Kibombo, write "Other")	Locality (from the list, if other than in Kindu/Kibombo, write "Other")	Yes=1 No=0	0 - 11 mon	12 - 23 mon	24-59 mon	Yes=1 No=0	Yes =1 No =0	Yes =1 No=0		Health Area (from the list, if other than in Kindu/Kibombo, write "Other")	Locality (from the list, if other than in Kindu/Kibombo, write "Other")	Yes=1 No=0	0 - 11 mon	12 - 23 mon	24-59 mon	Yes=1 No=0	Yes=1 No=0	Yes=1 No=0
1										38									
2										39									
3										40									
4										41									
5										42									
6										43									
7										44									
8										45									
9										46									
10										47									
11										48									
12										49									
13										50									
14										51									
15										52									
16										53									
17										54									
18										55									
19										56									
20										57									
21										58									
22										59									
23										60									
24										61									
25										62									
26										63									
27										64									
28										65									
29										66									
30										67									
31										68									
32										69									
33										70									
34										71									
35										72									
36										73									
37										74									
