# Supplementary material for: Reaching Never- and Incompletely-Vaccinated Children with Routine Immunization: A Proof-of-Concept Activity Using Geo-Referenced Microplans in Two Health Zones in Maniema Province, Democratic Republic of the Congo
Source: Vaccines (Basel). 2026 Feb 13;14(2):175. doi: 10.3390/vaccines14020175 (PMC12945245; doi:10.3390/vaccines14020175)
Supplement: Supplementary file 1 [file vaccines-14-00175-s001.zip › Tools_Alleman_Maniema_10Feb2026.zip!/M1_Tool_S13A_Maniema_PIRI_VaccinationSite_Organization_French.pdf]

# Circuit du site de vaccination et tâches de l' équipe (3 personnes)

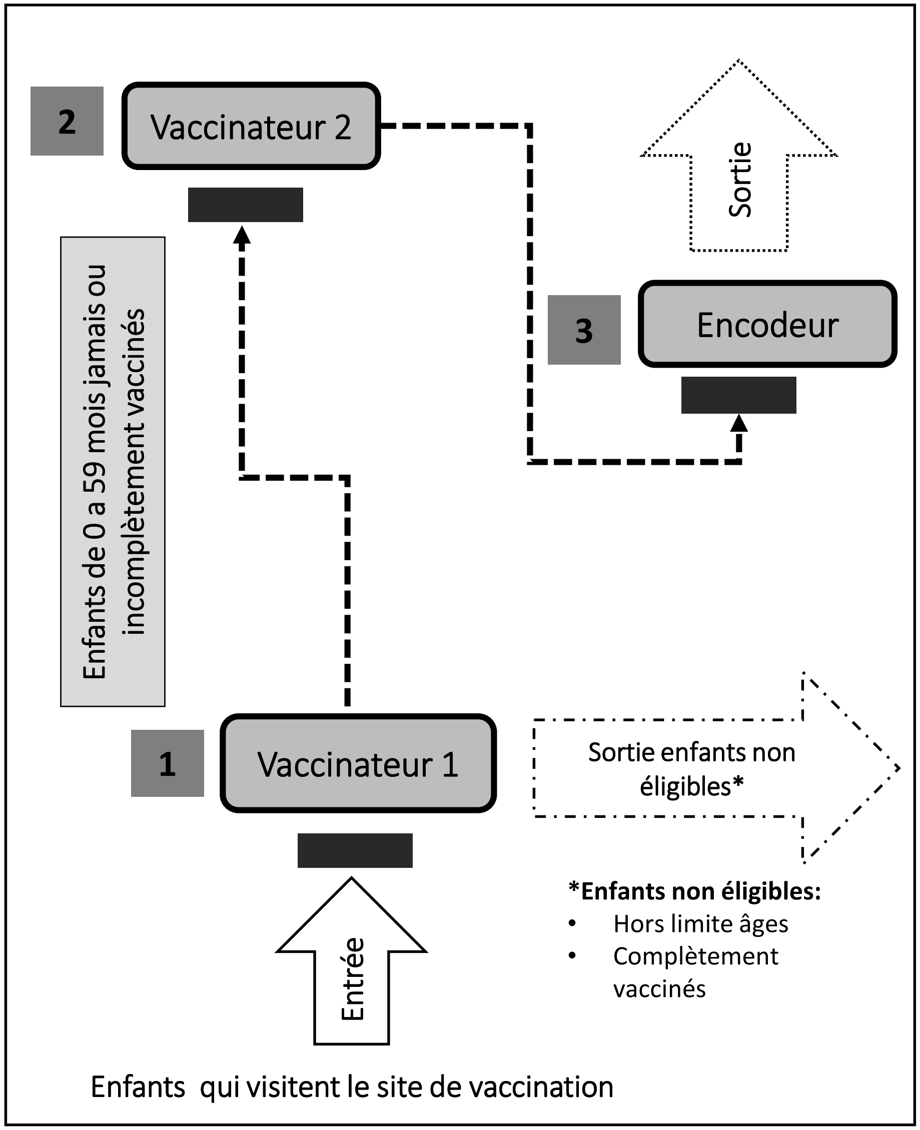


## Tâches de l'équipe:

### Vaccinateur 1:

- Vérifier le statut vaccinal des enfants
- Communiquer les vaccins a administrer au deuxième vaccinateur sur un bout de papier
- Remplir les généralités dans les fiches CPS et jeton bleu
- Remplir la fiche de provenance sur papier au fur et à mesure de l'arrivée des enfants
- Expliquer les effets secondaires et la conduite à tenir en cas de MAPI
- Inviter les enfants pour la prochaine IPVS
- Faire la gestion des déchets
- S'assurer que l'encodeur a saisi les données dans le téléphone

### Vaccinateur 2:

- Valider le statut vaccinal des enfants
- Préparer et administrer les vaccins
- Remplir les informations vaccinales dans les fiches CPS, le jeton bleu et dans la fiche de pointage papier.

### Encodeur:

*Pendant la séance:*

- Remplir la fiche de provenance sur téléphone pendant les temps morts (temps ou le circuit n'a pas d'enfants à vacciner)
- Inviter les enfants pour la prochaine IPVS
- Remplir les informations vaccinales dans le cahier de vaccination

*A la fin de la séance:*

- Terminer la saisie de la fiche de provenance dans le téléphone
- Saisir la fiche de pointage dans le téléphone

### Toute l' équipe:

- Remplir la fiche synthèse des fiches de pointage et de provenance à la fin de la journée
