# Supplementary material for: Reaching Never- and Incompletely-Vaccinated Children with Routine Immunization: A Proof-of-Concept Activity Using Geo-Referenced Microplans in Two Health Zones in Maniema Province, Democratic Republic of the Congo
Source: Vaccines (Basel). 2026 Feb 13;14(2):175. doi: 10.3390/vaccines14020175 (PMC12945245; doi:10.3390/vaccines14020175)
Supplement: Supplementary file 1 [file vaccines-14-00175-s001.zip › Tools_Alleman_Maniema_10Feb2026.zip!/M2_Tool_S13B_Maniema_PIRI_VaccinationSite_Organization_English.pdf]

# Set up for vaccination site and tasks of team members (3 persons)

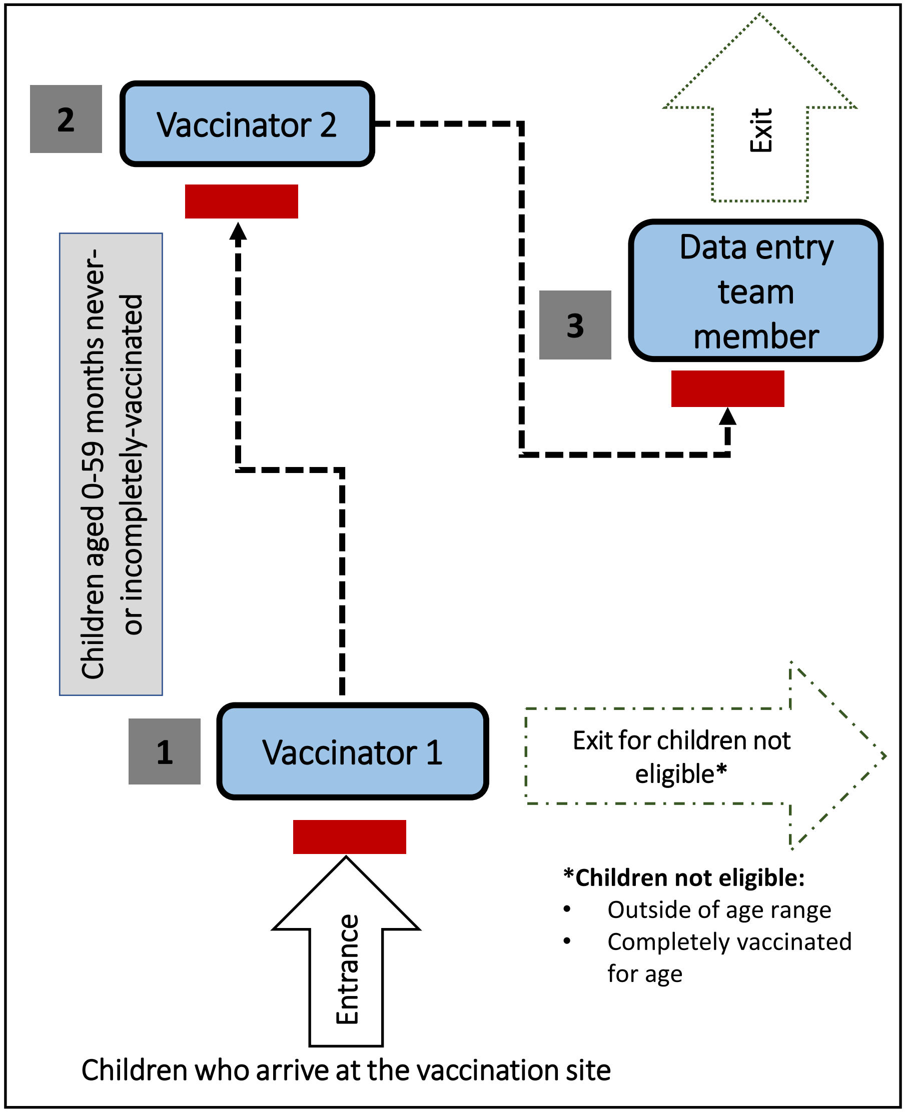


## Team member tasks:

### Vaccinator 1:

- Verify the vaccination status of each child
- Communicate the vaccines needed to the second vaccinator on a piece of paper
- Fill in the general information for each child of the vaccination card or the blue coupon
- Complete the paper copy "provenance form" for each child as they arrive
- Explain possible adverse events and actions to take if any occur
- Inform about the next PIRI
- Manage trash per immunization program protocol
- Ensure that the team member responsible for data entry entered all data into the telephone

### Vaccinator 2:

- Verify the vaccination status of each child
- Prepare and administer the vaccines
- Record vaccinations administered on the vaccination card or the blue coupon and the vaccination tally sheet

### Team member responsible for data entry into the telephone:

*During the session:*

- Enter data from the "provenance form" into the telephone during times when there are no children waiting for vaccination at the site
- Inform about the next PIRI
- Enter the data on vaccination into the vaccination register

*At the end of the session:*

- Complete entry of data from the "provenance form" into the telephone
- Enter data from the vaccination tally sheet into the telephone

### All members:

- Complete the paper forms that summarize the vaccination tally and provenance forms at the end of each day or session
