# Supplementary material for: Reaching Never- and Incompletely-Vaccinated Children with Routine Immunization: A Proof-of-Concept Activity Using Geo-Referenced Microplans in Two Health Zones in Maniema Province, Democratic Republic of the Congo
Source: Vaccines (Basel). 2026 Feb 13;14(2):175. doi: 10.3390/vaccines14020175 (PMC12945245; doi:10.3390/vaccines14020175)
Supplement: Supplementary file 1 [file vaccines-14-00175-s001.zip › Tools_Alleman_Maniema_10Feb2026.zip!/N1_Tool_S14A_Maniema_HealthArea_Daily_PIRI_Summary_French.pdf]

Fiche de synthèse Aire de Santé activités de vaccination 2023/2024<br>Projet de renforcement de la vaccination de routine chez des enfants non-vaccinés ou insuffisamment vaccinés à l'aide de micro-plan validés dans les zones de sante à haut risque de transmission de la polio et de la rougeole, Kindu/Kibombo, Maniema, RDC		
**Province :**	**Zone de Santé :**	**Aire de Santé :**
**Date des séances de vaccination :**	**Passage 1, 2 ou 3 ? (Faites un X à côté du passage actuel) :** ____ 1er ____ 2e_____ 3e	
Faites le total de la journée	***Féminin= F; Masculin = M; Total= T***	

0-11 mois

BCG	M	
	F	
	T	
VPOb(0)	M	
	F	
	T	
VPOb(1)	M	
	F	
	T	
PENTA(1)	M	
	F	
	T	
PCV13(1)	M	
	F	
	T	
ROTA(1)	M	
	F	
	T	
VPOb(2)	M	
	F	
	T	
PENTA(2)	M	
	F	
	T	
PCV13(2)	M	
	F	
	T	
ROTA(2)	M	
	F	
	T	
VPOb(3)	M	
	F	
	T	
PENTA(3)	M	
	F	
	T	
PCV13(3)	M	
	F	
	T	
ROTA(3)	M	
	F	
	T	
VPI(1)	M	
	F	
	T	
VAR(1)	M	
	F	
	T	
VPI(2)	M	
	F	
	T	
VAA	M	
	F	
	T	

12-23 mois

VPOb(1)	M	
	F	
	T	
PENTA(1)	M	
	F	
	T	
PCV13(1)	M	
	F	
	T	
ROTA(1)	M	
	F	
	T	
VPOb(2)	M	
	F	
	T	
PENTA(2)	M	
	F	
	T	
PCV13(2)	M	
	F	
	T	
ROTA(2)	M	
	F	
	T	
VPOb(3)	M	
	F	
	T	
PENTA(3)	M	
	F	
	T	
ROTA(3)	M	
	F	
	T	
VPI(1)	M	
	F	
	T	
VAR(1)	M	
	F	
	T	
VPI(2)	M	
	F	
	T	
VAR(2)	M	
	F	
	T	
VAA	M	
	F	
	T	

24-59 mois

VPOb(1)	M	
	F	
	T	
PENTA(1)	M	
	F	
	T	
PCV13(1)	M	
	F	
	T	
VPOb(2)	M	
	F	
	T	
PENTA(2)	M	
	F	
	T	
VPOb(3)	M	
	F	
	T	
PENTA(3)	M	
	F	
	T	
VPI(1)	M	
	F	
	T	
VAR(1)	M	
	F	
	T	
VPI(2)	M	
	F	
	T	
VAR(2)	M	
	F	
	T	
VAA	M	
	F	
	T	

Total

BCG	M	
	F	
	T	
VPOb(0)	M	
	F	
	T	
VPOb(1)	M	
	F	
	T	
PENTA(1)	M	
	F	
	T	
PCV13(1)	M	
	F	
	T	
ROTA(1)	M	
	F	
	T	
VPOb(2)	M	
	F	
	T	
PENTA(2)	M	
	F	
	T	
PCV13(2)	M	
	F	
	T	
ROTA(2)	M	
	F	
	T	
VPOb(3)	M	
	F	
	T	
PENTA(3)	M	
	F	
	T	
PCV13(3)	M	
	F	
	T	
ROTA(3)	M	
	F	
	T	
VPI(1)	M	
	F	
	T	
VAR(1)	M	
	F	
	T	
VPI(2)	M	
	F	
	T	
VAR(2)	M	
	F	
	T	
VAA	M	
	F	
	T	

SYNTHESE NOMBRE DES ENFANTS DANS LA FICHE DE PROVENANCE

0-11 Mois		12-23 Mois		24-59 Mois		TOTAL	
