# Supplementary material for: Reaching Never- and Incompletely-Vaccinated Children with Routine Immunization: A Proof-of-Concept Activity Using Geo-Referenced Microplans in Two Health Zones in Maniema Province, Democratic Republic of the Congo
Source: Vaccines (Basel). 2026 Feb 13;14(2):175. doi: 10.3390/vaccines14020175 (PMC12945245; doi:10.3390/vaccines14020175)
Supplement: Supplementary file 1 [file vaccines-14-00175-s001.zip › Tools_Alleman_Maniema_10Feb2026.zip!/N2_Tool_S14B_Maniema_HealthArea_Daily_PIRI_Summary_English.pdf]

Summary Form Health Area vaccination activities 2023/2024

Project to reinforce routine vaccination among children never vaccinated or incompletely vaccinated using validated microplans in health zones at high risk for transmission of measles and polio, Kindu and Kibombo, Maniema Province, DRC

**Province :**	**Health Zone :**	**Health Area :**
**Date of vaccination session:**	**PIRI 1, 2, or 3 ? (Mark an "X" for the relevant PIRI) : ____ 1st ____ 2nd_____ 3rd**	
Enter the total for the day	***Female= F; Male = M; Total= T***	

**0-11 months**			**12-23 months**			**24-59 months**			**Total**		
**BCG**	**M**								**BCG**	**M**	
	**F**									**F**	
	**T**									**T**	
**bOPV(0)**	**M**								**bOPV(0)**	**M**	
	**F**									**F**	
	**T**									**T**	
**bOPV(1)**	**M**		**bOPV(1)**	**M**		**bOPV(1)**	**M**		**bOPV(1)**	**M**	
	**F**			**F**			**F**			**F**	
	**T**			**T**			**T**			**T**	
**PENTA(1)**	**M**		**PENTA(1)**	**M**		**PENTA(1)**	**M**		**PENTA(1)**	**M**	
	**F**			**F**			**F**			**F**	
	**T**			**T**			**T**			**T**	
**PCV13(1)**	**M**		**PCV13(1)**	**M**		**PCV13(1)**	**M**		**PCV13(1)**	**M**	
	**F**			**F**			**F**			**F**	
	**T**			**T**			**T**			**T**	
**ROTA(1)**	**M**		**ROTA(1)**	**M**					**ROTA(1)**	**M**	
	**F**			**F**						**F**	
	**T**			**T**						**T**	
**bOPV(2)**	**M**		**bOPV(2)**	**M**		**bOPV(2)**	**M**		**bOPV(2)**	**M**	
	**F**			**F**			**F**			**F**	
	**T**			**T**			**T**			**T**	
**PENTA(2)**	**M**		**PENTA(2)**	**M**		**PENTA(2)**	**M**		**PENTA(2)**	**M**	
	**F**			**F**			**F**			**F**	
	**T**			**T**			**T**			**T**	
**PCV13(2)**	**M**		**PCV13(2)**	**M**					**PCV13(2)**	**M**	
	**F**			**F**						**F**	
	**T**			**T**						**T**	
**ROTA(2)**	**M**		**ROTA(2)**	**M**					**ROTA(2)**	**M**	
	**F**			**F**						**F**	
	**T**			**T**						**T**	
**bOPV(3)**	**M**		**bOPV(3)**	**M**		**bOPV(3)**	**M**		**bOPV(3)**	**M**	
	**F**			**F**			**F**			**F**	
	**T**			**T**			**T**			**T**	
**PENTA(3)**	**M**		**PENTA(3)**	**M**		**PENTA(3)**	**M**		**PENTA(3)**	**M**	
	**F**			**F**			**F**			**F**	
	**T**			**T**			**T**			**T**	
**PCV13(3)**	**M**								**PCV13(3)**	**M**	
	**F**									**F**	
	**T**									**T**	
**ROTA(3)**	**M**		**ROTA(3)**	**M**					**ROTA(3)**	**M**	
	**F**			**F**						**F**	
	**T**			**T**						**T**	
**IPV(1)**	**M**		**IPV(1)**	**M**		**IPV(1)**	**M**		**IPV(1)**	**M**	
	**F**			**F**			**F**			**F**	
	**T**			**T**			**T**			**T**	
**MCV(1)**	**M**		**MCV(1)**	**M**		**MCV(1)**	**M**		**MCV(1)**	**M**	
	**F**			**F**			**F**			**F**	
	**T**			**T**			**T**			**T**	
**IPV(2)**	**M**		**IPV(2)**	**M**		**IPV(2)**	**M**		**IPV(2)**	**M**	
	**F**			**F**			**F**			**F**	
	**T**			**T**			**T**			**T**	
			**MCV(2)**	**M**		**MCV(2)**	**M**		**MCV(2)**	**M**	
				**F**			**F**			**F**	
				**T**			**T**			**T**	
**YF**	**M**		**YF**	**M**		**YF**	**M**		**YF**	**M**	
	**F**			**F**			**F**			**F**	
	**T**			**T**			**T**			**T**	

SUMMARY: NUMBER OF CHILDREN ON PROVENANCE FORM

**0-11 Mois**		**12-23 Mois**		**24-59 Mois**		**TOTAL**	
