# Supplementary material for: Reaching Never- and Incompletely-Vaccinated Children with Routine Immunization: A Proof-of-Concept Activity Using Geo-Referenced Microplans in Two Health Zones in Maniema Province, Democratic Republic of the Congo
Source: Vaccines (Basel). 2026 Feb 13;14(2):175. doi: 10.3390/vaccines14020175 (PMC12945245; doi:10.3390/vaccines14020175)
Supplement: Supplementary file 1 [file vaccines-14-00175-s001.zip › Tools_Alleman_Maniema_10Feb2026.zip!/O1_Tool_S15A_Maniema_HealthZone_Daily_PIRI_Summary_French.pdf]

Fiche de synthèse Zone de Santé activités de vaccination 2023/2024
Projet de renforcement de la vaccination de routine chez des enfants non-vaccinés ou insuffisamment vaccinés à l'aide de micro-plan validés dans les zones de sante à haut risque de transmission de la polio et de la rougeole, Kindu/Kibombo, Maniema, RDC

**Province :**	**Zone de Santé :**
**Date des séances de vaccination :**	**Passage 1, 2 ou 3 ? (Faites un X à côté du passage actuel) : ____ 1er ____ 2e_____ 3e**
Faites le total de la journée	***Féminin= F; Masculin = M; Total= T***

0-11 mois			12-23 mois			24-59 mois			Total		
BCG	M								BCG	M	
	F									F	
	T									T	
VPOb(0)	M								VPOb(0)	M	
	F									F	
	T									T	
VPOb(1)	M		VPOb(1)	M		VPOb(1)	M		VPOb(1)	M	
	F			F			F			F	
	T			T			T			T	
PENTA(1)	M		PENTA(1)	M		PENTA(1)	M		PENTA(1)	M	
	F			F			F			F	
	T			T			T			T	
PCV13(1)	M		PCV13(1)	M		PCV13(1)	M		PCV13(1)	M	
	F			F			F			F	
	T			T			T			T	
ROTA(1)	M		ROTA(1)	M					ROTA(1)	M	
	F			F						F	
	T			T						T	
VPOb(2)	M		VPOb(2)	M		VPOb(2)	M		VPOb(2)	M	
	F			F			F			F	
	T			T			T			T	
PENTA(2)	M		PENTA(2)	M		PENTA(2)	M		PENTA(2)	M	
	F			F			F			F	
	T			T			T			T	
PCV13(2)	M		PCV13(2)	M					PCV13(2)	M	
	F			F						F	
	T			T						T	
ROTA(2)	M		ROTA(2)	M					ROTA(2)	M	
	F			F						F	
	T			T						T	
VPOb(3)	M		VPOb(3)	M		VPOb(3)	M		VPOb(3)	M	
	F			F			F			F	
	T			T			T			T	
PENTA(3)	M		PENTA(3)	M		PENTA(3)	M		PENTA(3)	M	
	F			F			F			F	
	T			T			T			T	
PCV13(3)	M								PCV13(3)	M	
	F									F	
	T									T	
ROTA(3)	M		ROTA(3)	M					ROTA(3)	M	
	F			F						F	
	T			T						T	
VPI(1)	M		VPI(1)	M		VPI(1)	M		VPI(1)	M	
	F			F			F			F	
	T			T			T			T	
VAR(1)	M		VAR(1)	M		VAR(1)	M		VAR(1)	M	
	F			F			F			F	
	T			T			T			T	
VPI(2)	M		VPI(2)	M		VPI(2)	M		VPI(2)	M	
	F			F			F			F	
	T			T			T			T	
			VAR(2)	M		VAR(2)	M		VAR(2)	M	
				F			F			F	
				T			T			T	
VAA	M		VAA	M		VAA	M		VAA	M	
	F			F			F			F	
	T			T			T			T	

SYNTHESE NOMBRE DES ENFANTS DANS LA FICHE DE PROVENANCE

0-11 Mois		12-23 Mois		24-59 Mois		TOTAL	
