# Supplementary material for: Reaching Never- and Incompletely-Vaccinated Children with Routine Immunization: A Proof-of-Concept Activity Using Geo-Referenced Microplans in Two Health Zones in Maniema Province, Democratic Republic of the Congo
Source: Vaccines (Basel). 2026 Feb 13;14(2):175. doi: 10.3390/vaccines14020175 (PMC12945245; doi:10.3390/vaccines14020175)
Supplement: Supplementary file 1 [file vaccines-14-00175-s001.zip › Tools_Alleman_Maniema_10Feb2026.zip!/P1_Tool_S16A_Maniema_PIRI_Supervision_Checklist_French.pdf]

**Fiche de monitorage**<br>Activités de vaccination 2023/2024. Projet de renforcement de la vaccination de routine chez des enfants non-vaccinés ou insuffisamment vaccinés à l'aide de micro-plan validés dans les zones de sante à haut risque de transmission de la polio et de la rougeole, Kindu/Kibombo, Maniema, RDC	
**Outils**	
**10-Est-ce que les outils prévus pour la séance sont présents sur le site ?**	
Guide âge	Si non, donnez les outils manquants a l'équipe si vous les avez sur vous ou contacter le IT ou l'équipe cadre du bureau central de la zone de sante pour que l'envoie les outils manquants a l'équipe.<br><br>**Note** : La fiche synthèse des fiches de provenance et de pointage n'est pas incluse dans l'outil ODK. Prière de vérifier qu'elle est présente dans les sites de vaccination.
Calendrier vaccinal	
Les jetons jaunes	
Les jetons bleus	
Cartes CPS	
Antigènes selon l'âge	
Fiches de pointage	
Fiches de provenance	
Gestion des vaccins/MAPI	
Organisation du site	
Liste des localités	
Liste des équipes avec leur codes	
Fiche synthèse des fiches de provenance et de pointage	
**Organisation du site**	
11-Est-ce que le(s) vaccinateur(s) effectuent les tâches prévues ?	Si non, faite un révision des tâches à accomplir.
12-Est-ce que le pointeur effectue les tâches prévues ?	
13-Est-ce que tous les membres de l'équipe se trouvent sur place ?	Si non, appeler le/les membres manquants ou signaler a l'IT.
14-Est-ce que tous les 8 antigènes (BCG, VPOB, Penta, PCV13, ROTA, VPI, VAR, VAA) sont disponibles sur place ?	Si non, appeler l'IT pour approvisionnement ou aller chercher le matériel manquant pour le ramener a l'équipe.<br>Note : Les accumulateurs doivent être frais au toucher.
15-Si non quels antigènes sont manquants ?	
16-Est-ce qu'il y'a des vaccins au stade non utilisable sur le site ?	
17-Est-ce que tous les intrants (seringues, diluants et boites de sécurité) sont disponibles sur place ?	
18-Si non quels intrants sont manquants ?	
19-Est-ce que les accumulateurs sont disponibles sur place ?	
20-Est-ce que les accumulateurs sont frais?	
**Affluence**	
21-Est-ce que des enfants sont présents sur le site ?	Si non, discuter avec l'équipe et examiner la fiche de provenance pour évaluer l'affluence avant votre

	arrivée. Si aucun enfant n'est arrivé ou l'affluence est faible, contacter l'IT pour examiner la pertinence de revoir les stratégies de mobilisation ou la possibilité de déplacer le site de vaccination.
22-Si oui combien d'enfants sont en attente ?	
23-Combien d'enfants étaient déjà inscrits sur la fiche de provenance a votre arrivée ?	
**Qualité de la vaccination**	
24-Est-ce que vous avez pu observer l'administration de vaccins a au moins 1 enfant ?	
25-Est-ce que l'enfant est venu avec un jeton jaune ?	Si non, vérifier si le jeton a été oublie ou si le ménage n'a pas été visité par les RECOS
26-Est-ce que cet enfant a été correctement entré dans la fiche de provenance ?	Si non, aidez le pointeur a corriger ou a remplir correctement la fiche de provenance en réexpliquant le processus de remplissage si nécessaire.
27-Est-ce que l'infirmière a évalué le statut vaccinal de l'enfant selon l'âge de l'enfant ?	Si non, faites une pause dans le processus pour vous assurer que l'infirmière fait l'évaluation du statut vaccinal et au besoin utilise les outils appropries (guide d'âge, calendrier vaccinal, antigènes selon l'âge)
28-Si l'enfant a besoin d'être vacciné, est ce que le(s) vaccin(s) est/sont correctement administre(s) ?	Si non, faites une pause dans le processus pour aider l'infirmière a poser les actes corrects. Le calendrier vaccinal peut servir de guide pour la révision.
29-Est que les doses administrées sont correctement documentées (fiches de pointage, cahier de vaccination, cartes CPS / jetons bleus) ?	Si non, aidez l'infirmière à corriger ou à remplir correctement les documents en réexpliquant le processus de remplissage si nécessaire.
30-Est-ce que les vaccinateurs utilisent correctement la boîte de sécurité ?	Si non, rappeler l'importance d'utiliser la boite de sécurité. Si il n'y a pas de boite de sécurité, aller en chercher une ou appeler l'IT pour qu'il en amène une à l'équipe.
31-Est-ce que les vaccinateurs informent au sujet des MAPI ?	Si non, rappeler a l'infirmière de donner la bonne information aux enfants et a leurs accompagnateurs.
32-Est-ce que les vaccinateurs invitent les enfants pour la prochaine IPVS ?	
**Utilisation des téléphones**	
33-Est-ce que l'équipe a un téléphone qui marche sur place ?	Si non, contacter GRID3
34-Est-ce que les données de provenance sont rentrées progressivement dans le téléphone ?	Si non, rappeler au pointeur que les données de la fiche de provenance doivent être entrées au fur et a mesure, contrairement a la fiche de pointage qui va être entrée a la fin de la séance.
35-Est-ce que le téléphone est utilisé uniquement pour saisir les données de l'IPVS	Si non, rappeler au pointeur que ces utilisations personnelles sont interdites et vont consommer

(pas de carte SIM, pas d'applications téléchargées, pas de photos)	l'énergie des téléphones au détriment de la collecte de données.
