# Supplementary material for: Reaching Never- and Incompletely-Vaccinated Children with Routine Immunization: A Proof-of-Concept Activity Using Geo-Referenced Microplans in Two Health Zones in Maniema Province, Democratic Republic of the Congo
Source: Vaccines (Basel). 2026 Feb 13;14(2):175. doi: 10.3390/vaccines14020175 (PMC12945245; doi:10.3390/vaccines14020175)
Supplement: Supplementary file 1 [file vaccines-14-00175-s001.zip › Tools_Alleman_Maniema_10Feb2026.zip!/P2_Tool_S16B_Maniema_PIRI_Supervision_Checklist_English.pdf]

<table>
<tr><td colspan="2">Supervision Checklist
Vaccination Activities 2023/2024. Project to reinforce routine vaccination among children never vaccinated or incompletely vaccinated using validated microplans in health zones at high risk for transmission of measles and polio, Kindu and Kibombo, Maniema Province, Democratic Republic of the Congo</td></tr>
<tr><td colspan="2">Tools</td></tr>
<tr><td colspan="2">10-Are the tools prepared for use during the vaccination session available at the site?</td></tr>
<tr><td>Age Guide</td><td rowspan="12">If no, provide the missing tools to the team if you have them or contact the head nurse or the health zone office to provide them.

Note: The paper-based summary forms for the vaccination tally sheets and provenance forms are not part of the ODK tool on the telephone. Verify that these paper forms are available with the team.</td></tr>
<tr><td>Vaccination schedule</td></tr>
<tr><td>Yellow coupons</td></tr>
<tr><td>Blue coupons</td></tr>
<tr><td>Vaccination cards</td></tr>
<tr><td>Guides for vaccines by age</td></tr>
<tr><td>Vaccination tally sheets</td></tr>
<tr><td>Provenance forms</td></tr>
<tr><td>Vaccine management and adverse event forms</td></tr>
<tr><td>Set-up for vaccination site</td></tr>
<tr><td>Locality list</td></tr>
<tr><td>List of team code numbers</td></tr>
<tr><td>Paper-based summary forms for the vaccination tally sheets and provenance forms</td><td></td></tr>
<tr><td colspan="2"></td></tr>
<tr><td colspan="2">Set-up of the vaccination site</td></tr>
<tr><td>11-Are the vaccinators completing the tasks assigned?</td><td rowspan="2">If no, review tasks with the team members.</td></tr>
<tr><td>12- Is the person assigned to data entry completing the tasks assigned?</td></tr>
<tr><td>13-Are all team members present at the vaccination site?</td><td>If no, find/telephone any absent team members or signal their absence to the head nurse.</td></tr>
<tr><td>14-Are all vaccines (8) to be administered available at the site (BCG, bOPV, Penta, PCV13, ROTA, IPV, measles, yellow fever)?</td><td rowspan="7">If no, call the head nurse for a supply or gather the missing materials and bring them to the team.

Note: Ice packs in the vaccine carriers should be cool to the touch.</td></tr>
<tr><td>15-If no, which vaccines are missing?</td></tr>
<tr><td>16-Do the vaccine vial monitors on the vaccines at the site indicate a useable?</td></tr>
<tr><td>17-Are other necessary supplies available at the site (syringes, diluents and sharps disposal boxes)?</td></tr>
<tr><td>18-If no, which supplies are missing?</td></tr>
<tr><td>19-Are ice packs available at the site?</td></tr>
<tr><td>20-Are the ice packs cool?</td></tr>
<tr><td colspan="2">Situation at the site</td></tr>
<tr><td>21-Are children present at the site?</td><td>If no, review the provenance form to evaluate the number of children who had come to the site before your arrival. If no child or few children had come to the site, contact the</td></tr>
</table>

	head nurse to discuss. Should social mobilization strategies be revised? Should the team be moved to another site?
22-If yes, how many children are waiting?	
23-How many children had been registered on the provenance form at the time of your arrival?	
**Quality of vaccination administration**	
24-Did you observe the administration of vaccines to at least one child?	
25-Had the infant arrived to the site with a yellow coupon?	If no, verify if the family forgot to bring the coupon or if the household was not visited by a community volunteer.
26-Was the child's information correctly entered into the provenance form?	If no, assist the team member responsible for data entry with correcting erroneous entries, and if necessary, review the process for completing the form.
27-Is the vaccinator correctly evaluating the child's vaccination status according to their age?	If no, ask the vaccinator to pause activities. Ensure that the child is being correctly evaluated. If necessary, remind the nurse to use the appropriate tools for the evaluation (age guide, vaccination schedule, guide for vaccines by age)
28-If the child needs to be vaccinated, are the vaccines being administered correctly?	If no, ask the vaccinator to pause activities and review the correct procedures. Use available tools to discuss, if helpful.
29-Are the doses administered being correctly documented? (tally sheets, vaccination register, vaccination cards/blue coupons)?	If no, assist the vaccinator with correcting erroneous entries and if necessary, review the process for completing the forms.
30-Are the vaccinators using the sharps disposal box per protocol?	If no, remind them of the importance of the correct disposal of sharps. If the team does not have a sharps disposal box, find one or call the head nurse and request that one be delivered immediately.
31-Do the vaccinators explain the possibility of adverse events to caretakers?	If not, remind the vaccinator to discuss these subjects with caretakers.
32-Did the team inform the family of the next PIRI?	
**Use of the telephones**	
33-Does the team have a project phone that is functional?	If no, contact GRID3
34-Are the data describing the children's provenance being gradually entered into the telephone?	If no, remind the team member responsible for entry of data into the telephone that the provenance form data should be entered gradually, in contrast to the data from the vaccination tally sheet that is entered at the end of the session.
35-Is the project telephone being used uniquely to enter the PIRI data? (i.e., no SIM card inserted, no applications downloaded, no photos taken and stored)	If no, remind the team member responsible for entry of data into the telephone that personal use of the phone is prohibited and consumes battery life to the detriment of intended data collection.
