# Supplementary material for: Reaching Never- and Incompletely-Vaccinated Children with Routine Immunization: A Proof-of-Concept Activity Using Geo-Referenced Microplans in Two Health Zones in Maniema Province, Democratic Republic of the Congo
Source: Vaccines (Basel). 2026 Feb 13;14(2):175. doi: 10.3390/vaccines14020175 (PMC12945245; doi:10.3390/vaccines14020175)
Supplement: Supplementary file 1 [file vaccines-14-00175-s001.zip › Tools_Alleman_Maniema_10Feb2026.zip!/Q4_Tool_S20_Maniema_Kasuku1_Kindu_Combination_Map.pdf]

Enfants 0-23 Mois (Juillet - Octobre 2022)

Kasuku 1 Air de Santé

Kindu Zone de Santé

Pourcentage d’enfants jamais vaccinés à l'hôpital/centre de santé

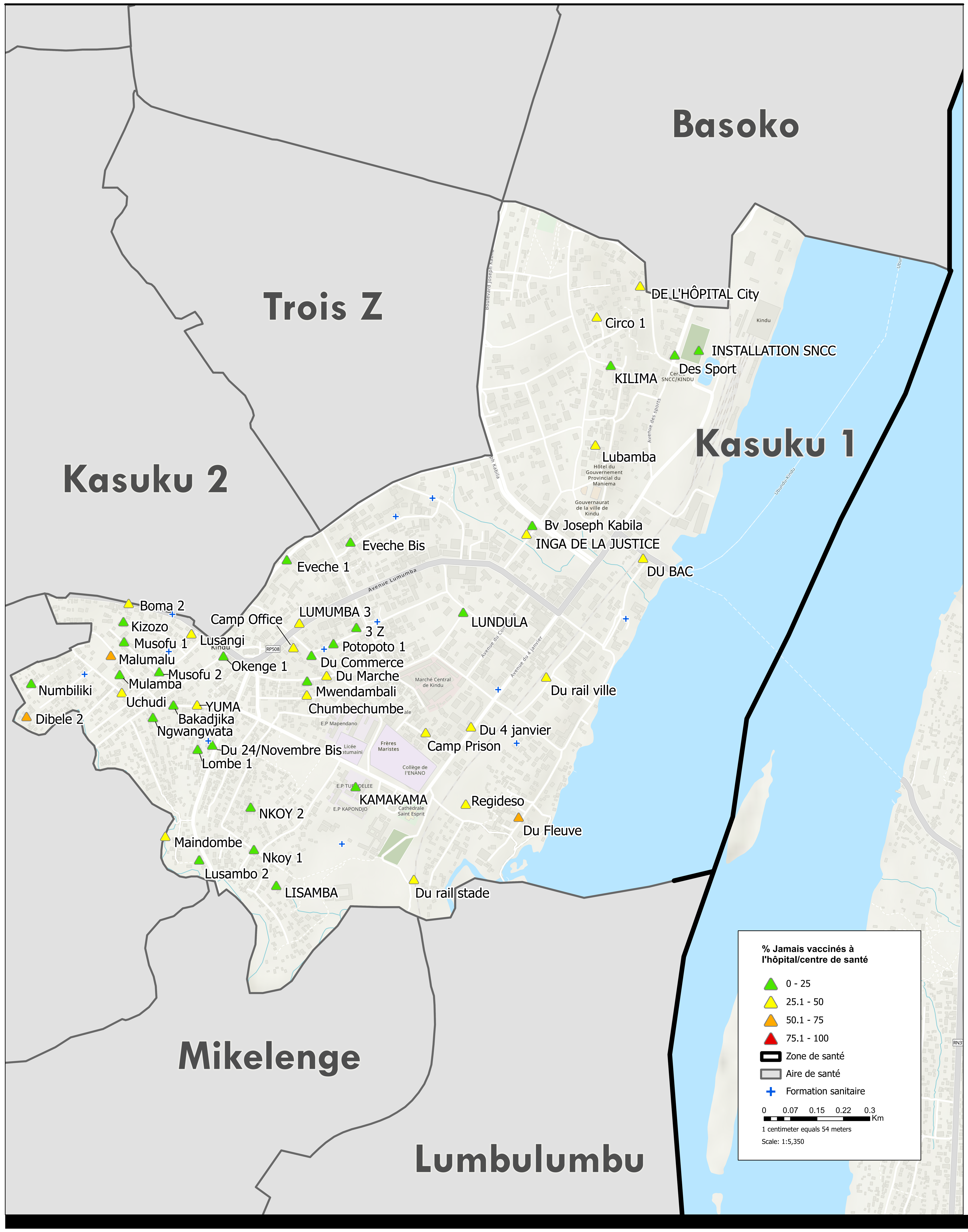

Raisons principales des enfants n'ont jamais été vaccinés à l'hôpital/centre de santé

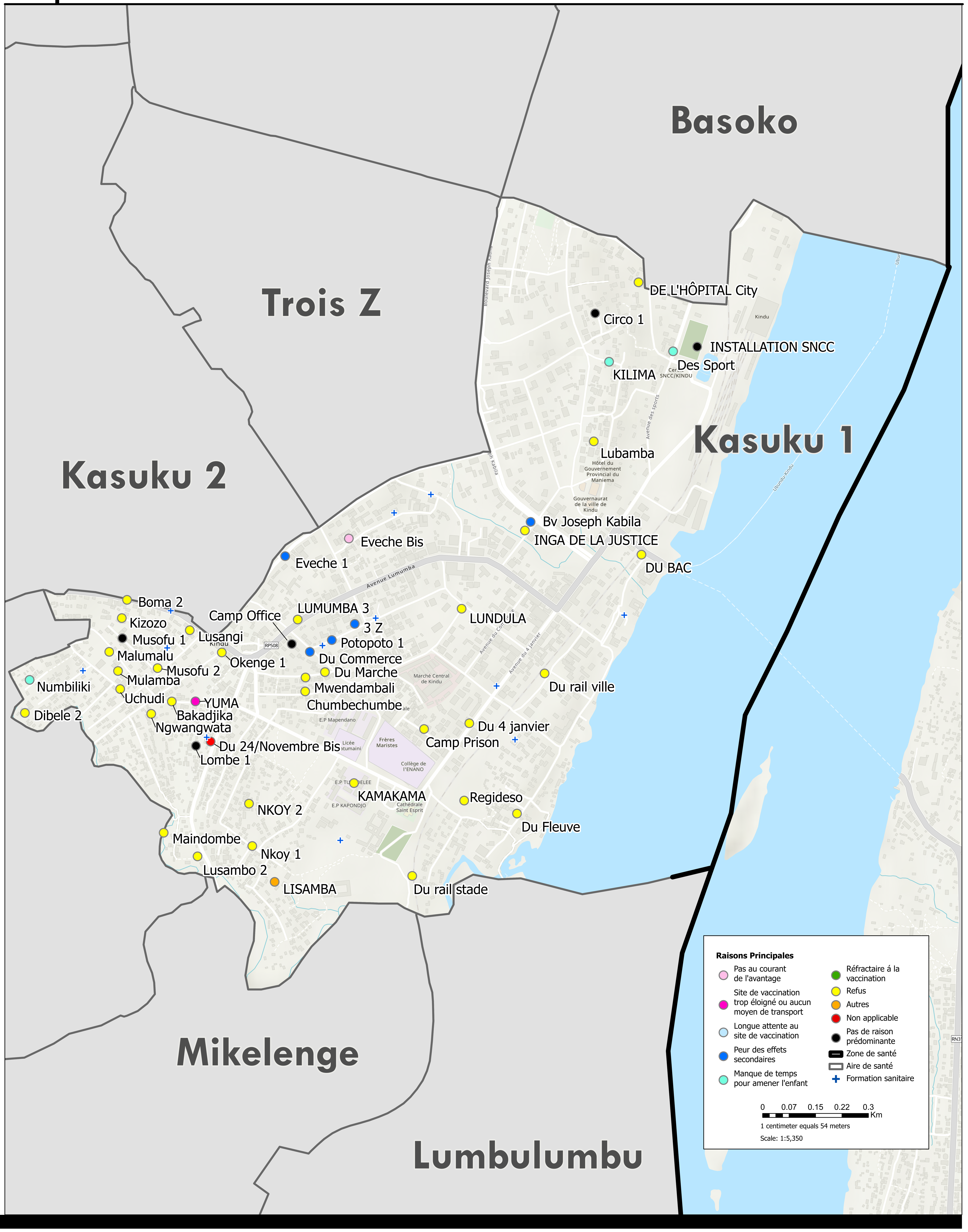

Centers for Disease Control and Prevention
Agency for Toxic Substances and Disease Registry

Clause de non-responsabilité: Les limites et les noms indiqués et les désignations utilisées sur cette carte n'impliquent l'expression d'aucune opinion de la part de la CDC concernant le statut juridique de tout pays, territoire, ville ou zone administrative ou de ses autorités, ou concernant la délimitation de ses frontières ou limites.

Geospatial Research, Analysis, and Services Program

SOURCES DE DONNÉES: PNLP, DSNIS, CDC, CIESIN, GRID3, OSM
